# Vulnerable newborn types: analysis of subnational, population‐based birth cohorts for 541 285 live births in 23 countries, 2000–2021

**DOI:** 10.1111/1471-0528.17510

**Published:** 2023-05-08

**Authors:** D. J. Erchick, E. A. Hazel, J. Katz, A. C. C. Lee, M. Diaz, L. S. F. Wu, S. Yoshida, R. Bahl, C. Grandi, A. B. Labrique, M. Rashid, S. Ahmed, A. D. Roy, R. Haque, S. Shaikh, A. H. Baqui, S. K. Saha, R. Khanam, S. Rahman, R. Shapiro, R. Zash, M. F. Silveira, R. Buffarini, P. Kolsteren, C. Lachat, L. Huybregts, D. Roberfroid, L. Zeng, Z. Zhu, J. He, X. Qiu, S. H. Gebreyesus, K. Tesfamariam, D. Bekele, G. Chan, E. Baye, F. Workneh, K. P. Asante, E. B. Kaali, S. Adu‐Afarwuah, K. G. Dewey, S. Gyaase, B. J. Wylie, B. R. Kirkwood, A. Manu, R. D. Thulasiraj, J. Tielsch, R. Chowdhury, S. Taneja, G. R. Babu, P. Shriyan, P. Ashorn, K. Maleta, U. Ashorn, C. Mangani, S. Acevedo‐Gallegos, M. J. Rodriguez‐Sibaja, S. K. Khatry, S. C. LeClerq, L. C. Mullany, F. Jehan, M. Ilyas, S. J. Rogerson, H. W. Unger, R. Ghosh, S. Musange, V. Ramokolo, W. Zembe‐Mkabile, M. Lazzerini, M. Rishard, D. Wang, W. W. Fawzi, D. T. R. Minja, C. Schmiegelow, H. Masanja, E. Smith, J. P. A. Lusingu, O. A. Msemo, F. M. Kabole, S. N. Slim, P. Keentupthai, A. Mongkolchati, R. Kajubi, A. Kakuru, P. Waiswa, D. Walker, D. H. Hamer, K. E. A. Semrau, E. B. Chaponda, R. M. Chico, B. Banda, K. Musokotwane, A. Manasyan, J. M. Pry, B. Chasekwa, J. Humphrey, R. E. Black, the Subnational Vulnerable Newborn Prevalence Collaborative Group and Vulnerable Newborn Measurement Core Group, Hasmot Ali, Parul Christian, Rolf D. W. Klemm, Alan B. Massie, Maithili Mitra, Sucheta Mehra, Kerry J. Schulze, Abu Ahmed Shamim, Alfred Sommer, MD. Barkat Ullah, Keith P. West, Nazma Begum, Nabidul Haque Chowdhury, Md. Shafiqul Islam, Dipak Kumar Mitra, Abdul Quaiyum, Modiegi Diseko, Joseph Makhema, Yue Cheng, Yixin Guo, Shanshan Yuan, Meselech Roro, Bilal Shikur, Frederick Goddard, Sebastien Haneuse, Bezawit Hunegnaw, Yemane Berhane, Alemayehu Worku, Seyram Kaali, Charles D. Arnold, Darby Jack, Seeba Amenga‐Etego, Lisa Hurt, Caitlin Shannon, Seyi Soremekun, Nita Bhandari, Jose Martines, Sarmila Mazumder, Yamuna Ana, R Deepa, Lotta Hallamaa, Juha Pyykkö, Mario I. Lumbreras‐Marquez, Claudia E. Mendoza‐Carrera, Atiya Hussain, Muhammad Karim, Farzana Kausar, Usma Mehmood, Naila Nadeem, Muhammad Imran Nisar, Muhammad Sajid, Ivo Mueller, Maria Ome‐Kaius, Elizabeth Butrick, Felix Sayinzoga, Ilaria Mariani, Willy Urassa, Thor Theander, Phillippe Deloron, Birgitte Bruun Nielsen, Alfa Muhihi, Ramadhani Abdallah Noor, Ib Bygbjerg, Sofie Lykke Moeller, Fahad Aftab, Said M. Ali, Pratibha Dhingra, Usha Dhingra, Arup Dutta, Sunil Sazawal, Atifa Suleiman, Mohammed Mohammed, Saikat Deb, Moses R. Kamya, Miriam Nakalembe, Jude Mulowooz, Nicole Santos, Godfrey Biemba, Julie M. Herlihy, Reuben K. Mbewe, Fern Mweena, Kojo Yeboah‐Antwi, Jane Bruce, Daniel Chandramohan, Andrew Prendergast, Joy E. Lawn, Hannah Blencowe, Eric Ohuma, Yemi Okwaraji, Judith Yargawa, Ellen Bradley, Joanne Katz

**Affiliations:** ^1^ Department of International Health Johns Hopkins Bloomberg School of Public Health Baltimore Maryland USA; ^2^ Department of Pediatric Newborn Medicine Brigham and Women's Hospital, Harvard Medical School Boston Massachusetts USA; ^3^ Department of Maternal, Newborn, Child and Adolescent Health and Ageing World Health Organization Geneva Switzerland; ^4^ Argentine Society of Paediatrics Ciudad Autónoma de Buenos Aires Argentina; ^5^ IntraHealth International Dhaka Bangladesh; ^6^ Projahnmo Research Foundation Dhaka Bangladesh; ^7^ JiVitA Maternal and Child Health Research Project Rangpur Bangladesh; ^8^ Child Health Research Foundation Dhaka Bangladesh; ^9^ Department of Women's and Children's Health Uppsala University Uppsala Sweden; ^10^ Department of Immunology and Infectious Diseases Harvard T.H. Chan School of Public Health Boston Massachusetts USA; ^11^ Beth Israel Deaconess Medical Center Boston Massachusetts USA; ^12^ Postgraduate Program in Epidemiology Federal University of Pelotas Pelotas Brazil; ^13^ Department of Food Technology, Safety and Health Ghent University Ghent Belgium; ^14^ Poverty, Health and Nutrition Division International Food Policy Research Institute Washington DC USA; ^15^ Medicine Department, Faculty of Medicine University of Namur Namur Belgium; ^16^ Department of Epidemiology and Biostatistics School of Public Health, Xi'an Jiaotong University Health Science Center Xi'an China; ^17^ Division of Birth Cohort Study, Guangzhou Women and Children's Medical Centre Guangzhou Medical University Guangzhou China; ^18^ Department of Nutrition and Dietetics, School of Public Health Addis Ababa University Addis Ababa Ethiopia; ^19^ Department of Food Technology, Safety and Health Faculty of Bioscience Engineering, Ghent University Ghent Belgium; ^20^ Department of Obstetrics and Gynecology Harvard T.H. Chan School of Public Health Boston Massachusetts USA; ^21^ Department of Obstetrics and Gynecology St. Paul's Hospital Millennium Medical College Addis Ababa Ethiopia; ^22^ Department of Epidemiology Harvard T.H. Chan School of Public Health Boston Massachusetts USA; ^23^ Department of Pediatrics Boston Children's Hospital, Harvard Medical School Boston Massachusetts USA; ^24^ Addis Continental Institute of Public Health Addis Ababa Ethiopia; ^25^ Kintampo Health Research Centre Research and Development Division Kintampo Ghana; ^26^ Department of Nutrition and Food Science University of Ghana Accra Ghana; ^27^ Institute for Global Nutrition, Department of Nutrition University of California Davis California USA; ^28^ Department of Statistics Kintampo Health Research Centre Kintampo Ghana; ^29^ Department of Obstetrics and Gynecology Columbia University Medical Center New York New York USA; ^30^ Epidemiology and Population Health London School of Hygiene & Tropical Medicine London UK; ^31^ University of Ghana School of Public Health Accra Ghana; ^32^ Aravind Eye Care System Madurai India; ^33^ Department of Global Health, Milken Institute School of Public Health George Washington University Washington DC USA; ^34^ Centre for Health Research and Development Society for Applied Studies Delhi India; ^35^ Department of Population Medicine College of Medicine QU Health Qatar University Doha Qatar; ^36^ Indian Institute of Public Health, Public Health Foundation of India Bengaluru India; ^37^ Center for Child, Adolescent and Maternal Health Research, Faculty of Medicine and Health Technology Tampere University and Tampere University Hospital Tampere Finland; ^38^ School of Global and Public Health Kamuzu University of Health Sciences Blantyre Malawi; ^39^ National Institute of Perinatology Maternal‐Fetal Medicine Department Mexico City Mexico; ^40^ Nepal Nutrition Intervention Project – Sarlahi (NNIPS) Kathmandu Nepal; ^41^ Department of Paediatrics and Child Health The Aga Khan University Karachi Pakistan; ^42^ The Aga Khan University Karachi Pakistan; ^43^ Department of Infectious Diseases University of Melbourne, Doherty Institute Melbourne Victoria Australia; ^44^ Menzies School of Health Research Charles Darwin University Darwin Northern Territory Australia; ^45^ Institute for Global Health Sciences, Department of Epidemiology and Biostatistics University of California San Francisco San Francisco California USA; ^46^ School of Public Health, College of Medicine and Health Sciences University of Rwanda Kigali Rwanda; ^47^ HIV and Other Infectious Diseases Research Unit South African Medical Research Council Cape Town South Africa; ^48^ Gertrude H Sergievsky Center Vagelos College of Physicians and Surgeons, Columbia University Irving Medical Center New York New York USA; ^49^ Health Systems Research Unit South African Medical Research Council Cape Town South Africa; ^50^ College Graduate of Studies University of South Africa Johannesburg South Africa; ^51^ Institute for Maternal and Child Health – IRCCS ‘Burlo Garofolo’, WHO Collaborating Centre for Maternal and Child Health Trieste Italy; ^52^ University Obstetrics Unit De Soysa Hospital for Women Colombo Sri Lanka; ^53^ Department of Obstetrics & Gynaecology University of Colombo Colombo Sri Lanka; ^54^ Department of Global and Community Health College of Public Health, George Mason University Fairfax Virginia USA; ^55^ Department of Global Health and Population Harvard T.H. Chan School of Public Health Boston Massachusetts USA; ^56^ National Institute for Medical Research, Tanga Centre Tanga Tanzania; ^57^ Centre for Medical Parasitology, Department for Immunology and Microbiology Faculty of Health and Medical Sciences, University of Copenhagen Copenhagen Denmark; ^58^ Ifakara Health Institute Dar es Salaam Tanzania; ^59^ Department of Global Health Milken Institute School of Public Health Washington DC USA; ^60^ National Institute for Medical Research Dar es Salaam Tanzania; ^61^ Ministry of Health Zanzibar Zanzibar Tanzania; ^62^ College of Medicine and Public Health Ubon Ratchathani University Ubon Ratchathani Thailand; ^63^ ASEAN Institute for Health Development Mahidol University Salaya Thailand; ^64^ Infectious Diseases Research Collaboration Kampala Uganda; ^65^ Department of Health Policy Planning and Management Makerere University School of Public Health, New Mulago Hospital Complex Kampala Uganda; ^66^ Division of Global Health, Department of Public Health Sciences Karolinska Institutet Stockholm Sweden; ^67^ Institute for Global Health Sciences and Department of Obstetrics and Gynecology University of California San Francisco San Francisco California USA; ^68^ Department of Global Health Boston University School of Public Health Boston Massachusetts USA; ^69^ Section of Infectious Diseases, Department of Medicine Boston University Chobanian & Avedisian School of Medicine Boston Massachusetts USA; ^70^ Ariadne Labs Brigham and Women's Hospital and Harvard T.H. Chan School of Public Health Boston Massachusetts USA; ^71^ Division of Global Health Equity & Department of Medicine Brigham and Women's Hospital Boston Massachusetts USA; ^72^ Department of Biological Sciences School of Natural Sciences, University of Zambia Lusaka Zambia; ^73^ Department of Disease Control, Faculty of Infectious & Tropical Diseases London School of Hygiene & Tropical Medicine London UK; ^74^ Research Unit for Environmental Sciences and Management North‐West University Potchefstroom South Africa; ^75^ Health Specialist PMTCT and Pediatric AIDS UNICEF Lusaka Zambia; ^76^ University of Alabama at Birmingham Birmingham Alabama USA; ^77^ Centre for Infectious Disease Research in Zambia Lusaka Zambia; ^78^ Zvitambo Institute for Maternal and Child Health Research Harare Zimbabwe

**Keywords:** low birthweight, newborn, preterm birth, small for gestational age

## Abstract

**Objective:**

To examine prevalence of novel newborn types among 541 285 live births in 23 countries from 2000 to 2021.

**Design:**

Descriptive multi‐country secondary data analysis.

**Setting:**

Subnational, population‐based birth cohort studies (*n* = 45) in 23 low‐ and middle‐income countries (LMICs) spanning 2000–2021.

**Population:**

Liveborn infants.

**Methods:**

Subnational, population‐based studies with high‐quality birth outcome data from LMICs were invited to join the Vulnerable Newborn Measurement Collaboration. We defined distinct newborn types using gestational age (preterm [PT], term [T]), birthweight for gestational age using INTERGROWTH‐21st standards (small for gestational age [SGA], appropriate for gestational age [AGA] or large for gestational age [LGA]), and birthweight (low birthweight, LBW [<2500 g], nonLBW) as ten types (using all three outcomes), six types (by excluding the birthweight categorisation), and four types (by collapsing the AGA and LGA categories). We defined small types as those with at least one classification of LBW, PT or SGA. We presented study characteristics, participant characteristics, data missingness, and prevalence of newborn types by region and study.

**Results:**

Among 541 285 live births, 476 939 (88.1%) had non‐missing and plausible values for gestational age, birthweight and sex required to construct the newborn types. The median prevalences of ten types across studies were T+AGA+nonLBW (58.0%), T+LGA+nonLBW (3.3%), T+AGA+LBW (0.5%), T+SGA+nonLBW (14.2%), T+SGA+LBW (7.1%), PT+LGA+nonLBW (1.6%), PT+LGA+LBW (0.2%), PT+AGA+nonLBW (3.7%), PT+AGA+LBW (3.6%) and PT+SGA+LBW (1.0%). The median prevalence of small types (six types, 37.6%) varied across studies and within regions and was higher in Southern Asia (52.4%) than in Sub‐Saharan Africa (34.9%).

**Conclusions:**

Further investigation is needed to describe the mortality risks associated with newborn types and understand the implications of this framework for local targeting of interventions to prevent adverse pregnancy outcomes in LMICs.

## 1 INTRODUCTION

In 2020, more than 5 million children under the age of 5 died, with nearly half (47%), occurring within the first 28 days after birth (neonatal deaths).^1^ In 2014, the Every Newborn Action Plan called for the reduction of neonatal deaths to 12 per 1000 live births, a threshold later incorporated into the United Nations Sustainable Development Goal (SDG) 3 with a target date of 2030.^2^, ^3^, ^4^ Globally, approximately three‐quarters of neonatal deaths occur in the first week after birth, and one‐third on the first day, caused mainly by preterm birth, intrapartum‐related complications and infections.^5^ Accelerating reductions in neonatal mortality to meet global and national targets will require understanding of the conditions before and at the time of birth that contribute to the risk of mortality.

Low birthweight (LBW), defined as <2500 g, is a commonly used indicator associated with neonatal mortality and morbidity, inadequate infant/child growth, and adverse health outcomes later in life.^6^, ^7^ LBW is caused by preterm birth (PT) or fetal growth restriction (FGR), which is defined by a metric at birth, small for gestational age (SGA), often <10th centile of expected birthweight for gestational age and sex. The SDGs and Global Nutrition Plan include a target of 30% reduction in the number of infants born LBW by 2025, although there has been little progress towards this goal, which was recently extended to 2030.^8^ In 2015, 20.5 million liveborn infants (15.5% of births) were estimated to be born LBW, with a prevalence of 26.4% in Southern Asia and 14.0% in Sub‐Saharan Africa.^9^ Of 18 million estimated LBW live births in low‐ and middle‐income countries (LMICs) in 2010, 41% were preterm and 59% were term‐SGA.^10^ There were also 6.3 million estimated preterm births and 19.0 million estimated term SGA births that were non‐low birthweight (nonLBW) but still vulnerable to excess mortality and other adverse outcomes.^10^, ^11^

Currently, global, regional and national estimates are produced separately for LBW and PT births. Yet these separate, dichotomous classifications are overlapping and not sufficiently granular to quantify prevalence and mortality risk for specific types of vulnerable newborns. Further, they obscure the important contribution of SGA, including term‐SGA infants that are nonLBW.^12^ Existing estimates also do not consider large for gestational age (LGA) infants, who may be at increased mortality risk, mainly due to intrapartum‐related complications and neonatal hypoglycaemia, and have a separate set of risk factors and long‐term health complications.^13^ Together, PT/T, SGA/appropriate for gestational age (AGA)/LGA, and LBW/nonLBW combine to create 12 mutually exclusive types, two of which are not biologically plausible (PT+SGA+nonLBW and T+LGA+LBW). There are several possible approaches to the classification of vulnerable newborn types, including retaining more detail as ten groups, or simplifying by collapsing some groups; for example, by categorising types in six groups defined by P/T and SGA/AGA/LGA or four groups defined by P/T and SGA/AGA. Another classification is ‘small types’, defined as classifications of LBW, PT or SGA (one of the ‘ten’ types classification) or PT or SGA (one of the ‘six’ or ‘four’ types classification) versus non‐small types. Notably, in all of these classifications, we do not distinguish between term versus post‐term infants or appropriate birthweight versus high birthweight infants because of the small prevalence of the post‐term and high birthweight types.

The categorisation of newborn types should be considered in the context of biological and epidemiological considerations, and any approach has a broad range of implications for research, policy and programmes for maternal, fetal and newborn health. A 2020 *Lancet* comment called for an effort to consider the prevalence and mortality risk of newborn types and describe their risk factors, effective interventions and financial considerations.^14^ The Vulnerable Newborn Measurement Collaboration (VNMC) is a multi‐country partnership to close this measurement gap by applying standard definitions and undertaking analyses to generate prevalence, mortality risk and population attributable risk for newborn types, and to inform improved data quality and use in all regions of the world. This paper is part of a journal supplement that will provide description, analysis and novel insights for improving measurement and capture of detailed newborn types within national and subnational data. This journal supplement describes individual‐level data from 23 national datasets (~165 million live births) and 45 studies in 23 countries (~0.5 million live births) that were used as inputs to model the national and regional prevalence for three mutually exclusive small vulnerable newborn types not including LBW classification, preterm PT+nonSGA, T+SGA and PT+SGA in 2020, recently published in the *Lancet* Small Vulnerable Newborn series.^15^

**FIGURE 1:**
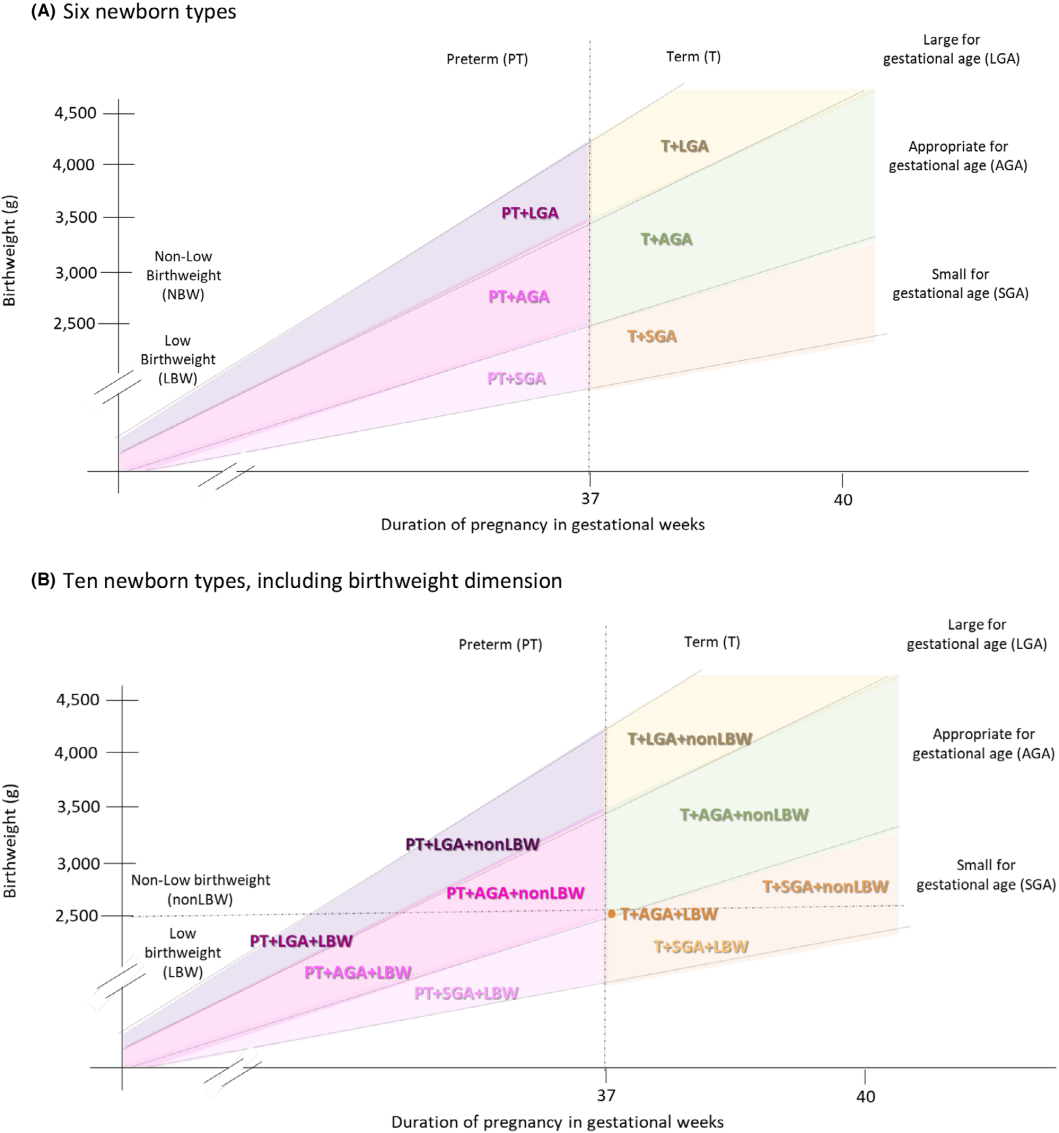
Overview of newborn types based on gestational age, size for gestational age and birthweight. (A) Six newborn types. (B) Ten newborn types, including birthweight dimension. This figure illustrates the six newborn types and more granular expansion of these types adding the birthweight dimension. Original newborn types proposed by Ashorn et al. are shown in Appendix S4.

This paper aims to describe the prevalence of newborn types among live births in LMICs using data from subnational, population‐based randomised trials and prospective cohort studies (Figures 1 and S4). We also assessed the data quality of included studies, specifically study population, recruitment protocols, missing gestational age, birthweight, sex data, implausible values and data heaping. Lastly, we consider the benefits of presenting the prevalence of the newborn types using different mutually exclusive categorisations, including ten, six or four groups, and potential for future work to align aetiologies, risk factors, and mortality and morbidity risks more clearly with specific birth outcomes (Table 1).

**TABLE 1:** Key findings.

1. What was known?
Currently, global estimates are produced separately for low birth weight and preterm birth. Yet these separate, dichotomous classifications are overlapping and not sufficiently granular to qsuantify prevalence, mortality risk, risk factors and aetiologies for specific types of vulnerable newborns. Further, they obscure the important contribution of SGA, including term‐SGA infants that are nonLBW, and large for gestational age infants.
2. What was done that is new?
Our study is the first multi‐country analysis to present detailed newborn type prevalences for a large number of low‐ and middle‐income countries (LMICs). We conducted a systematic search to identify 45 studies from 23 LMICs and 541 285 live births with high‐quality population‐based data on pregnancy outcomes.
We defined and described prevalence for newborn types categorised by preterm (PT) and term (T), size for gestational age (small (SGA), appropriate (AGA) and large (LGA)) and low birthweight (LBW) and non‐LBW (nonLBW).
3. What was found?
*Six types*: The median prevalences of six types across studies were T + AGA (58.5%), T + LGA (3.3%), T + SGA (21.9%), PT + LGA (1.7%), PT + AGA (7.4%), and PT + SGA (1.0%). Southern Asia, compared to Sub‐Saharan Africa, had a higher prevalence of T + SGA (medians: 40.6% vs. 19.3%), PT + SGA (medians: 2.4% vs. 0.8%), and PT + AGA (medians: 9.4% vs. 6.3%), and lower prevalence of T + AGA (medians: 46.8% vs. 62.4%) and both T+LGA (medians: 0.8% vs. 4.3%) and PT + LGA (medians: 1.7% vs. 3.0%).
*Small types*: The overall median prevalence of small types (six categories) was 37.6% and ranged across studies from 12.9% to 80.0%. Median small type prevalence was 52.4% for Southern Asia (range: 29.8% to 80.0%) and 34.9% for Sub‐Saharan Africa (range: 14.4% to 48.0%).
4. What next?
*Action in preventive programmes*: The categorisation of newborn types should be considered in context of biological and epidemiologic considerations, including data on associated mortality risks. This approach has a broad range of implications for research, policy, and programs for maternal, fetal, and newborn health.
*Research gaps*: Prevalence of vulnerable newborn types are reliant upon high‐quality, population‐based data. Efforts to strengthen routine data systems for collection and tracking of gestational age and birthweight data are critical to development of targeted preventive and therapeutic interventions for vulnerable newborns.

## 2 METHODS

### 2.1 Dataset identification

**FIGURE 2:**
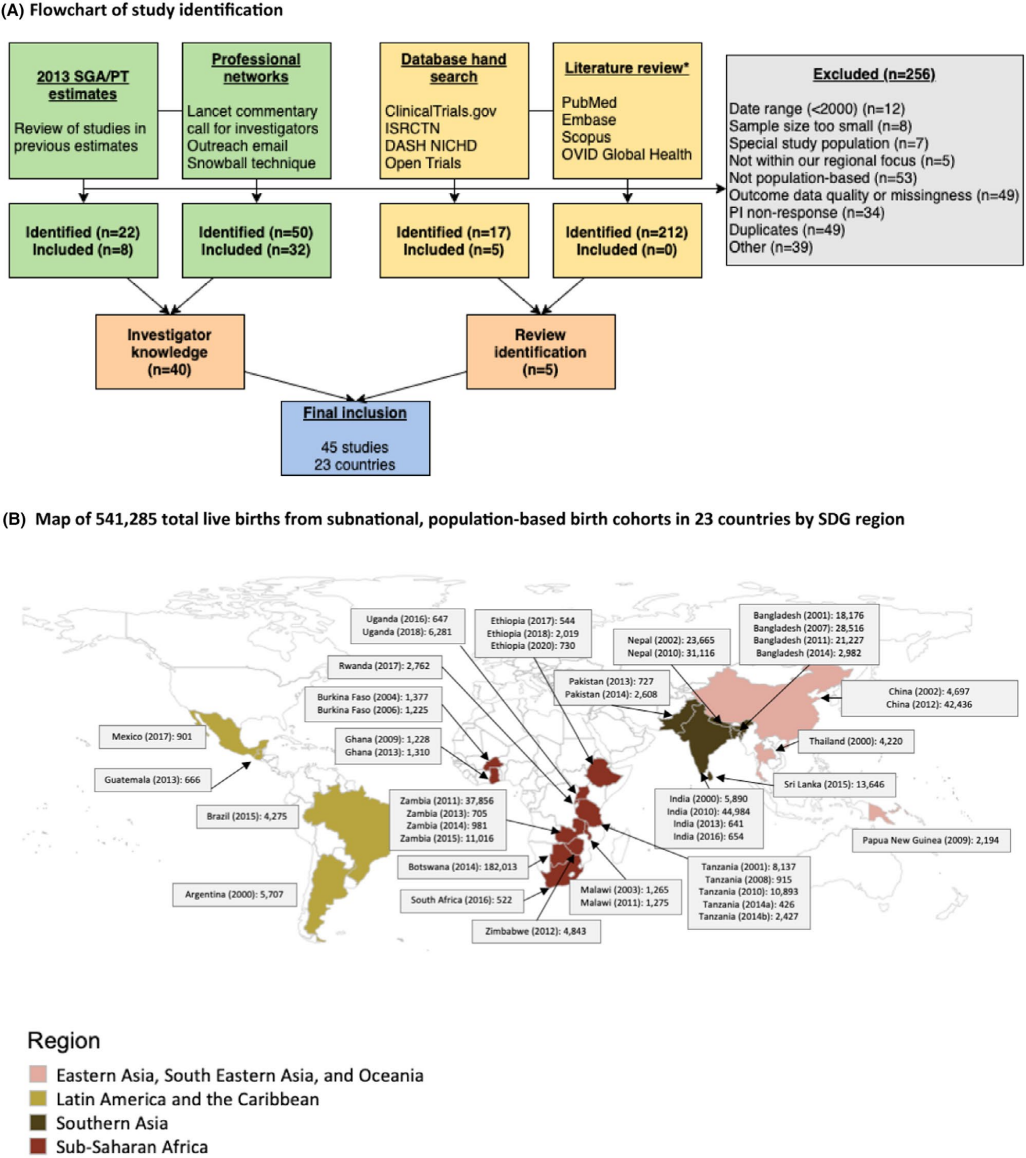
Study identification and location. (A) Flowchart of study identification. *The studies listed as ‘identified’ for the literature review are the articles that underwent full‐text extraction. The full methodology for the literature review, including the total results, title/abstract screening and duplicate removals, is in Appendices S1–S8. (B) Map of 541 285 total live births from subnational, population‐based birth cohorts in 23 countries by SDG region.

We identified datasets through multiple sources, including literature review, hand‐search of online databases, outreach through professional networks, and investigator knowledge (Figure 2A, Appendices S1, S2 and S5) from LMICs in four UN SDG Regions (Sub‐Saharan Africa; Southern Asia, Eastern Asia, South Eastern Asia and Oceania (combined as one region); and Latin America and the Caribbean). We searched MEDLINE, Embase, Scopus and OVID Global Health to identify potential studies to include in our analysis. This search was restricted to articles published between 1 January 2000, and 15 March 2021, and accepted publications in any language. We selected an approximately 20‐year period to ensure that a sufficient number of high‐quality studies meeting our inclusion/exclusion criteria in four UN SDG Regions would be available for analysis. Search terms included ‘small for gestational age’, and ‘low birthweight’, and ‘preterm’, and ‘low‐ and middle‐income countries’, with variations on these phrases. We conducted a hand search of clinicaltrials.gov, ISRCTN, Open Trials and medRxiv to explore relevant studies. We searched bibliographies of several reviews utilising pregnancy or birth cohorts in LMICs, including prior analyses by investigators.^10^, ^11^ Additionally, authors of the 2020 *Lancet* comment called for investigators and national authorities with suitable data to participate in a collaborative analysis effort.^14^ To extend the reach of this call, our team shared an outreach email and an online survey with investigators in the maternal and child health field with the request for collaboration.

### 2.2 Dataset inclusion and exclusion criteria

We aimed to include studies with high‐quality measurement of gestational age, birthweight and sex that identified and enrolled pregnancies or live births through a population‐based framework, representing all deliveries in a defined geographical area, whether facility‐based or home‐based. We considered facility‐based studies to be population‐based in settings where approximately 80% or more of births occur in facilities. For studies that recruited at antenatal (ANC) clinics, we defined population‐based as settings where about 90% of women received at least one ANC visit. We excluded studies for the following reasons: sample size of <300 live births; participant recruitment did not meet our population‐based criteria; gestational age was not assessed through ultrasound examination or last menstrual period (LMP) methods; birthweight was not collected <72 hours after delivery; missingness >30% among live births of complete gestational age, birthweight and sex information; or data collection before year 2000. We excluded stillbirths because few studies collected birthweight data on these outcomes. Newborns with lethal birth defects who were liveborn but died soon after birth were included.

### 2.3 Exposure definitions

Gestational age was categorised as PT <37 weeks (up to 36^+6^) or T ≥37 weeks (37^+0^ and above) using days or completed weeks (Appendix S3). Birthweight was categorised as LBW <2500 g or nonLBW ≥2500 g. SGA was defined as birthweight less than the 10th centile for exact gestational age in days by sex using the INTERGROWTH‐21st Standard.^16^ AGA was defined as greater than or equal to the 10th centile and less than the 90th centile. LGA was defined as greater than or equal to the 90th centile. For this analysis, our team modified the INTERGROWTH‐21st Standard by extrapolating the original equation for the range of gestational age down to 22^+0^ (previously 24^+0^) and up to 44^+6^ weeks (previously 42^+6^).^17^ This was done to allow us to include a wider range of gestational ages than the original INTERGROWTH‐21st standard provided at both the lower and upper ends of gestational age. The methods have been described elsewhere.^15^

Using PT/T, SGA/AGA/LGA and LBW/nonLBW, we constructed mutually exclusive sets of newborn types, including those with ten, six, four and small/non‐small types categories. The 10‐category types included T+AGA+nonLBW (reference), T+LGA+nonLBW, T+AGA+LBW, T+SGA+nonLBW, T+SGA+LBW, PT+LGA+nonLBW, PT+LGA+LBW, PT+AGA+nonLBW, PT+AGA+LBW and PT+SGA+LBW. The six‐category types included T+AGA (reference), T+LGA, T+SGA, PT+LGA, PT+AGA and PT+SGA. The four‐category types included T+AGA (reference), T+SGA, PT+AGA, and PT+SGA. We also defined non‐small types as the combination of T+AGA+nonLBW and T+LGA+nonLBW and small types as the combination of the remaining eight types. The small types were similarly constructed using the six‐ and four‐type categorisations, i.e. small types T+SGA, PT+LGA, PT+AGA and PT+SGA versus non‐small types T+AGA and T+LGA.

### 2.4 Analysis of individual datasets

We provided each principal investigator the option of conducting the analysis themselves or sharing the data with us for analysis. In cases where the principal investigators opted to perform their own analysis, we provided standard code in Stata (STATACORP) to clean their dataset and output the results. The same analysis was conducted on each dataset of live births. We selected inclusion and exclusion criteria for analysis of individual datasets and applied these consistently across papers in this journal supplement.

Among live births, we excluded individual observations missing gestational age, birthweight or sex data. We excluded individual observations with gestational age assessment collected through methods other than ultrasonography or LMP (e.g. fundal height) or birthweight collected >72 hours after delivery. We also excluded individual observations with gestational age values <22^+0^ or >44^+6^ weeks and birthweight values <250 or ≥6500 g. Finally, we considered as improbable and excluded gestational age and birthweight combinations where the birthweight was greater than five times the standard deviation from the mean weight for each gestational age week by sex.^16^ We retained multiple births in this analysis because we aimed to describe the prevalence of these small vulnerable newborn types among live births, regardless of the aetiologies of these adverse outcomes. Among randomised controlled trials included in this analysis, we retained all participants, regardless of their intervention assignment.

We calculated the prevalence of participant characteristics in each study, including maternal age, education, parity, delivery location, delivery type, infant sex and multiple births. We also calculated data quality indicators by study, including proportions of missing types overall and by each reason for missingness (e.g. missing gestational age or birthweight collected >72 hours). We calculated a birthweight heaping index by study as the number of live births reported at exactly 2500 g divided by the number of births with reported birthweight 249 g below (i.e. >2250 and <2500 g) and 249 g above (i.e. >2500 and <2750 g) this exact value (low values indicate better reporting practices and higher quality of data).^18^, ^19^ The distribution of birthweight and gestational age was assessed visually and by quantifying the proportion of births with birthweight <500 g, 1000 g or gestational age of ≤28^+6^ weeks.

### 2.5 Summary analysis

We present participant characteristics, data quality and missingness, and median prevalence of newborn types by ten groups, six groups, four groups and small/non‐small types individually for each study and region. We also presented small type prevalence overall and regionally by study time period (2000–2010 versus 2011–2021) and rural versus urban setting. The median, instead of the mean, was used for global and regional prevalences to avoid undue influence of larger studies on the results. The overall and regional newborn type prevalences presented in this study are intended only to summarise the data available from the participating research studies. They should not be interpreted as final regional prevalence estimates, which will be generated using the data presented in this study along with data from other sources through future efforts of the Vulnerable Newborn Measurement Collaboration team.

## 3 RESULTS

### 3.1 Study and participant characteristics

We included 45 datasets from 23 countries with 541 285 live births from Sub‐Saharan Africa (24 datasets, 11 countries); Southern Asia (13 datasets, five countries); Eastern Asia, South Eastern Asia and Oceania (combined as one region) (4 datasets, three countries); and Latin America and the Caribbean (4 datasets, four countries) (Table 2, Appendices S6a–S8). Studies had a median sample size of 2608 live births (range: 426–182 013) and were conducted in 23 countries with data collection spanning 2000 to 2021 (Figure 2B).

**TABLE 2:** Characteristics of 45 subnational, population‐based birth cohorts included in this analysis.

Study	Original cohort of live births (*n*)	Analysed cohort of live births (*n*)	Facility delivery (%)^a^	Multiple birth (%)^b^	Percent of live births in region (%)^c^	Percent of live births overall (%)	Data collection (years)	Setting	Primary study design	Population represented
Sub‐Saharan Africa (*n* = 24)	281 357	247 040								
Botswana (2014)	182 013	163 928	100	2.0	66.4	34.4	2014–2020	Multiple locations	Prospective observational cohort study using review of antenatal medical records	Facility‐based recruitment of pregnancies at govt. facilities countrywide
Burkina Faso (2004)	1337	1045	92.6	3.3	0.4	0.2	2004–2006	Rural Hounde	RCT of multiple micronutrient supplementation	Prospective, community‐based cohort
Burkina Faso (2006)	1225	1050	95.9	3.8	0.4	0.2	2006–2008	Rural Hounde	RCT of maternal fortified food supplementation	Prospective, community‐based cohort
Ethiopia (2017)	544	544	81.7	5.5	0.2	0.1	2017–2020	Urban and rural Butajira	Prospective observational cohort study	Population‐based recruitment of all pregnant women in study area
Ethiopia (2018)	2019	1424	84.2	3.6	0.6	0.3	2018–2021	Rural Amhara	Prospective observational cohort study	Population‐based recruitment of all pregnant women in study area
Ethiopia (2020)	730	556	87.1	0.0	0.2	0.1	2020–2021	Rural Amhara	Randomised pragmatic clinical effectiveness study	Facility‐based ANC clinic recruitment
Ghana (2009)	1228	1037	91.8	1.7	0.4	0.2	2009–2014	Urban Manya Krobo and Yilo Krobo districts	RCT of lipid‐based nutrient supplements	Facility‐based ANC clinic recruitment
Ghana (2013)	1310	1291	53.9	0.5	0.5	0.3	2013–2016	Rural Kintampo	Cluster randomised RCT of clean cooking interventions	Population based recruitment of all pregnant women in study area
Malawi (2003)	1265	1199	63.1	0.9	0.5	0.3	2003–2006	Rural Mangochi District	RCT of sulfadoxine‐pyrimethamine and azithromycin in pregnancy	Facility‐based ANC clinic recruitment of pregnancies
Malawi (2011)	1275	1074	89.2	1.7	0.4	0.2	2011–2012	Rural Mangochi District	RCT of lipid‐based nutrient supplement	Facility‐based ANC clinic recruitment of pregnancies
Rwanda (2017)	2762	2762	100	1.8	1.1	0.6	2017–2019	Urban and rural areas of five districts	Cluster RCT of group antenatal group	Facility‐based ANC clinic recruitment of pregnancies
South Africa (2016)	522	394	99.2	0.0	0.2	0.1	2016–2020	Peri‐Urban Langa Township	Evaluation of social protection programme	Facility‐based ANC clinic recruitment of pregnancies
Tanzania (2001)	8137	7630	99	3.7	3.1	1.6	2001–2004	Urban Dar es Salaam	RCT of multiple micronutrient supplementation	Facility‐based ANC clinic recruitment of pregnancies
Tanzania (2008)	915	818	89.5	3.7	0.3	0.2	2008–2010	Rural Korogwe	Prospective observational cohort study	Facility‐based recruitment, ANC clinics, community follow‐up
Tanzania (2010)	10 893	8309	85.8	3.4	3.4	1.7	2010–2013	Urban and rural Morogoro and Dar es Salaam	RCT of newborn Vitamin A supplementation	Facility‐based ANC clinic and labour ward recruitment of pregnancies
Tanzania (2014a)	426	407	91.4	5.9	0.2	0.1	2014–2016	Rural Korogwe	Prospective observational cohort study	Population‐based recruitment of women preconception or in 1st trimester
Tanzania (2014b)	2427	2319	99.8	3.8	0.9	0.5	2014–2018	Rural Pemba Island	Prospective observational cohort study	Population‐based recruitment of all pregnant women in study area
Uganda (2016)	647	635	90.4	0.0	0.3	0.1	2016–2018	Rural Busia District	RCT of intermittent preventive therapy for malaria in pregnancy	Facility‐based ANC clinic recruitment of pregnancies
Uganda (2018)	6281	6255	100	2.6	2.5	1.3	2018–2019	Rural Busoga Region	Quasi‐experimental pre‐post‐intervention study of midwife checklist	Facility‐based recruitment of women presenting to labour ward
Zambia (2011)	37 856	29 207	67.5	2.1	11.8	6.1	2011–2013	Urban and rural areas of Southern Province	RCT of chlorhexidine application for umbilical cord disinfection	Facility‐based ANC clinic recruitment of pregnancies
Zambia (2013)	705	703	96.4	0.0	0.3	0.1	2013–2014	Rural Nchelenge District	Prospective observational cohort study	Facility‐based ANC clinic recruitment of pregnancies
Zambia (2014)	981	762	97.6	0.8	0.3	0.2	2014–2018	Rural Southern Province	Prospective observational cohort study	Population‐based recruitment of all pregnant women in study area
Zambia (2015)	11 016	9509	100	2.1	3.8	2.0	2015–2017	Urban Lusaka	Prospective observational cohort study	Facility‐based recruitment of pregnancies
Zimbabwe (2012)	4843	4182	92.3	3.2	1.7	0.9	2012–2017	Rural Chirumanzu and Shurugwi Districts	Cluster RCT of WASH and improved infant and young child feeding intervention	Population‐based recruitment of all pregnant women in study area
Southern Asia (*n* = 13)	194 832	166 149								
Bangladesh (2001)	18 176	13 368	3.8	1.3	8.0	2.8	2001–2007	Rural Gaibandha and Rangpur	Cluster RCT of maternal Vitamin A supplementation	Population‐based recruitment of all pregnant women in study area
Bangladesh (2007)	28 516	20 501	8.8	1.2	12.3	4.3	2007–2012	Rural Gaibandha and Rangpur	Cluster RCT of antenatal multiple micronutrient	Population‐based recruitment of all pregnant women in study area
Bangladesh (2011)	21 227	18 007	7.2	1.6	10.8	3.8	2011–2013	Rural Sylhet	Prospective observational cohort study	Population‐based recruitment of all pregnant women in study area
Bangladesh (2014)	2982	2572	49	0.9	1.5	0.5	2014–2018	Rural Sylhet	Prospective observational cohort study	Population based recruitment of all pregnant women in study area
India (2000)	5890	4136	60.8	0.9	2.5	0.9	2000–2001	Rural Tamil Nadu	RCT of newborn Vitamin A supplementation	Population‐based recruitment of all pregnant women in study area
India (2010)	44 984	44 958	56.6	1.3	27.1	9.4	2010–2012	Rural Haryana	RCT of newborn Vitamin A supplementation	Population‐based recruitment of all pregnant women in study area
India (2013)	641	573	—	3.3	0.3	0.1	2013–2014	Rural Karnataka	RCT of timing of maternal nutrition intervention	Prospective recruitment of women preconception in the study area
India (2016)	654	653	100	0.0	0.4	0.1	2016–2017	Urban Bangalore	Prospective observational cohort study	Facility‐based ANC clinic recruitment of pregnancies
Nepal (2002)	23 665	21 383	6.9	1.3	12.9	4.5	2002–2006	Rural Sarlahi District	Cluster RCT of newborn skin‐umbilical cord cleansing with chlorhexidine	Population‐based recruitment of all pregnant women in study area
Nepal (2010)	31 116	23 568	53.6	1.3	14.2	4.9	2010–2017	Rural Sarlahi District	Cluster RCT of newborn massage with sunflower seed oil	Population‐based recruitment of all pregnant women in study area
Pakistan (2013)	727	640	—	0.9	0.4	0.1	2013–2014	Rural Sind Province	RCT of timing of maternal nutrition intervention	Prospective recruitment of women preconception in the study area
Pakistan (2014)	2608	2415	65	0.2	1.5	0.5	2014–2018	Peri‐Urban Karachi	Prospective observational cohort study	Population‐based recruitment of all pregnant women in study area
Sri Lanka (2015)	13 646	13 375	100	2.7	8.1	2.8	2015–2017	Colombo	Prospective observational cohort study	Facility‐based recruitment of pregnancies
Eastern Asia, South Eastern Asia, Oceania (*n* = 4)	53 547	52 344								
China (2002)	4697	4380	87.5	2.0	8.4	0.9	2002–2006	Rural Shaanxi Province	Cluster RCT of antenatal supplementation with micronutrients interventions	Population‐based recruitment of all pregnant women in study area
China (2012)	42 436	42 249	100	4.2	80.7	8.9	2012–2020	Urban Guangzhou	Prospective observational cohort study	Facility‐based ANC clinic recruitment
Papua New Guinea (2009)	2194	1871	100	0.0	3.6	0.4	2009–2012	Rural Madang Municipality	RCT of intermittent preventive treatment of malaria in pregnancy	Facility‐based ANC clinic recruitment of pregnancies
Thailand (2000)	4220	3844	99.9	1.4	7.3	0.8	2000–2002	Rural areas and urban Bangkok	Prospective observational cohort study	Longitudinal birth cohort of all births in 5 districts
Latin America and the Caribbean (*n* = 4)	11 549	11 406								
Argentina (2000)	5707	5698	99.9	1.8	50.0	1.2	2000–2001	Urban Buenos Aires	Prospective observational cohort study	Facility‐based recruitment
Brazil (2015)	4275	4257	—	2.5	37.3	0.9	2015	Urban Pelotas City	Prospective observational cohort study	Prospective, community‐based cohort of live births
Guatemala (2013)	666	565	—	0.7	5.0	0.1	2013–2014	Rural Chimaltenango	RCT of timing of maternal nutrition intervention	Prospective recruitment of women preconception in the study area
Mexico (2017)	901	886	100	0.0	7.8	0.2	2017–2019	Urban Mexico City	Retrospective observational cohort study using review of fetal growth charts	Facility‐based retrospective enrolment of pregnancies
Total	541 285	476 939								

^a^ Studies without individual level data on home or facility birth are marked as blank. Uganda 2016 had a majority (>90%) of births conducted in a facility without individual level data and is therefore marked as 100%.

^b^ The following studies excluded multiple births at enrolment or from the final analysis: Ethiopia (2020), India (2016), Mexico (2017), Papua New Guinea (2009), South Africa (2016), Uganda (2016) and Zambia (2013).

^c^ The number of included live births for each study as a proportion of the total live births globally or for each respective region (analysis cohort column) included in this analysis. Included live births are those with complete and plausible gestational age, birthweight and sex data that were used in the analysis for each study.

Approximately half (53.3%) of included studies were conducted in Sub‐Saharan Africa and over a quarter (28.9%) in Southern Asia. The Sub‐Saharan Africa region had a higher proportion of studies from the second half of these 20 years (70.8%) compared with Southern Asia (53.8%). In Sub‐Saharan Africa, a higher proportion of studies (45.8%) were conducted in primarily urban populations or multiple settings, as compared with Southern Asia, which included only three of 13 (23.1%) studies in such areas. The facility delivery rate was higher among studies in Sub‐Saharan Africa (median: 92.0%) than in Southern Asia (median: 53.6%) and deliveries were almost exclusively in facilities in the other two regions.

Young maternal age (<19 years) was most prevalent in the studies from Southern Asia (median: 25.0%) followed by Sub‐Saharan Africa (median: 18.1%), compared with the other two regions, which had medians between 10% and 15% (Appendix S6c). The proportion of primiparous women was higher in studies from Southern Asia (median: 29.8%) than Sub‐Saharan Africa (median: 21.1%). The caesarean delivery rate was lower in studies from Sub‐Saharan Africa (median: 6.0%) and Southern Asia (median: 5.8%) than in the other two regions, which each had medians over 25%. Seven studies excluded multiple births; the multiple birth rate among the other 38 studies ranged from 0.2% to 5.9% (median: 1.9%).

### 3.2 Data quality and missingness

Among all live births, 476 939 (88.1%) had non‐missing and plausible values for gestational age, birthweight and sex required to construct the newborn types for this analysis (Table 3, Appendix S6f). Overall missingness of the newborn type data was <10% for half of the studies (48.9%). Newborn type missingness among all live births was primarily driven by missing gestational age data (5.0%), birthweight exclusion due to collection >72 hours after delivery (3.4%) and missing birthweight data (2.7%). Most studies had <5% gestational age missingness (82.2%), <5% birthweight exclusion due to collection timing (73.3%) and <5% birthweight missingness (62.2%).

**TABLE 3:** Data quality and missingness in 541 285 total live births among 45 studies.

Region	Missing type^a^ ^,^ ^b^	Missing GA only	Missing BW only	Missing GA & BW	Missing sex	Birthweight collected >72 h	GA below 22 weeks	GA below 28 weeks	GA above 45 weeks	BW below 500 g	BW below 1000 g	BW above 6500 g	Improbable GA & BW combination
Total	64 346 (11.9)	26 882 (5.0)	14 825 (2.7)	1234 (0.2)	263 (0.0)	18 242 (3.4)	137 (0.0)	2791 (0.5)	3543 (0.7)	87 (0.0)	1684 (0.3)	15 (0.0)	108 (0.0)
Sub‐Saharan Africa	34 317 (12.2)	24 350 (8.7)	4899 (1.7)	951 (0.3)	159 (0.1)	1186 (0.4)	118 (0.0)	2081 (0.7)	2801 (1.0)	78 (0.0)	1437 (0.5)	7 (0.0)	71 (0.0)
Southern Asia	28 683 (14.7)	2043 (1.0)	9195 (4.7)	155 (0.1)	45 (0.0)	16 874 (8.7)	15 (0.0)	582 (0.3)	720 (0.4)	2 (0.0)	134 (0.1)	8 (0.0)	18 (0.0)
Eastern Asia, South Eastern Asia, and Oceania	1203 (2.2)	420 (0.8)	676 (1.3)	127 (0.2)	59 (0.1)	168 (0.3)	0 (0.0)	64 (0.1)	21 (0.0)	2 (0.0)	44 (0.1)	0 (0.0)	17 (0.0)
Latin America and the Caribbean	143 (1.2)	69 (0.6)	55 (0.5)	1 (0.0)	0 (0.0)	14 (0.1)	4 (0.0)	64 (0.6)	1 (0.0)	5 (0.0)	69 (0.6)	0 (0.0)	2 (0.0)

^a^ Missing type refers to the percent of live births missing a newborn type value for any reason.

^b^ Values are number (%).

One‐third of studies (16/45, 35.6%) exclusively used ultrasound dating to measure gestational age and a slightly lower proportion used only LMP dating (28.8%); the remainder relied on a mixture of the two approaches and/or best obstetric estimate, including ultrasound (Appendix S6d). Ultrasound dating was used exclusively in 41.7% of studies in Sub‐Saharan Africa compared with only three studies (23.1%) in Southern Asia. Among all live births pooled across studies, ultrasound and LMP dating were used roughly evenly among studies in Sub‐Saharan Africa (47.5% and 52.1%, respectively). LMP was much more heavily relied upon in studies from Southern Asia (10.3% and 89.3%, respectively). Nearly three‐quarters (33/45, 73.3%) of studies recorded gestational age in days and the remainder used completed weeks (12/45, 26.7%).

Across studies, the birthweight heaping index ranged from 0.1% to 49.3% (median: 5.5%). Over one‐third of studies (35.6%) had heaping indices >10%. Heaping was more common among studies in Sub‐Saharan Africa (median: 13.8%) than in Southern Asia (median: 3.0%) or the other regions. Half of studies in Sub‐Saharan Africa (13/24, 54.2%) and 15% of studies in Southern Asia (2/13, 15.4%) had heaping indices >10%.

### 3.3 Newborn type prevalences

Across studies, the median proportions of LBW, PT, SGA and LGA births were 13.3%, 13.0%, 23.2% and 5.1%, respectively (Appendix S6b,g). LBW, PT and SGA birth prevalences were highest among studies from Southern Asia (medians: 28.6%, 14.2% and 42.7%). Among studies from Sub‐Saharan Africa, PT prevalence (median: 13.5%) was similar to Southern Asia, but prevalence of SGA birth (median: 19.9%) and LBW birth (median: 10.0%) were lower than in Southern Asia. Median LGA birth prevalence was 8.6% in studies from Sub‐Saharan Africa and 2.8% in Southern Asia.

**FIGURE 3:**
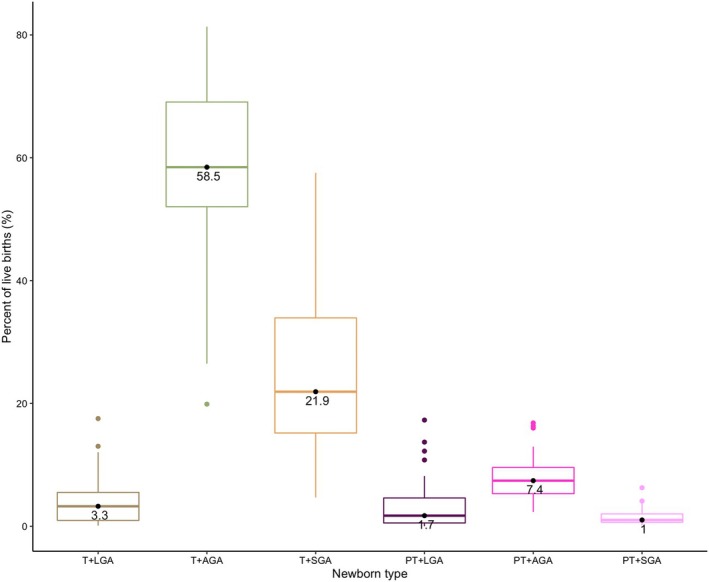
Median and interquartile range of subnational prevalence of six newborn types among 476 939 live births included in the analysis from 23 countries.

**FIGURE 4:**
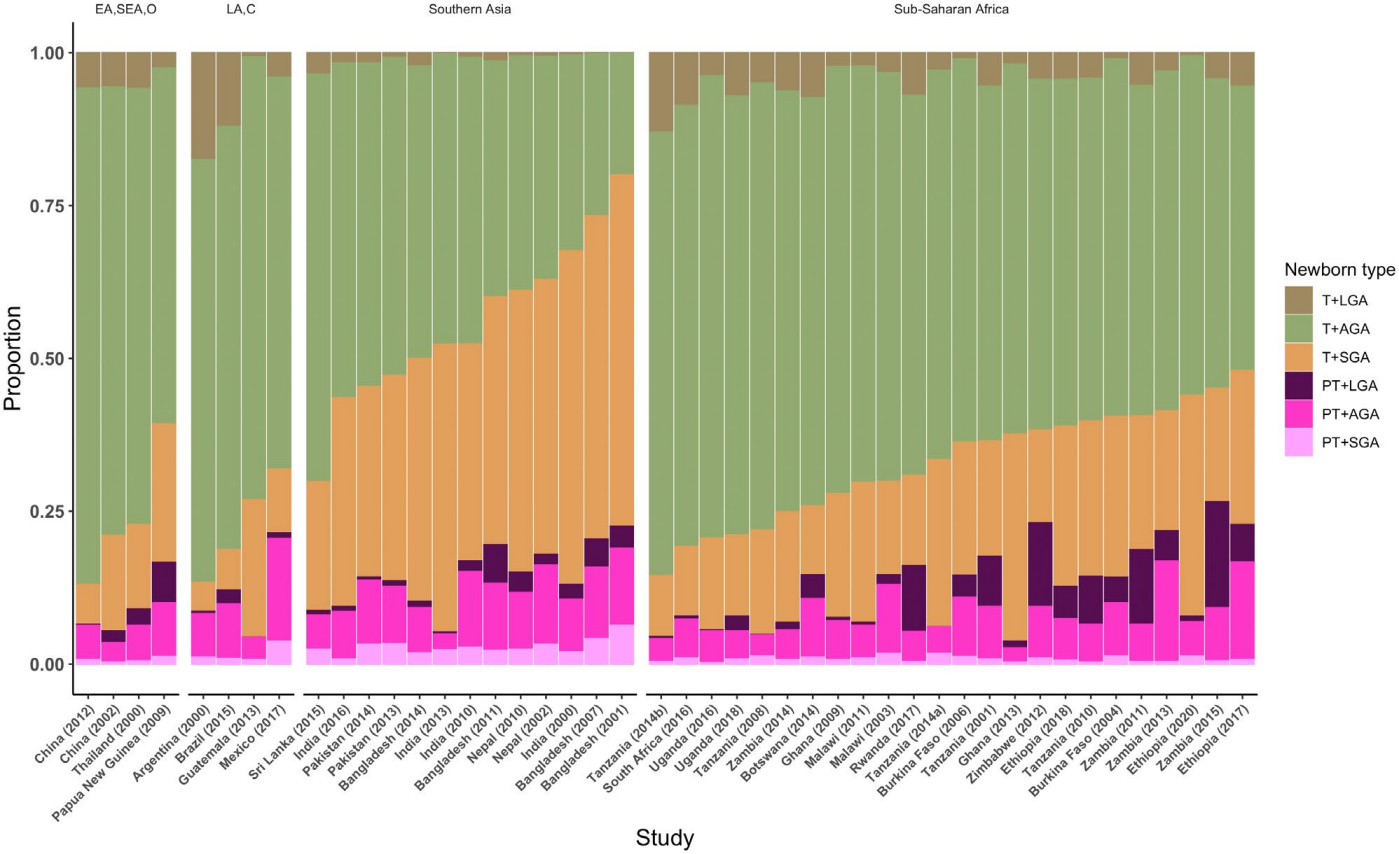
Prevalence of six newborn types among 476 939 live births included in the analysis by study and SDG region.

The median and interquartile range for the six newborn types is presented in Figure 3. The prevalence of six types by study is presented in Figure 4. Among the six types, Southern Asia had a higher prevalence of T‐SGA (medians: 40.6% versus 19.3%) and lower prevalence of T+AGA (medians: 46.8% versus 62.4%) compared with Sub‐Saharan Africa. The median prevalence of small types (six categories) was 37.6% (range across studies: 12.9% to 80.0%) overall, 52.4% for Southern Asia and 34.9% for Sub‐Saharan Africa.

The median prevalence of small types (six categories) comparing studies conducted between 2000–2010 (early) and 2011–2021 (late) was 39.3% versus 35.5%. In studies from Southern Asia, the median small type prevalence was 65.3% in the early period compared with 47.2% later. From 2001 to 2007, at the same community‐based field site in Bangladesh (JiVitA‐1, JiVitA‐3), the median prevalence of small types decreased by 8.4% (80.0–73.3%), due to reduction of both SGA (63.8–57.0%) and PT (22.5–20.4%). In studies from Sub‐Saharan Africa, the early and late period median prevalences were 36.4 and 33.4%, respectively.

Across studies, the median prevalence of small types (six categories) was highest in rural areas (39.3%), followed by mixed urban/rural areas (35.3%), and was lowest in urban areas (30.9%). In Southern Asia, the median small type prevalence was higher among studies conducted in rural areas (60.5%) versus urban areas/mixed areas (43.5%). In Sub‐Saharan Africa, the median small type prevalence was lower among studies conducted in rural (31.6%) versus urban/mixed areas (37.4%).

Additional results for the ten‐, six‐, four‐, and small versus non‐small types by region (Appendix S6e) and study (Appendix S6h–k) are presented in Appendices S1–S8.

## 4 DISCUSSION

We identified 45 subnational, population‐based studies among 23 countries in four geographical regions with high‐quality data on birth outcomes to serve as inputs for further analyses to describe the prevalence of and mortality risk associated with newborn types based upon gestational age, weight for gestational age and birthweight.

Our findings may suggest reasons to collapse types to fewer categories, such as by excluding the LBW/nonLBW classification. However, any decision to collapse types from ten to six or four categories should be taken in the context of mortality risks for each type, the focus of other papers in this supplement. The T+AGA+LBW type category is negligible in size (median: 0.5%), representing a narrow range of birthweights just below the 2500‐g cutoff among infants born around 37 weeks. Similarly, the PT+LGA+LBW type (median: 0.2%) represents a small unique group of infants with a gestational age range restricted to those born very or extremely preterm. Although a larger proportion of births, the PT+AGA+nonLBW and PT+AGA+LBW types both range across studies from very low (0.3% and 1.2%, respectively) to just over 10% (12.7% and 10.5%, respectively) prevalence. The T+SGA+nonLBW and T+SGA+LBW types also have a similar spread, with a somewhat wider range in prevalence, spanning 2.9% and 1.8%, respectively, to over a quarter (28.3%) and over a third (37.5%), respectively. Notably, the prevalences of small types, whether categorised using ten or six types, are similar.

There may be practical drawbacks to dropping the LBW/nonLBW classification, particularly in low‐resource settings. The LBW outcome is the simplest to measure accurately and is commonly used by health workers to inform the delivery of essential care of newborns. Overcoming this limitation may require improvements in access to high‐quality gestational age data through expanded use training of healthcare workers to provide early ultrasound gestational age assessment, higher coverage of early ANC visits and portable ultrasonography equipment. Further, a revised classification system could require understanding, across regions and countries, of how health workers and mothers understand aetiologies and mortality and morbidity risks for newborns, and evaluation of messages to convey appropriate information about care for specific newborn types.

Countries with national health information systems that collect representative, high‐quality and timely data on birth outcomes and death events are the ideal source for inputs to estimate the prevalence of newborn types.^20^ Our analysis focused on population‐based studies in LMICs, particularly in Sub‐Saharan Africa and Southern Asia, where such national systems are often incomplete.^21^ While not nationally representative, population‐based pregnancy or birth cohorts utilise prospective, systematic follow‐up that can generate high‐quality estimates of birth outcomes for a specific geographical area. We also included some studies from countries, mainly in Eastern Asia, South Eastern Asia, and Oceania and Latin America and Caribbean regions, that have systems with high‐quality national data that are more representative than our subnational data, but not all were available for analysis (e.g., People's Republic of China) to enable a more comprehensive analysis of newborn type prevalence, mortality and data quality in LMICs.

We observed substantial variation in newborn type distribution across included studies and within regions. A proportion of the variation in newborn type prevalence between studies in each region is likely attributable to heterogeneity in the risk factors and local determinants of these outcomes. For example, the prevalence of risk factors for poor birth outcomes differs between an urban population in Colombo (Sri Lanka 2015) and a rural community in Karnataka (India 2013), where the prevalence of small types was 30.8% and 53.1%, respectively, despite their proximity in time.^22^, ^23^ The prevalence of several important risk factors for PT and SGA and neonatal mortality are typically higher in rural areas, including young maternal age, short birth spacing, and maternal undernutrition.^24^, ^25^ Women in rural areas are also less likely to access complete antenatal care and deliver in a health facility.^26^ Over‐representation of rural locations in countries with increasing urban populations could impact estimates of newborn types. This may be most relevant for studies from Southern Asia, where the prevalence of SGA is highest,^27^ given that maternal undernutrition is a leading risk factor for this outcome.^28^

Data collection for included studies spanned 2000–2021. In that time, many countries achieved substantial improvements in maternal nutrition and access to health services, such as ANC and facility delivery, interventions known to reduce poor birth outcomes and increase newborn survival.^1^ Data were insufficient for a formal time‐trend analysis in our study. However, we observed that the highest proportions of poor birth outcomes and newborn types in the Southern Asia region were generally among studies conducted earlier in the 20‐year period. During this time, there were large shifts from home to facility delivery.^29^

Although we established strict inclusion and exclusion criteria for facility‐based studies, it is possible that such studies differentially enrolled newborn types, relative to studies that enrolled women at home or from multiple kinds of sites. Studies enrolling at only health facilities may miss more pregnancies that result in poor birth outcomes, stillbirth or neonatal death, as these events are more likely to occur among women without access to care, leading to underestimates of newborn types at higher risk of mortality.^30^ Alternatively, this bias could lead to overestimation of newborn types at higher risk of mortality in settings where a higher proportion of high‐risk births occur in facilities, as observed in rural Nepal.^31^ In facility settings, weight is likely measured closer to the time of delivery, providing more accurate estimates of birthweight than in non‐facility‐based studies, where delays in reaching participant homes could lead to lower or higher weights depending on the exact timing of the measurement because of infant weight changes in the first 72 hours after birth.

Similarly, although meeting our inclusion and exclusion criteria, some studies had many observations excluded due to missing birthweights or weights taken >72 hours. Observations with missing weights may be more likely to occur among preterm or small infants with higher risk of mortality, especially early mortality in home deliveries, presenting bias for prevalence and associated mortality risk for the newborn types.^9^ Early ultrasound, which is recommended as the gold standard for pregnancy dating, is also more commonly done in settings that have a high prevalence of facility deliveries. The LMP method has been associated with poor precision and biases that can influence estimates of gestational age, relative to ultrasound dating, especially maternal recall which can lead to overestimation and underestimation of the prevalence of preterm birth.^32^, ^33^, ^34^ Somewhat mitigating this concern is that many of the community‐based studies used active surveillance for pregnancies and early pregnancy testing, allowing LMP assessment with a short recall period.

Our study had limitations. We only identified data from a small group of countries within each region, which are unlikely to be representative of the entire region. Further, there were insufficient numbers of studies to sub‐divide the regions we selected, potentially masking important differences among studies between smaller geographical areas, such as western, eastern, southern or central sub‐regions of Sub‐Saharan Africa. Population‐based studies that are conducted in limited geographical areas, may not necessarily represent the national population. Investments in national data systems to improve data coverage and quality for vital events and birth outcomes would greatly benefit efforts to describe detailed newborn types, assess mortality risks and tailor interventions. Importantly, the type prevalences presented are determined by our outcome definitions. For example, we defined SGA as birthweight less than the 10th centile of a normal healthy population, whereas less than the 3rd centile may better reflect the frequency of FGR and associated mortality risks. Many studies utilised LMP for gestational age or had missing birthweights that could bias estimates of type prevalence. We did not evaluate the potential influence of trial interventions on type prevalence, given the diversity of study designs and interventions among included datasets. Future studies could undertake sensitivity analyses to explore associations between newborn types and trial intervention assignment, among other factors, such as multiple births, and consider mortality risks for newborn types and by detailed strata of gestational age, birthweight and weight for gestational age. Our analysis included only live births, excluding stillbirths, which is the focus of another paper in this series.

We did not present global or regional estimates of newborn types, as the included studies are not sufficiently representative of the heterogeneous populations in each region and over the study period. Instead, the Vulnerable Newborn Measurement Collaboration team will produce global and regional newborn type prevalence estimates using a Bayesian modelling framework and comprehensive set of data inputs from many countries worldwide, including population‐level data on PT and LBW and individual‐level data from national health information systems and the population‐based studies described in this paper.^14^, ^17^

## 5 CONCLUSION

Our study is the first multi‐country analysis to present detailed newborn type prevalences for a large number of LMICs. LBW is a common marker of vulnerability for newborns, and previous estimates of LBW, PT and SGA and their health consequences have been generated independently.^9^, ^27^, ^32^ This division leads to a siloed approach to policy and programming, despite many overlapping biological pathways, epidemiological risk factors, and interventions to improve health outcomes among small vulnerable newborns and reduce risk of neonatal mortality. Considering combinations of types also enables description of the prevalence of and risk associated with LGA, which although not as common as being small at birth, has been neglected. There may be benefits to a classification of six or four newborn types, using PT/T and AGA/SGA and possibly LGA, while excluding LBW/nonLBW, including alignment of aetiologies, risk factors, and mortality and morbidity risks more clearly with specific birth outcomes as well as opportunities to evaluate targeted preventive and therapeutic interventions to reduce adverse health outcomes and promote healthy growth and development. Further investigation is needed to describe the mortality and morbidity risks associated with specific newborn types in pursuit of a framework to support local evaluation and targeting of interventions to prevent adverse pregnancy outcomes in LMICs.^35^, ^36^

## AUTHOR CONTRIBUTIONS

The Vulnerable Newborn Measurement Collaboration was planned by JEL and REB. This analysis was designed by DE, EH, JK and ACL with REB. All authors contributed to the study protocol, with inputs from the wider Vulnerable Newborn Measurement Collaboration. Analysis was undertaken by DE, EH and MD. The manuscript was drafted by DE with EH, JK, ACL, MD and REB. All authors helped revise the paper. All authors reviewed and agreed on the final version.

## FUNDING INFORMATION

The Children's Investment Fund Foundation, grant 2004‐04670. The funders had no role in the study design, data collection, analysis or interpretation of the paper.

## CONFLICT OF INTEREST STATEMENT

See Appendices S1–S8.

## ETHICS APPROVAL

The Vulnerable Newborn Measurement Collaboration was granted ethical approval from the Institutional Review Boards of the London School of Hygiene & Tropical Medicine (ref: 22858) and Johns Hopkins University (see the Appendices S1–S8). All collaborators received local ethical permission for their data where relevant.

## Supporting information


Appendix S1–S8.

## Data Availability

Data sharing and transfer agreements were jointly developed and signed by all collaborating partners. The pooled summary table data generated during the current study are deposited online with data access subject to approval at https://doi.org/10.17037/DATA.00003095.

## APPENDIX 1

### Subnational Collaborative Group for Vulnerable Newborn Prevalence*

Argentina (2000): None. Bangladesh (2001, 2007): Hasmot Ali, Parul Christian, Rolf D. W. Klemm, Alan B. Massie, Maithili Mitra, Sucheta Mehra, Kerry J. Schulze, Abu Ahmed Shamim, Alfred Sommer, MD. Barkat Ullah, Keith P. West, Jr. Bangladesh (2004, 2011): Nazma Begum, Nabidul Haque Chowdhury, Md. Shafiqul Islam, Dipak Kumar Mitra, Abdul Quaiyum. Bangladesh (2014): None. Botswana (2014): Modiegi Diseko, Joseph Makhema. Brazil (2015): None. Burkina Faso (2004): None. Burkina Faso (2006): None. China (2002): Yue Cheng. China (2012): Yixin Guo, Shanshan Yuan. Ethiopia (2017): Meselech Roro, Bilal Shikur. Ethiopia (2018): Frederick Goddard, Sebastien Haneuse, Bezawit Hunegnaw. Ethiopia (2020): Yemane Berhane, Alemayehu Worku. Ghana (2008): Seyram Kaali. Ghana (2009): Charles D. Arnold. Ghana (2013): Darby Jack. Ghana (2014): Seeba Amenga‐Etego, Lisa Hurt, Caitlin Shannon, Seyi Soremekun. Guatemala (2013): None. India (2000): None. India (2010): Nita Bhandari, Jose Martines, Sarmila Mazumder. India (2013): None. India (2016): Yamuna Ana, Deepa R. Malawi (2003): Lotta Hallamaa, Juha Pyykkö. Malawi (2011): None. Mexico (2017): Mario I. Lumbreras‐Marquez, Claudia E. Mendoza‐Carrera. Nepal (2002): None. Nepal (2010): None. Pakistan (2013): None. Pakistan (2014): Atiya Hussain, Muhammad Karim, Farzana Kausar, Usma Mehmood, Naila Nadeem, Muhammad Imran Nisar, Muhammad Sajid. Papua New Guinea (2009): Ivo Mueller, Maria Ome‐Kaius. Rwanda (2017): Elizabeth Butrick, Felix Sayinzoga. South Africa (2016): None. Sri Lanka (2015): Ilaria Mariani. Tanzania (2001): Willy Urassa. Tanzania (2008): Thor Theander, Phillippe Deloron, Birgitte Bruun Nielsen. Tanzania (2010): Alfa Muhihi, Ramadhani Abdallah Noor. Tanzania (2014a): Ib Bygbjerg, Sofie Lykke Moeller. Tanzania (2014b): Fahad Aftab, Said M. Ali, Pratibha Dhingra, Usha Dhingra, Arup Dutta, Sunil Sazawal, Atifa Suleiman, Mohammed Mohammed, Saikat Deb. Thailand (2000): None. Uganda (2016): Moses R. Kamya, Miriam Nakalembe. Uganda (2018): Jude Mulowooz, Nicole Santos. Zambia (2011): Godfrey Biemba, Julie M. Herlihy, Reuben K. Mbewe, Fern Mweena, Kojo Yeboah‐Antwi. Zambia (2013): Jane Bruce, Daniel Chandramohan. Zambia (2014): None. Zambia (2015): None. Zimbabwe (2012): Andrew Prendergast. *Individuals involved in multiple studies on this list are only named once.

### Vulnerable Newborn Measurement Core Group

LSHTM: Joy E. Lawn, Hannah Blencowe, Eric Ohuma, Yemi Okwaraji, Judith Yargawa, Ellen Bradley. JHU: Robert E. Black, Joanne Katz, Daniel J. Erchick, Elizabeth Hazel, Michael Diaz, Anne C. C. Lee.

## References

[1] United Nations Interagency Group for Child Mortality Estimation (UN IGME) . Levels and trends in child mortality. New York: United Nations Interagency Group for Child Mortality Estimation; 2021.

[2] World Health Organization . Every newborn: an action plan to end preventable deaths. Geneva: World Health Organization; 2014.

[3] Lawn JE , Blencowe H , Oza S , You D , Lee AC , Waiswa P , et al. Every newborn: progress, priorities, and potential beyond survival. Lancet. 2014;384(9938):189–205. 24853593 10.1016/S0140-6736(14)60496-7

[4] UN General Assembly . Transforming our world: the 2030 agenda for sustainable development. 2015. [cited 2015 Jul 27]. Available from: https://sustainabledevelopment.un.org/post2015/transformingourworld

[5] Perin J , Mulick A , Yeung D , Villavicencio F , Lopez G , Strong KL , et al. Global, regional, and national causes of under‐5 mortality in 2000‐19: an updated systematic analysis with implications for the sustainable development goals. Lancet Child Adolesc Health. 2022;6(2):106–15. 34800370 10.1016/S2352-4642(21)00311-4 PMC8786667

[6] Christian P , Lee SE , Donahue Angel M , Adair LS , Arifeen SE , Ashorn P , et al. Risk of childhood undernutrition related to small‐for‐gestational age and preterm birth in low‐ and middle‐income countries. Int J Epidemiol. 2013;42(5):1340–55. 23920141 10.1093/ije/dyt109 PMC3816349

[7] Fall CH . Fetal malnutrition and long‐term outcomes. Nestle Nutr Inst Workshop Ser. 2013;74:11–25. 23887100 10.1159/000348384 PMC5081104

[8] World Health Organization/Unicef . The extension of the 2025 maternal, infant and young child nutrition targets to 2030. 2019.

[9] Blencowe H , Krasevec J , de Onis M , Black RE , An X , Stevens GA , et al. National, regional, and worldwide estimates of low birthweight in 2015, with trends from 2000: a systematic analysis. Lancet Glob Health. 2019;7(7):e849–e60. 31103470 10.1016/S2214-109X(18)30565-5 PMC6560046

[10] Lee AC , Katz J , Blencowe H , Cousens S , Kozuki N , Vogel JP , et al. National and regional estimates of term and preterm babies born small for gestational age in 138 low‐income and middle‐income countries in 2010. Lancet Glob Health. 2013;1(1):e26–36. 25103583 10.1016/S2214-109X(13)70006-8 PMC4221634

[11] Katz J , Lee AC , Kozuki N , Lawn JE , Cousens S , Blencowe H , et al. Mortality risk in preterm and small‐for‐gestational‐age infants in low‐income and middle‐income countries: a pooled country analysis. Lancet. 2013;382(9890):417–25. 23746775 10.1016/S0140-6736(13)60993-9 PMC3796350

[12] Hughes MM , Black RE , Katz J . 2500‐g low birth weight cutoff: history and implications for future research and policy. Matern Child Health J. 2017;21(2):283–9. 27449779 10.1007/s10995-016-2131-9 PMC5290050

[13] Mendez‐Figueroa H , Truong VTT , Pedroza C , Chauhan SP . Large for gestational age infants and adverse outcomes among uncomplicated pregnancies at term. Am J Perinatol. 2017;34(7):655–62. 27926975 10.1055/s-0036-1597325

[14] Ashorn P , Black RE , Lawn JE , Ashorn U , Klein N , Hofmeyr J , et al. The Lancet small vulnerable newborn series: science for a healthy start. Lancet. 2020;396(10253):743–5. 32919498 10.1016/S0140-6736(20)31906-1

[15] Lawn JE , Ohuma EO , Bradley E , Idueta LS , Hazel EA , Okwaraji YB . Small babies, big risks: global estimates of prevalence and mortality for vulnerable newborns to accelerate change and improve counting. Lancet. 2023. in press. 10.1016/S0140-6736(23)00522-6 37167989

[16] Villar J , Cheikh Ismail L , Victora CG , Ohuma EO , Bertino E , Altman DG , et al. International standards for newborn weight, length, and head circumference by gestational age and sex: the newborn cross‐sectional study of the INTERGROWTH‐21st project. Lancet. 2014;384(9946):857–68. 25209487 10.1016/S0140-6736(14)60932-6

[17] Suárez‐Idueta L . Vulnerable newborn types: analysis of population‐based registries for 165 million births in 23 countries, 2000 to 2021. BJOG. 2022. 10.1111/1471-0528.17505 PMC12678069 37156241

[18] United Nations Children’s Fund and World Health Organization . Low birthweight: Country regional and global estimates. New York: UNICEF; 2014.

[19] Kong S , Day LT , Zaman SB , Peven K , Salim N , Sunny AK , et al. Birthweight: EN‐BIRTH multi‐country validation study. BMC Pregnancy Childbirth. 2021;21(1):240. 33765936 10.1186/s12884-020-03355-3 PMC7995711

[20] Mikkelsen L , Phillips DE , AbouZahr C , Setel PW , de Savigny D , Lozano R , et al. A global assessment of civil registration and vital statistics systems: monitoring data quality and progress. Lancet. 2015;386(10001):1395–406. 25971218 10.1016/S0140-6736(15)60171-4

[21] United Nations Statistics Division . Quality of vital statistics obtained from civil registration. New York, NY: United Nations Statistics Division; 2021.

[22] Lazzerini M , Senanayake H , Mohamed R , Kaluarachchi A , Fernando R , Sakalasuriya A , et al. Implementation of an individual patient prospective database of hospital births in Sri Lanka and its use for improving quality of care. BMJ Open. 2019;9(2):e023706. 10.1136/bmjopen-2018-023706 PMC6368149 30782885

[23] Hambidge KM , Krebs NF , Westcott JE , Garces A , Goudar SS , Kodkany BS , et al. Preconception maternal nutrition: a multi‐site randomized controlled trial. BMC Pregnancy Childbirth. 2014;14:111. 24650219 10.1186/1471-2393-14-111 PMC4000057

[24] Sharma V , Katz J , Mullany LC , Khatry SK , LeClerq SC , Shrestha SR , et al. Young maternal age and the risk of neonatal mortality in rural Nepal. Arch Pediatr Adolesc Med. 2008;162(9):828–35. 18762599 10.1001/archpedi.162.9.828 PMC2535853

[25] Katz J , West KP Jr , Khatry SK , Christian P , LeClerq SC , Pradhan EK , et al. Risk factors for early infant mortality in Sarlahi district, Nepal. Bull World Health Organ. 2003;81(10):717–25. 14758431 PMC2572324

[26] World Health Organization . Increasing access to health workers in remote and rural areas through improved retention. Global policy recommendations. Geneva: World Health Organization; 2010. 23741785

[27] Lee AC , Kozuki N , Cousens S , Stevens GA , Blencowe H , Silveira MF , et al. Estimates of burden and consequences of infants born small for gestational age in low and middle income countries with INTERGROWTH‐21(st) standard: analysis of CHERG datasets. BMJ. 2017;358:j3677. 28819030 10.1136/bmj.j3677 PMC5558898

[28] Gurung S , Tong HH , Bryce E , Katz J , Lee AC , Black RE , et al. A systematic review on estimating population attributable fraction for risk factors for small‐for‐gestational‐age births in 81 low‐ and middle‐income countries. J Glob Health. 2022;12:04024. 35356650 10.7189/jogh.12.04024 PMC8942297

[29] Hasan MM , Magalhaes RJS , Fatima Y , Ahmed S , Mamun AA . Levels, trends, and inequalities in using institutional delivery services in low‐ and middle‐income countries: a stratified analysis by facility type. Glob Health Sci Pract. 2021;9(1):78–88. 33795363 10.9745/GHSP-D-20-00533 PMC8087431

[30] Helleringer S , Liu L , Chu Y , Rodrigues A , Fisker AB . Biases in survey estimates of neonatal mortality: results from a validation study in urban areas of Guinea‐Bissau. Demography. 2020;57(5):1705–26. 32914335 10.1007/s13524-020-00911-6

[31] Saya AR , Katz J , Khatry SK , Tielsch JM , LeClerq SC , Mullany LC . Causes of neonatal mortality using verbal autopsies in rural southern Nepal, 2010–2017. PLOS Glob Public Health. 2022;2(9):e0001072. 36962665 10.1371/journal.pgph.0001072 PMC10021801

[32] Chawanpaiboon S , Vogel JP , Moller AB , Lumbiganon P , Petzold M , Hogan D , et al. Global, regional, and national estimates of levels of preterm birth in 2014: a systematic review and modelling analysis. Lancet Glob Health. 2019;7(1):e37–46. 30389451 10.1016/S2214-109X(18)30451-0 PMC6293055

[33] Vwalika B , Price JT , Rosenbaum A , Stringer JSA . Reducing the global burden of preterm births. Lancet Glob Health. 2019;7(4):e415. 30879504 10.1016/S2214-109X(19)30060-9

[34] Karl S , Li Wai Suen CSN , Unger HW , Ome‐Kaius M , Mola G , White L , et al. Preterm or not – an evaluation of estimates of gestational age in a cohort of women from rural Papua New Guinea. PLoS One. 2015;10(5):e0124286. 25945927 10.1371/journal.pone.0124286 PMC4422681

[35] Hewish A , Dibley M , Huda T . The neonatal mortality risk of different types of vulnerable newborns in rural Bangladesh: a prospective cohort study within the Shonjibon trial. Curr Dev Nutr. 2022;6(Suppl 1):663. 10.1111/tmi.14092 PMC11965014 39894679

[36] Suárez‐Idueta L . Neonatal mortality risk for term large‐for‐gestational age and macrosomic livebirths in 15 countries using 115.6 million nationwide linked records, 2000 to 2020. BJOG. in press. 10.1111/1471-0528.17706 PMC12678065 38012114

