# Supplementary material for: Vulnerable newborn types: analysis of subnational, population‐based birth cohorts for 541 285 live births in 23 countries, 2000–2021
Source: BJOG. 2023 May 8;132(Suppl 8):S20–36. doi: 10.1111/1471-0528.17510 (PMC12678066; doi:10.1111/1471-0528.17510)
Supplement: Supplementary file 1 — Appendix S1–S8. [file BJO-132-S20-s001.docx]

# **SUPPLEMENT TITLE**

Vulnerable newborn multi-country analyses related to preterm births or small-for-gestational-age

# **PAPER TITLE**

Vulnerable newborn types: analysis of subnational, population-based birth cohorts for 541,285 live births in 23 countries, 2000 to 2021

# **RUNNING TITLE**

Vulnerable newborn types from subnational birth cohorts in 23 countries

**SUPPORTING INFORMATION**

**Appendix S1: STROBE checklist**

|  | Item No | Recommendation | Page  No. |
| --- | --- | --- | --- |
| **Title and abstract** | 1 | (*a*) Indicate the study’s design with a commonly used term in the title or the abstract | 1-6 |
|  |  | (*b*) Provide in the abstract an informative and balanced summary of what was done and what was found | 1-6 |
| Introduction | | |  |
| Background/rationale | 2 | Explain the scientific background and rationale for the investigation being reported | 7-8 |
| Objectives | 3 | State specific objectives, including any prespecified hypotheses | 8 |
| Methods | | |  |
| Study design | 4 | Present key elements of study design early in the paper | 9 |
| Setting | 5 | Describe the setting, locations, and relevant dates, including periods of recruitment, exposure, follow-up, and data collection | 9 |
| Participants | 6 | (*a*) *Cohort study*—Give the eligibility criteria, and the sources and methods of selection of participants. Describe methods of follow-up  *Case-control study*—Give the eligibility criteria, and the sources and methods of case ascertainment and control selection. Give the rationale for the choice of cases and controls  *Cross-sectional study*—Give the eligibility criteria, and the sources and methods of selection of participants | 9-10 |
|  |  | (*b*) *Cohort study*—For matched studies, give matching criteria and number of exposed and unexposed  *Case-control study*—For matched studies, give matching criteria and the number of controls per case |  |
| Variables | 7 | Clearly define all outcomes, exposures, predictors, potential confounders, and effect modifiers. Give diagnostic criteria, if applicable | 9-10 |
| Data sources/ measurement | 8* | For each variable of interest, give sources of data and details of methods of assessment (measurement). Describe comparability of assessment methods if there is more than one group | 9-10 |
| Bias | 9 | Describe any efforts to address potential sources of bias | 9-10 |
| Study size | 10 | Explain how the study size was arrived at | 9 |
| Quantitative variables | 11 | Explain how quantitative variables were handled in the analyses. If applicable, describe which groupings were chosen and why | 9-10 |
| Statistical methods | 12 | (*a*) Describe all statistical methods, including those used to control for confounding | 10 |
|  |  | (*b*) Describe any methods used to examine subgroups and interactions | 10 |
|  |  | (*c*) Explain how missing data were addressed | 10 |
|  |  | (*d*) *Cohort study*—If applicable, explain how loss to follow-up was addressed  *Case-control study*—If applicable, explain how matching of cases and controls was addressed  *Cross-sectional study*—If applicable, describe analytical methods taking account of sampling strategy | 10 |
|  |  | (*e*) Describe any sensitivity analyses | 10 |

| Results | | | Page  No. |
| --- | --- | --- | --- |
| Participants | 13* | (a) Report numbers of individuals at each stage of study—eg numbers potentially eligible, examined for eligibility, confirmed eligible, included in the study, completing follow-up, and analysed | 11 |
|  |  | (b) Give reasons for non-participation at each stage | 11,21 |
|  |  | (c) Consider use of a flow diagram | 21 |
| Descriptive data | 14* | (a) Give characteristics of study participants (eg demographic, clinical, social) and information on exposures and potential confounders | 11-12 |
|  |  | (b) Indicate number of participants with missing data for each variable of interest | 11 |
|  |  | (c) *Cohort study*—Summarise follow-up time (eg, average and total amount) | 11 |
| Outcome data | 15* | *Cohort study*—Report numbers of outcome events or summary measures over time | 11-12 |
|  |  | *Case-control study—*Report numbers in each exposure category, or summary measures of exposure |  |
|  |  | *Cross-sectional study—*Report numbers of outcome events or summary measures |  |
| Main results | 16 | (*a*) Give unadjusted estimates and, if applicable, confounder-adjusted estimates and their precision (eg, 95% confidence interval). Make clear which confounders were adjusted for and why they were included | 11-12 |
|  |  | (*b*) Report category boundaries when continuous variables were categorized |  |
|  |  | (*c*) If relevant, consider translating estimates of relative risk into absolute risk for a meaningful time period |  |
| Other analyses | 17 | Report other analyses done—eg analyses of subgroups and interactions, and sensitivity analyses | 11-12 |
| Discussion | | |  |
| Key results | 18 | Summarise key results with reference to study objectives | 13 |
| Limitations | 19 | Discuss limitations of the study, taking into account sources of potential bias or imprecision. Discuss both direction and magnitude of any potential bias | 15 |
| Interpretation | 20 | Give a cautious overall interpretation of results considering objectives, limitations, multiplicity of analyses, results from similar studies, and other relevant evidence | 13-15 |
| Generalisability | 21 | Discuss the generalisability (external validity) of the study results | 13-15 |
| Other information | | |  |
| Funding | 22 | Give the source of funding and the role of the funders for the present study and, if applicable, for the original study on which the present article is based | 18 |

*Give information separately for cases and controls in case-control studies and, if applicable, for exposed and unexposed groups in cohort and cross-sectional studies.

**Note:** An Explanation and Elaboration article discusses each checklist item and gives methodological background and published examples of transparent reporting. The STROBE checklist is best used in conjunction with this article (freely available on the Web sites of PLoS Medicine at http://www.plosmedicine.org/, Annals of Internal Medicine at http://www.annals.org/, and Epidemiology at http://www.epidem.com/). Information on the STROBE Initiative is available at www.strobe-statement.org.

**Appendix S2: Ethics approval or exemptions of Institutional Review Boards**

| **Study name** | **Institutional Review Board(s) or data access provider** | **Ref/Number*** | **Date of approval** |
| --- | --- | --- | --- |
| Argentina (2000) | Data retrieved from long-standing anonymized database through data access agreement with the *Miguel Larguía Foundation* | N/A | N/A |
| Bangladesh (2001) | JiVitA-1 | IRB000011017 | 19 Nov 2021 |
| Bangladesh (2007) | JiVitA-3 | IRB00000570 | 30 Nov 2021 |
| Bangladesh (2011) | Johns Hopkins Bloomberg School of Public Health Institutional Review Board  Icddr,b/ERC, Bangladesh | 3151  pr11-017 | 2011 |
| Bangladesh (2014) | Johns Hopkins Bloomberg School of Public Health Institutional Review Board  Icddr,b/ERC, Bangladesh | IRB00004508  PR12-073 | 2012 |
| Botswana (2014) | Harvard TH Chan School of Public Health, Institutional Review Board  Health Research Development Committee, Botswana | Unavailable. | Unavailable. |
| Brazil (2015) | School of Physical Education Ethics Committee at the Federal University of Pelotas | CAAE registration number: 26746414.5.0000.5313 | 5 February 2014 |
| Burkina Faso (2004) | Ethics committee of Centre Muraz, Bobo-Dioulasso, Burkina Faso  Ethics committee of the Institute of Tropical Medicine, Antwerp, Belgium | Unavailable. | Unavailable. |
| Burkina Faso (2006) | Ethics committee of Centre Muraz, Bobo-Dioulasso, Burkina Faso  Ethics committee of the Institute of Tropical Medicine, Antwerp, Belgium | Unavailable. | 2001 |
| China (2002) | The Human Research Ethics Committee of the College of Medicine, Xi’an Jiaotong University | No 2002001 | 10 April 2002 |
| China (2012) | the Institutional Ethics Committee of Guangzhou Women and Children’s Medical Center | 2017102302 | 12 November 2017 |
| Ethiopia (2017) | Addis Ababa University, Ethiopia | 099/17/SPH | 8 March 2018 |
| Ethiopia (2018) | St. Paul’s Hospital Millennium Medical college  Boston Children Hospital | PM23/274  IRB-P00028224 | 29 March 2018 |
| Ethiopia (2020) | Addis Continental Institute of Public Health, Institutional Ethical Review Board (IRB/ACIPH)  Partners Health Care/Mass General Brigham | IRB registration/identification number 0029 (Ministry of Science and Technology, Ethiopia )  2018P002479  0029 MSTEthiopia is the granting authority for ACIPH to provide IRB reviews.  ENAT IRB is:  ACIPH/IRB/001-A3/2021 | 11 January 2019    26 February 2019 |
| Ghana (2009) | Ghana Health Services Ethics Review Committee (GHS-ERC)  University of Ghana Noguchi Memorial Institute for Medical Research (NMIMR)  UC Davis Institutional Review Board | GHS-ERC-05/07/09  042/08-09 revd. 2017  223623-6 (formerly #200917276) | 8 July 2009  17 June 2009  25 June 2009 |
| Ghana (2013) | Kintampo Health Research Centre Institutional Ethics Committee | 2011/02 | 13 November 2012 |
| Guatemala (2013) | The project was approved by the Colorado Multiple Institutional Review Board, University of Colorado, the local or/and national ethics committees for each of the three research sites: Guatemala—Comite de Etica Universidad Francisco Marroquin; India—Institutional Ethics Committee on Human Subjects Research, KLE Society’s JNMC Institutional Ethics Committee on Human Subjects Research; Pakistan—Aga Khan University Ethical Review Committee. | Guatemala: 034-14  India: MDC/IECHSR/2013-14/A25  Pakistan: 2753-CHS-ERC-13 | Unavailable. |
| India (2000) | Aravind Eye and Children’s Hospitals, the Department of Health, Tamil Nadu State Government, and the Committee on Human Research of the Johns Hopkins Bloomberg School of Public Health | IRB No: H.22.02.03.06.C1 | Unknown |
| India (2010) | Ethics Review Committee, Society for Applied Studies, 45, Kalu Sarai, New Delhi-110016 | Protocol version 1.0 | November 18,2009 |
| India (2013) | The project was approved by the Colorado Multiple Institutional Review Board, University of Colorado, the local or/and national ethics committees for each of the three research sites: Guatemala—Comite de Etica Universidad Francisco Marroquin; India—Institutional Ethics Committee on Human Subjects Research, KLE Society’s JNMC Institutional Ethics Committee on Human Subjects Research; Pakistan—Aga Khan University Ethical Review Committee. | Guatemala: 034-14  India: MDC/IECHSR/2013-14/A25  Pakistan: 2753-CHS-ERC-13 | Unavailable. |
| India (2016) | Institutional ethical review board, Indian Institute of Public Health-H, Bengaluru. | (Ref No: IIPHHB/TRCIEC/091/2015 | 13/11/2015 |
| Malawi (2003) | COMREC, College of Medicine Research and Ethics Committee, University of Malawi | P.01/02/169 | April 30, 2003 |
| Malawi (2011) | COMREC, College of Medicine Research and Ethics Committee, University of Malawi | P.08/10/972 | November 17, 2010 |
| Mexico (2017) | Unavailable. | Unavailable. | Unavailable. |
| Nepal (2002) | Committee on Human Research of the Johns Hopkins Bloomberg School of Public Health  Nepal Health Research Council | H.22.01.09.25.A1 | 2001 |
| Nepal (2010) | Johns Hopkins Bloomberg School of Public Health Institutional Review Board  Institute of Medicine, Tribhuvan University | IRB00001735  #217 (6-11-E) | 2009  2009 |
| Pakistan (2013) | The project was approved by the Colorado Multiple Institutional Review Board, University of Colorado, the local or/and national ethics committees for each of the three research sites: Guatemala—Comite de Etica Universidad Francisco Marroquin; India—Institutional Ethics Committee on Human Subjects Research, KLE Society’s JNMC Institutional Ethics Committee on Human Subjects Research; Pakistan—Aga Khan University Ethical Review Committee. | Guatemala: 034-14  India: MDC/IECHSR/2013-14/A25  Pakistan: 2753-CHS-ERC-13 | Unavailable. |
| Pakistan (2014) | WHO ERC | RPC 532 | 18.01.2021 (continuing review for extension of AMANHI study) |
| Papua New Guinea (2009) | Papua New Guinea Institute of Medical Research Institutional Review Board  Medical Research Advisory Council of Papua New Guinea  Melbourne Health Human Research Ethics Committee | 08.15  08.01   2008.162 | 23 Feb 2008  23 Feb 2008  24 Sep 2008 |
| Rwanda (2017) | UCSF Human Research Protection Program IRB  Rwanda National Ethics Committee | 16-21177  No.0034/RNEC/2017 | 1 Mar 2017  20 Feb 2017 |
| South Africa (2016) | South African Medical Research Council IRB | Protocol ID: EC036-11/2015 | 23 November 2015 |
| Sri Lanka (2015) | The study was approved by the Ethics Review Committee of the Faculty of Medicine, University of Colombo, Sri Lanka | EC-15–017/2015 | 2015 |
| Tanzania (2001) | The study was approved by the institutional review boards at Muhimbili University of Health and Allied Sciences in Dar es Salaam, Tanzania (NIMR/HQ/R.8a/Vol. IX/2649)  Harvard T.H. Chan School of Public Health in Boston, USA (IRB-10433). | Tanzania: NIMR/HQ/R.8a/Vol. IX/2649  Harvard: IRB-10433 | Unavailable. |
| Tanzania (2008) | Medical Research Coordinating Committee (MRCC), housed by National Institute for Medical Research, Tanzania | NIMR/HQ/R.8a/Vol.IX/688 | 18 April 2008 |
| Tanzania (2010) | Harvard School of Public Health    Ifakara Health Institute    National Medical Research Coordinating Council of Tanzania (NIMR)  WHO Ethical Review Committee | Harvard: OHRA Protocol # 18195  Ifakara: IHI/IRB/No. A77- 2009  NIMR: NIMR/HQ/R.8a/Vol.  WHO: RPC356 | October 12, 2009  2009  2009  2009 |
| Tanzania (2014a) | Medical Research Coordinating Committee (MRCC), housed by National Institute for Medical Research, Tanzania | NIMR/HQ/R.8a/Vol. IX/1717 | 6 May 2014 |
| Tanzania (2014b) | WHO ERC | RPC 532 | 18.01.2021 (continuing review for extension of AMANHI study) |
| Thailand (2000) | EC submission approval, the Ministry of Thailand Public Health EC board | 40/2546 | 22 September 2000 |
| Uganda (2016) | Makerere University School of Biomedical Sciences Research Ethics Committee  University of California San Francisco | SBS 342  16-18679 | 13 May 2016  22 April 2016 |
| Uganda (2018) | UCSF Human Research Protection Program IRB  School of Public Heath, Makerere University Higher Degrees Research and Ethics Committee | 17-22310  515 | 28 Oct 2017  11 Nov 2017 |
| Zambia (2011) | The Boston University Medical Campus Institutional Review Board (FWA00000301)  University of Zambia Research Ethics Committee (FWA00000338) provided ethical approval, and the Zambian Ministry of Health approved the study to be undertaken in Zambia. | BU IRB: H-29647  UNZA REC: 001-01-10 | Unavailable. |
| Zambia (2013) | Observational / Interventions Research Ethics Committee  University of Zambia Biomedical Research Ethics Committee | 6292  004-02-13 | 10 December 2012  13 September 2013 |
| Zambia (2014) | WHO ERC | RPC 532 | 18.01.2021 (continuing review for extension of AMANHI study) |
| Zambia (2015) | University of Zambia Biomedical Research Ethics Committee (UNZA-BREC)  University of Alabama at Birmingham (UAB IRB) | 012-12-16  300000669 | January 17, 2017 |
| Zimbabwe (2012) | Medical Research Council of Zimbabwe  International Review Board of the Johns Hopkins Bloomberg School of Public Health | IRB # MRCZ-A-1675  IRB # 00004205 | 27 October 2012  02 April 2012 |

* Institutional Review Board reference numbers and approval dates are marked ‘unavailable’ for studies whose authors could no longer find these details for their studies.

**Appendix S3: Definitions**

| **Definitions** |  |
| --- | --- |
| Live birth | Is the complete expulsion or extraction from a woman of a fetus, irrespective of the duration of the pregnancy, which, after such separation, shows signs of life. |
| Birthweight | Is defined as the weight of the fetus or newborn obtained immediately after birth. For livebirths, measurement of birthweight within the first hour of life before significant postnatal weight loss has occurred is preferable. If the birth weight was measured repeatedly, the median value will be used. Birthweight can be measured using digital or analogue scales. For the purposes of this work, weights of newborns taken at ≥72 hours after birth will be excluded. |
| Gestational age | The duration of gestation measured from the first day of the last menstrual period (LMP). Gestational age will be analyzed in days where possible. Gestational age measured by LMP, early pregnancy ultrasound or best obstetric estimate (BEO) will be included. |
| Neonatal death | A neonatal death is defined as a death during the first 28 days after live birth (days 0-27). An early neonatal death is a death during the first 7 days after live birth (days 0 – 6), a late neonatal death is a death day 7 – 27 after a livebirth. |
| Stillbirth | A stillbirth is ‘the complete expulsion or extraction from a woman of a fetus following a fetal death at 22 or more completed weeks of gestation; or if gestational age is not available with a birthweight of 500 grams or more. |
| **Calculated variables** |  |
| Preterm birth | A birth before 37 completed weeks of gestation (or before 259 days of gestation) as measured from the first day of the last menstrual period (LMP) or by early ultrasound. |
| Term birth | A birth from 37 completed weeks of gestation as measured from the first day of the last menstrual period (LMP) or by early ultrasound. |
| Low birthweight | A birth with birthweight of less than 2,500 grams. |
| Non-low birthweight | A birth with birthweight of ≥2,500 grams. |
| Small for gestational age | A birth with a birthweight for gestational age and sex of <10th centile according to INTERGROWTH-21st international standards. |
| Appropriate for gestational age | A birth with a birthweight for gestational age and sex from 10th to 90th centiles according to INTERGROWTH-21st international standards. |
| Large for gestational age | A birth with a birthweight for gestational age and sex of >90th centile according to INTERGROWTH-21st international standards. |

# **Appendix S4: Illustration of four newborn types based on birthweight, gestational age, and size for gestational age**


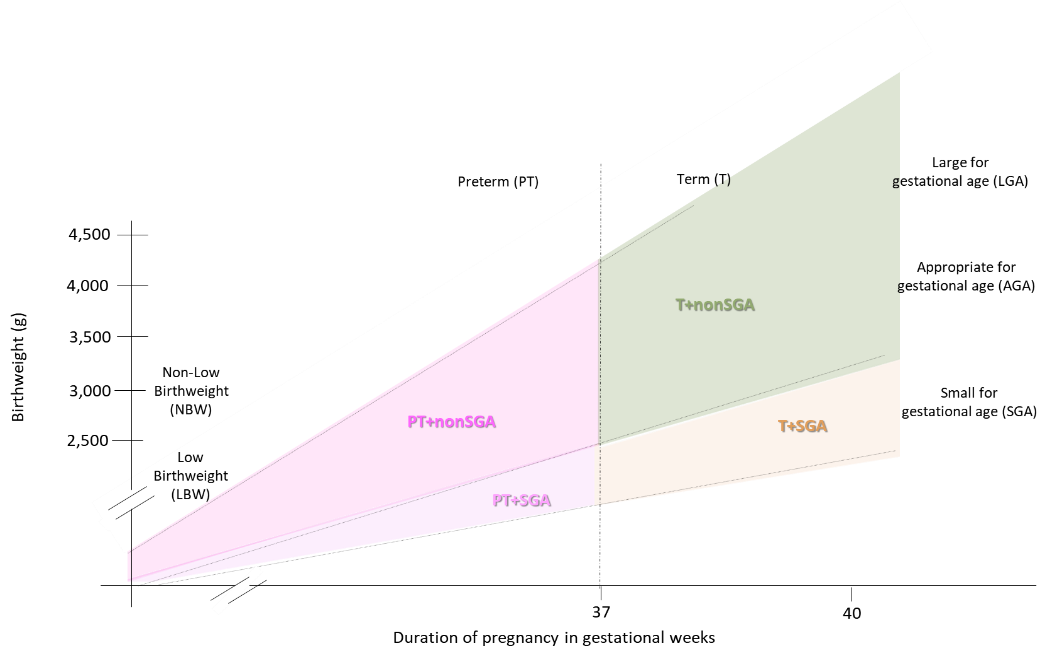


Original newborn types proposed by Ashorn et al.

**Appendix S5: Identification of subnational, prospective birth cohort studies for inclusion in this analysis**

Study inclusion criteria

- **Sample size:** Data must be from large studies (sample size of at least 300) involving birth cohorts, with high-quality gestational age, sex, and birthweight data.
- **Study population:**
  - Data can be from population-based studies or hospital-based studies in settings where approximately 80% of birth occur in hospitals.
  - Recruitment from ANC clinics is eligible if approximately 90% of women received at least one ANC visit in the study area.
- **Timing:** Data collection began in year 2000 or later.
- **Outcome assessment:**
  - Gestational age assessment conducted by ultrasound (1st or 2nd trimester) or my last menstrual period method.
  - Birthweight measured by digital scales (other methods acceptable) within <72 hours of birth for a high proportion of babies.
- **Data missingness:**
  - Gestational age has missingness of less than 20% of live births.
  - Birthweight data should have missingness of less than 20% of live births.

Literature review methodology

**Article databases:**

- **PubMed, Embase, Scopus, OVID Global Health**
- **Clinical trial registries and pre-print**
  - ClinicalTrials.gov
  - ISRCTN - <https://www.isrctn.com/>
  - Open Trials - <https://opentrials.net/>
  - medRxiv - <https://www.medrxiv.org/>

**Search inclusion/exclusion criteria:**

- Article published in any language
- Articles published since 2000
- Study conducted in a low- or middle-income country
- Study design inclusion: prospective cohort studies, randomized trials
- Study design exclusion: systematic reviews, meta-analyses, book chapters

**Search terms:**

(“small for gestational age” OR “intrauterine growth restriction”) AND (“low birthweight” OR “low birth weight”) AND (prematurity OR “premature labor”)

**Search strategy:**

- Review title/abstracts of relevant papers by single reviewer
- Full-text review of selected papers with inclusion criteria
  - Data must be from (1) nationally representative datasets with cross-linked birth data, in particular gestational age, sex, and birthweight, or (2) large studies (sample size of at least 300) involving birth cohorts, with high-quality gestational age, sex, and birthweight data.
    - - Data can be from population-based studies or from hospital-based studies in settings where at least 80% of births occur in hospitals.
      - For national datasets, we expect a high national coverage of at least 80% of estimated births per year.
  - Data collection began in year 2000 or later.
  - Gestational age assessment conducted by ultrasound (1^st^ or 2^nd^ trimester) (although other methods acceptable)
  - Birthweights measured by digital scales (other methods acceptable) within <72 hours of birth for a high proportion of babies.
  - Gestational age and birthweight data combined should have missingness of less than 20%.
- If study meets the criteria, contact the authors with the data request.

**Appendix S6: Additional analyses and results**

S6a: Flowchart of database construction for all studies


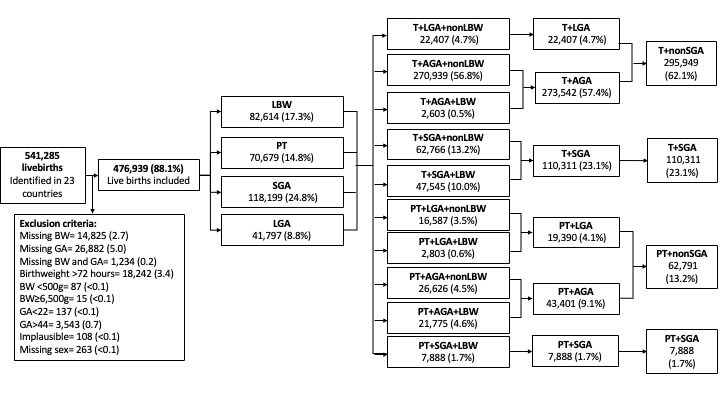


This figure depicts how the pooled dataset was derived to calculate the prevalence of birth outcomes and vulnerable newborn types.

S6b: Median and interquartile range for PT, LBW, SGA, and LGA outcomes among 476,939 live births included from 45 studies by SDG Regions

**
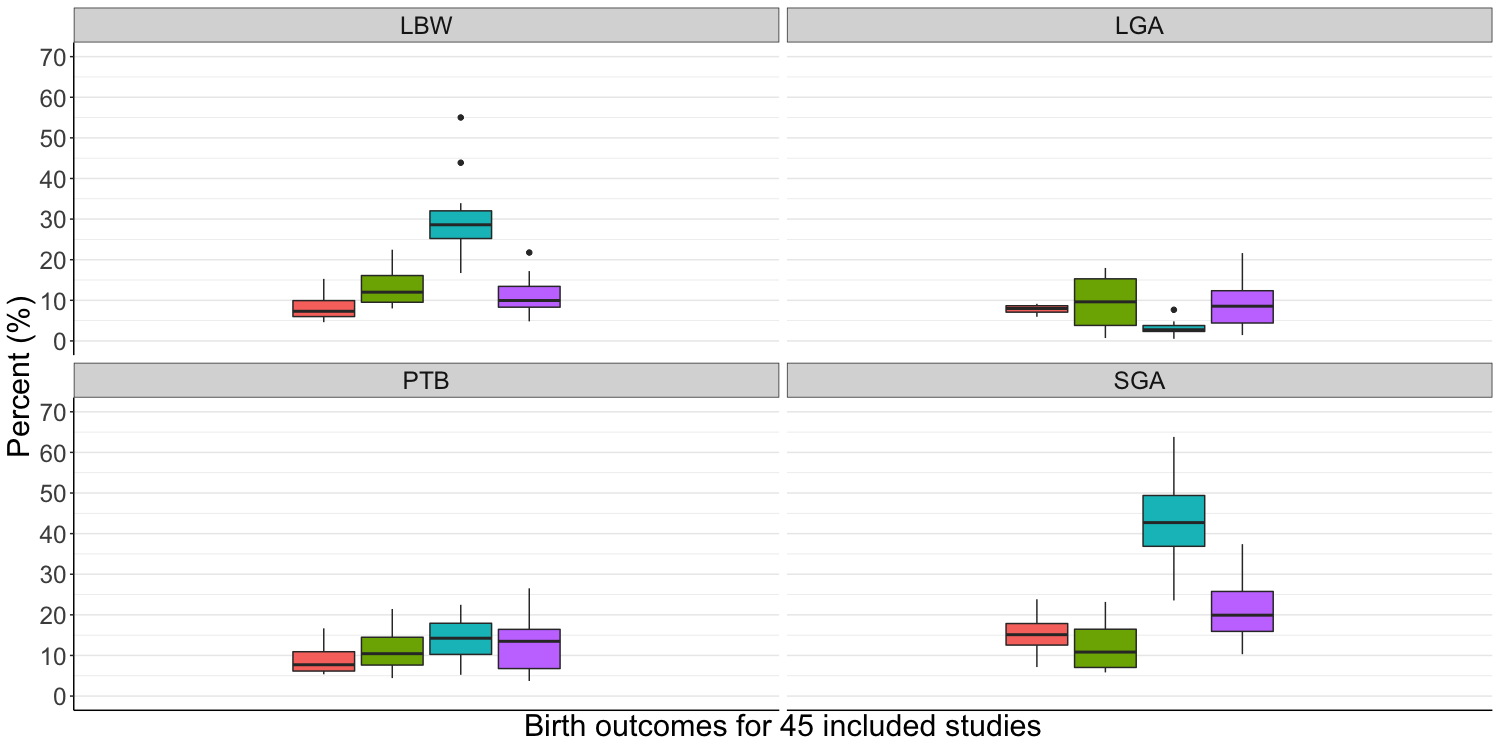
**

**
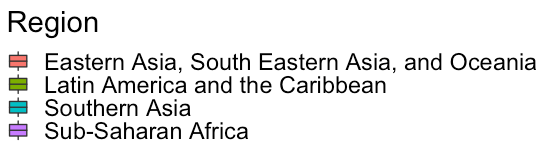
**

S6c: Participant characteristics of livebirths for all 45 studies and by region (n=476,939 live births in the analysis cohort)

| **Variable** | **All studies*** | **Sub-Saharan Africa** | **Southern Asia** | **Eastern Asia, South Eastern Asia, and Oceania** | **Latin America and the Caribbean** |
| --- | --- | --- | --- | --- | --- |
| **Sex** |  |  |  |  |  |
| Male | 244,049 (51.2) | 125,772 (50.9) | 85,208 (51.3) | 27,341 (52.2) | 5,728 (50.2) |
| Female | 232,893 (48.8) | 121,268 (49.1) | 80,944 (48.7) | 25,003 (47.8) | 5,678 (49.8) |
| **Maternal age** |  |  |  |  |  |
| <15 | 2,557 (0.5) | 666 (0.3) | 1,813 (1.1) | 17 (0.0) | 61 (0.5) |
| 15-19 | 69,810 (14.6) | 34,599 (14.0) | 32,288 (19.4) | 1,195 (2.3) | 1,728 (15.1) |
| 20-24 | 143,591 (30.1) | 67,995 (27.5) | 65,914 (39.7) | 6,530 (12.5) | 3,152 (27.6) |
| 25-29 | 121,113 (25.4) | 56,699 (23.0) | 39,417 (23.7) | 22,192 (42.4) | 2,805 (24.6) |
| 30-34 | 79,343 (16.6) | 42,696 (17.3) | 17,855 (10.7) | 16,651 (31.8) | 2,141 (18.8) |
| 35-39 | 39,106 (8.2) | 26,213 (10.6) | 6,742 (4.1) | 5,037 (9.6) | 1,114 (9.8) |
| 40 | 11,238 (2.4) | 8,033 (3.3) | 2,094 (1.3) | 707 (1.4) | 404 (3.5) |
| Missing | 10,184 (2.1) | 10,139 (4.1) | 29 (0.0) | 15 (0.0) | 1 (0.0) |
| **Education** |  |  |  |  |  |
| Primary to secondary | 231,923 (48.6) | 82,869 (33.5) | 143,199 (86.2) | 2,925 (5.6) | 2,930 (25.7) |
| Upper secondary or professional | 173,407 (36.4) | 120,452 (48.8) | 14,953 (9.0) | 37,470 (71.6) | 532 (4.7) |
| Bachelor's and above | 48,717 (10.2) | 38,693 (15.7) | 5,558 (3.3) | 3,672 (7.0) | 794 (7.0) |
| Missing | 22,895 (4.8) | 5,026 (2.0) | 2,442 (1.5) | 8,277 (15.8) | 7,150 (62.7) |
| **Delivery location** |  |  |  |  |  |
| Not health facility | 114,802 (24.1) | 12,826 (5.2) | 101,422 (61.0) | 550 (1.1) | 4 (0.0) |
| Health facility | 353,725 (74.2) | 232,931 (94.3) | 62,424 (37.6) | 51,793 (98.9) | 6,577 (57.7) |
| Missing | 8,415 (1.8) | 1,283 (0.5) | 2,306 (1.4) | 1 (0.0) | 4,825 (42.3) |
| **Delivery type** |  |  |  |  |  |
| Vaginal | 400,173 (83.9) | 202,816 (82.1) | 157,881 (95.0) | 34,434 (65.8) | 5,042 (44.2) |
| Cesarean delivery | 66,556 (14.0) | 40,557 (16.4) | 7,900 (4.8) | 15,997 (30.6) | 2,102 (18.4) |
| Missing | 10,216 (2.1) | 3,670 (1.5) | 371 (0.2) | 1,913 (3.7) | 4,262 (37.4) |
| **Parity** |  |  |  |  |  |
| 1 | 164,128 (34.4) | 79,572 (32.2) | 52,591 (31.7) | 29,859 (57.0) | 2,106 (18.5) |
| 2 | 115,466 (24.2) | 59,881 (24.2) | 40,921 (24.6) | 13,350 (25.5) | 1,314 (11.5) |
| 3 | 67,958 (14.2) | 40,495 (16.4) | 26,415 (15.9) | 578 (1.1) | 470 (4.1) |
| 4 | 38,198 (8.0) | 23,588 (9.5) | 14,250 (8.6) | 161 (0.3) | 199 (1.7) |
| 5 | 49,291 (10.3) | 31,528 (12.8) | 17,452 (10.5) | 145 (0.3) | 166 (1.5) |
| Missing | 41,901 (8.8) | 11,976 (4.8) | 14,523 (8.7) | 8,251 (15.8) | 7,151 (62.7) |
| **Multiple births** |  |  |  |  |  |
| Single | 464,873 (97.5) | 241,337 (97.7) | 161,934 (97.5) | 50,408 (96.3) | 11,194 (98.1) |
| Multiple | 9,815 (2.1) | 5,375 (2.2) | 2,292 (1.4) | 1,936 (3.7) | 212 (1.9) |
| Missing | 2,254 (0.5) | 328 (0.1) | 1,926 (1.2) | 0 (0.0) | 0 (0.0) |
| **Gestational age assessment method** |  |  |  |  |  |
| Ultrasound-based assessment <14 weeks | 1,686 (0.4) | 1,031 (0.4) | 0 (0.0) | 28 (0.1) | 627 (5.5) |
| Ultrasound-based assessment 14-24 weeks | 7,046 (1.5) | 6,179 (2.5) | 0 (0.0) | 614 (1.2) | 253 (2.2) |
| Ultrasound-based assessment >24 weeks | 1,944 (0.4) | 1,297 (0.5) | 0 (0.0) | 641 (1.2) | 6 (0.1) |
| Ultrasound-based assessment timing unknown | 170,277 (35.7) | 105,248 (42.6) | 17,052 (10.3) | 42,249 (80.7) | 5,728 (50.2) |
| Best obstetric estimate | 1,988 (0.4) | 887 (0.4) | 566 (0.3) | 0 (0.0) | 535 (4.7) |
| Last menstrual period method | 277,214 (58.1) | 124,873 (50.5) | 147,373 (88.7) | 4,968 (9.5) | 0 (0.0) |
| Missing | 16,787 (3.5) | 7,525 (3.0) | 1,161 (0.7) | 3,844 (7.3) | 4,257 (37.3) |
|  |  |  |  |  |  |
| * Values are number (percent) |  |  |  |  |  |

S6d: Gestational age assessment method for 45 studies (n=476,939 live births in the analysis cohort)

| **Study** | **Included live births** | **Ultrasound-based assessment <14 weeks*** | **Ultrasound-based assessment 14-24 weeks** | **Ultrasound-based assessment >24 weeks** | **Ultrasound-based assessment timing unknown** | **Best obstetric estimate** | **Last menstrual period method** | **GA assessment method missing** | **GA precision** |
| --- | --- | --- | --- | --- | --- | --- | --- | --- | --- |
| Argentina (2000) | 5,698 | 0 (0.0) | 0 (0.0) | 0 (0.0) | 5,698 (100.0) | 0 (0.0) | 0 (0.0) | 0 (0.0) | Completed weeks |
| Bangladesh (2001) | 13,368 | 0 (0.0) | 0 (0.0) | 0 (0.0) | 0 (0.0) | 0 (0.0) | 13,371 (100.0) | 0 (0.0) | Days |
| Bangladesh (2007) | 20,501 | 0 (0.0) | 0 (0.0) | 0 (0.0) | 0 (0.0) | 0 (0.0) | 20,501 (100.0) | 0 (0.0) | Days |
| Bangladesh (2011) | 18,007 | 0 (0.0) | 0 (0.0) | 0 (0.0) | 0 (0.0) | 0 (0.0) | 18,007 (100.0) | 0 (0.0) | Days |
| Bangladesh (2014) | 2,572 | 0 (0.0) | 0 (0.0) | 0 (0.0) | 2,572 (100.0) | 0 (0.0) | 0 (0.0) | 0 (0.0) | Days |
| Botswana (2014) | 163,928 | 0 (0.0) | 0 (0.0) | 0 (0.0) | 98,678 (60.2) | 0 (0.0) | 65,250 (39.8) | 0 (0.0) | Completed weeks |
| Brazil (2015) | 4,257 | 0 (0.0) | 0 (0.0) | 0 (0.0) | 0 (0.0) | 0 (0.0) | 0 (0.0) | 4,257 (100.0) | Completed weeks |
| Burkina Faso (2004) | 1,045 | 0 (0.0) | 0 (0.0) | 0 (0.0) | 794 (76.0) | 0 (0.0) | 251 (24.0) | 0 (0.0) | Days |
| Burkina Faso (2006) | 1,050 | 0 (0.0) | 0 (0.0) | 0 (0.0) | 873 (83.1) | 0 (0.0) | 177 (16.9) | 0 (0.0) | Days |
| China (2002) | 4,380 | 0 (0.0) | 0 (0.0) | 0 (0.0) | 0 (0.0) | 0 (0.0) | 4,380 (100.0) | 0 (0.0) | Days |
| China (2012) | 42,249 | 0 (0.0) | 0 (0.0) | 0 (0.0) | 42,249 (100.0) | 0 (0.0) | 0 (0.0) | 0 (0.0) | Days |
| Ethiopia (2017) | 544 | 156 (28.7) | 388 (71.3) | 0 (0.0) | 0 (0.0) | 0 (0.0) | 0 (0.0) | 0 (0.0) | Days |
| Ethiopia (2018) | 1,424 | 69 (4.8) | 190 (13.3) | 278 (19.5) | 0 (0.0) | 887 (62.3) | 0 (0.0) | 0 (0.0) | Days |
| Ethiopia (2020) | 556 | 91 (16.4) | 387 (69.6) | 62 (11.2) | 0 (0.0) | 0 (0.0) | 16 (2.9) | 0 (0.0) | Days |
| Ghana (2009) | 1,037 | 168 (16.2) | 757 (73.0) | 112 (10.8) | 0 (0.0) | 0 (0.0) | 0 (0.0) | 0 (0.0) | Days |
| Ghana (2013) | 1,291 | 0 (0.0) | 1,291 (100.0) | 0 (0.0) | 0 (0.0) | 0 (0.0) | 0 (0.0) | 0 (0.0) | Days |
| Guatemala (2013) | 565 | 0 (0.0) | 0 (0.0) | 0 (0.0) | 30 (5.3) | 535 (94.7) | 0 (0.0) | 0 (0.0) | Completed weeks |
| India (2000) | 4,136 | 0 (0.0) | 0 (0.0) | 0 (0.0) | 0 (0.0) | 0 (0.0) | 4,136 (100.0) | 0 (0.0) | Days |
| India (2010) | 44,958 | 0 (0.0) | 0 (0.0) | 0 (0.0) | 0 (0.0) | 0 (0.0) | 44,958 (100.0) | 0 (0.0) | Completed weeks |
| India (2013) | 573 | 0 (0.0) | 0 (0.0) | 0 (0.0) | 7 (1.2) | 566 (98.8) | 0 (0.0) | 0 (0.0) | Completed weeks |
| India (2016) | 653 | 0 (0.0) | 0 (0.0) | 0 (0.0) | 0 (0.0) | 0 (0.0) | 0 (0.0) | 653 (100.0) | Days |
| Malawi (2003) | 1,199 | 0 (0.0) | 1,199 (100.0) | 0 (0.0) | 0 (0.0) | 0 (0.0) | 0 (0.0) | 0 (0.0) | Days |
| Malawi (2011) | 1,074 | 0 (0.0) | 0 (0.0) | 0 (0.0) | 1,074 (100.0) | 0 (0.0) | 0 (0.0) | 0 (0.0) | Days |
| Mexico (2017) | 886 | 627 (70.8) | 253 (28.6) | 6 (0.7) | 0 (0.0) | 0 (0.0) | 0 (0.0) | 0 (0.0) | Days |
| Nepal (2002) | 21,383 | 0 (0.0) | 0 (0.0) | 0 (0.0) | 0 (0.0) | 0 (0.0) | 21,383 (100.0) | 0 (0.0) | Days |
| Nepal (2010) | 23,568 | 0 (0.0) | 0 (0.0) | 0 (0.0) | 0 (0.0) | 0 (0.0) | 23,568 (100.0) | 0 (0.0) | Days |
| Pakistan (2013) | 640 | 0 (0.0) | 0 (0.0) | 0 (0.0) | 132 (20.6) | 0 (0.0) | 0 (0.0) | 508 (79.4) | Completed weeks |
| Pakistan (2014) | 2,415 | 0 (0.0) | 0 (0.0) | 0 (0.0) | 2,415 (100.0) | 0 (0.0) | 0 (0.0) | 0 (0.0) | Days |
| Papua New Guinea (2009) | 1,871 | 28 (1.5) | 614 (32.8) | 641 (34.3) | 0 (0.0) | 0 (0.0) | 588 (31.4) | 0 (0.0) | Days |
| Rwanda (2017) | 2,762 | 524 (19.0) | 1,491 (54.0) | 643 (23.3) | 104 (3.8) | 0 (0.0) | 0 (0.0) | 0 (0.0) | Days |
| South Africa (2016) | 394 | 0 (0.0) | 0 (0.0) | 0 (0.0) | 7 (1.8) | 0 (0.0) | 342 (86.8) | 45 (11.4) | Completed weeks |
| Sri Lanka (2015) | 13,375 | 0 (0.0) | 0 (0.0) | 0 (0.0) | 11,926 (89.2) | 0 (0.0) | 1,449 (10.8) | 0 (0.0) | Days |
| Tanzania (2001) | 7,630 | 0 (0.0) | 0 (0.0) | 0 (0.0) | 0 (0.0) | 0 (0.0) | 7,630 (100.0) | 0 (0.0) | Days |
| Tanzania (2008) | 818 | 0 (0.0) | 0 (0.0) | 0 (0.0) | 0 (0.0) | 0 (0.0) | 0 (0.0) | 818 (100.0) | Days |
| Tanzania (2010) | 8,309 | 0 (0.0) | 0 (0.0) | 0 (0.0) | 0 (0.0) | 0 (0.0) | 8,309 (100.0) | 0 (0.0) | Days |
| Tanzania (2014a) | 407 | 0 (0.0) | 0 (0.0) | 0 (0.0) | 0 (0.0) | 0 (0.0) | 0 (0.0) | 407 (100.0) | Days |
| Tanzania (2014b) | 2,319 | 0 (0.0) | 0 (0.0) | 0 (0.0) | 2,319 (100.0) | 0 (0.0) | 0 (0.0) | 0 (0.0) | Days |
| Thailand (2000) | 3,844 | 0 (0.0) | 0 (0.0) | 0 (0.0) | 0 (0.0) | 0 (0.0) | 0 (0.0) | 3,844 (100.0) | Completed weeks |
| Uganda (2016) | 635 | 0 (0.0) | 0 (0.0) | 0 (0.0) | 635 (100.0) | 0 (0.0) | 0 (0.0) | 0 (0.0) | Completed weeks |
| Uganda (2018) | 6,255 | 0 (0.0) | 0 (0.0) | 0 (0.0) | 0 (0.0) | 0 (0.0) | 0 (0.0) | 6,255 (100.0) | Days |
| Zambia (2011) | 29,207 | 0 (0.0) | 0 (0.0) | 0 (0.0) | 0 (0.0) | 0 (0.0) | 29,207 (100.0) | 0 (0.0) | Completed weeks |
| Zambia (2013) | 703 | 23 (3.3) | 476 (67.7) | 202 (28.7) | 2 (0.3) | 0 (0.0) | 0 (0.0) | 0 (0.0) | Completed weeks |
| Zambia (2014) | 762 | 0 (0.0) | 0 (0.0) | 0 (0.0) | 762 (100.0) | 0 (0.0) | 0 (0.0) | 0 (0.0) | Days |
| Zambia (2015) | 9,509 | 0 (0.0) | 0 (0.0) | 0 (0.0) | 0 (0.0) | 0 (0.0) | 9,509 (100.0) | 0 (0.0) | Days |
| Zimbabwe (2012) | 4,182 | 0 (0.0) | 0 (0.0) | 0 (0.0) | 0 (0.0) | 0 (0.0) | 4,182 (100.0) | 0 (0.0) | Days |

* Values are number (percent)

S6e: Median and interquartile range newborn type prevalence for ten, six, four, and two categories by region*

|  | **10 types** | | | | | | | | | |
| --- | --- | --- | --- | --- | --- | --- | --- | --- | --- | --- |
| **Region** | **T+LGA+nonLBW** | **T+AGA+nonLBW** | **T+AGA+LBW** | **T+SGA+nonLBW** | **T+SGA+LBW** | **PT+LGA+nonLBW** | **PT+LGA+LBW** | **PT+AGA+nonLBW** | **PT+AGA+LBW** | **PT+SGA+LBW** |
| **All studies** | 3.3 (1.0, 5.5) | 58.0 (50.4, 68.6) | 0.5 (0.2, 2.0) | 14.2 (11.0, 19.5) | 7.1 (4.3, 16.6) | 1.6 (0.5, 3.3) | 0.2 (0.1, 0.7) | 3.7 (2.2, 4.8) | 3.6 (2.3, 4.8) | 1.0 (0.7, 2.0) |
| **EA, SEA, O** | 5.7 (4.1, 5.7) | 72.1 (64.2, 72.1) | 0.5 (0.3, 0.5) | 11.0 (6.9, 11.0) | 3.7 (2.4, 3.7) | 2.0 (0.9, 2.0) | 0.3 (0.1, 0.3) | 3.0 (2.2, 3.0) | 2.7 (1.7, 2.7) | 0.6 (0.4, 0.6) |
| **LAC** | 8.0 (2.3, 8.0) | 68.7 (65.3, 68.7) | 0.8 (0.5, 0.8) | 4.2 (3.4, 4.2) | 4.4 (2.2, 4.4) | 0.6 (0.2, 0.6) | 0.1 (0.0, 0.1) | 3.0 (2.1, 3.0) | 5.0 (3.3, 5.0) | 1.0 (0.8, 1.0) |
| **SA** | 0.8 (0.4, 0.8) | 46.3 (35.7, 46.3) | 0.7 (0.6, 0.7) | 20.5 (17.2, 20.5) | 20.3 (17.0, 20.3) | 1.5 (0.6, 1.5) | 0.2 (0.1, 0.2) | 4.2 (3.0, 4.2) | 4.8 (4.4, 4.8) | 2.4 (2.2, 2.4) |
| **SSA** | 4.3 (2.6, 4.3) | 62.2 (56.4, 62.2) | 0.2 (0.1, 0.2) | 13.9 (10.8, 13.9) | 5.2 (4.3, 5.2) | 2.3 (0.5, 2.3) | 0.3 (0.0, 0.3) | 3.7 (2.1, 3.7) | 2.9 (2.1, 2.9) | 0.8 (0.4, 0.8) |

|  | **Six types** | | | | | | **Four types** | | | | |
| --- | --- | --- | --- | --- | --- | --- | --- | --- | --- | --- | --- |
| **Region** | **T+LGA** | **T+AGA** | **T+SGA** | **PT+LGA** | **PT+AGA** | **PT+SGA** | **T+nonSGA** | **T+SGA** | **PT+nonSGA** | **PT+SGA** |  |
| **All studies** | 3.3 (1.0, 5.5) | 58.5 (52.0, 69.1) | 21.9 (15.2, 33.9) | 1.7 (0.5, 4.6) | 7.4 (5.3, 9.6) | 1.0 (0.7, 2.0) | 62.4 (54.7, 74.1) | 21.9 (15.2, 33.9) | 11.1 (6.3, 15.4) | 1.0 (0.7, 2.0) |  |
| **EA, SEA, O** | 5.7 (4.1, 5.8) | 72.4 (64.8, 77.4) | 14.7 (10.2, 19.1) | 2.3 (1.0, 4.6) | 5.7 (4.4, 7.3) | 0.6 (0.4, 1.0) | 78.1 (68.9, 83.1) | 14.7 (10.2, 19.1) | 7.1 (5.4, 12.0) | 0.6 (0.4, 1.0) |  |
| **LAC** | 8.0 (2.3, 14.8) | 69.2 (66.6, 70.8) | 8.5 (5.6, 16.5) | 0.7 (0.2, 1.6) | 8.0 (5.4, 12.9) | 1.0 (0.8, 2.4) | 77.2 (70.6, 83.9) | 8.5 (5.6, 16.5) | 9.4 (5.6, 14.5) | 1.0 (0.8, 2.4) |  |
| **SA** | 0.8 (0.4, 1.7) | 46.8 (36.5, 52.0) | 40.6 (34.2, 47.1) | 1.7 (0.8, 3.3) | 9.4 (7.8, 11.7) | 2.4 (2.2, 3.2) | 47.6 (37.1, 52.8) | 40.6 (34.2, 47.1) | 11.1 (8.6, 14.7) | 2.4 (2.2, 3.2) |  |
| **SSA** | 4.3 (2.6, 5.9) | 62.4 (56.4, 69.4) | 19.3 (15.1, 25.3) | 3.0 (0.5, 6.9) | 6.3 (4.9, 8.7) | 0.8 (0.4, 1.2) | 65.1 (59.9, 74.7) | 19.3 (15.1, 25.3) | 12.5 (5.9, 16.2) | 0.8 (0.4, 1.2) |  |

|  | **Small vs. nonSmall (10 types)** | | **Small vs. nonSmall (6 types)** | |
| --- | --- | --- | --- | --- |
| **Region** | **Small** | **nonSmall** | **Small** | **nonSmall** |
| **All studies** | 38.0 (26.5, 46.0) | 62.0 (54.0, 73.5) | 37.6 (25.9, 45.3) | 62.4 (54.7, 74.1) |
| **EA, SEA, O** | 22.2 (17.3, 31.1) | 77.8 (68.4, 83.1) | 21.9 (16.9, 31.1) | 78.1 (68.9, 83.1) |
| **LAC** | 23.6 (16.6, 29.4) | 76.4 (69.0, 83.9) | 22.8 (16.1, 29.4) | 77.2 (70.6, 83.9) |
| **SA** | 53.1 (48.9, 62.9) | 46.9 (36.4, 52.8) | 52.4 (47.2, 62.9) | 47.6 (37.1, 52.8) |
| **SSA** | 35.2 (25.8, 40.1) | 64.8 (59.7, 74.7) | 34.9 (25.3, 40.1) | 65.1 (59.9, 74.7) |

* Latin America and the Caribbean, Eastern Asia, South Eastern Asia, and Oceania, Southern Asia, Sub-Saharan Africa

S6f: Data quality and missingness for 45 included studies (n=476,939 live births in the analysis cohort)


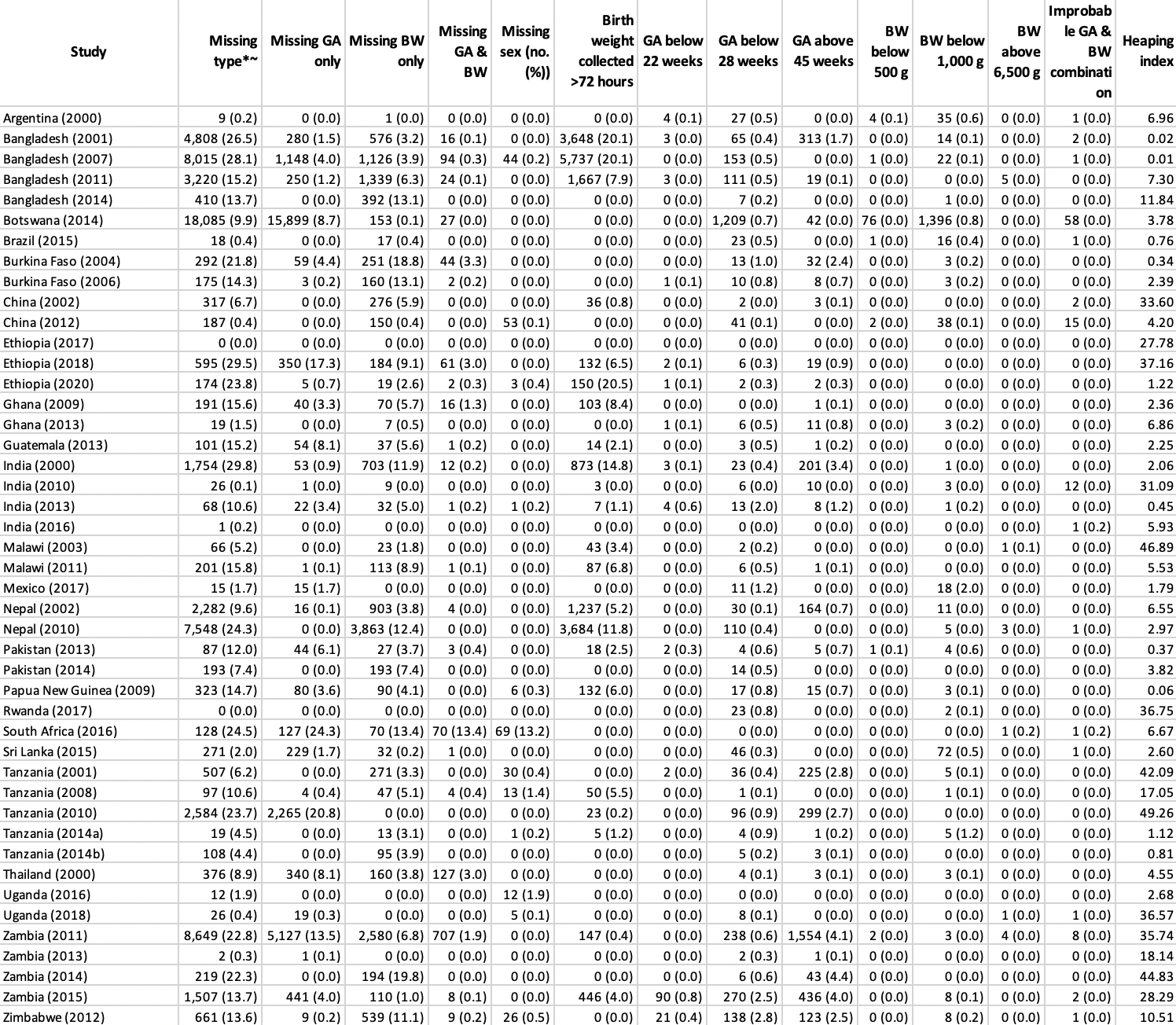


* Missing type refers the percent of live births missing a type value for any reason. ~ Values are number (percent).

S6g: Prevalence of low birthweight, preterm birth, and small- and large-for-gestational age by study

| **Study** | **Live births** | **LBW** | **nonLBW** | **PT** | **T** | **SGA** | **AGA** | **LGA** |
| --- | --- | --- | --- | --- | --- | --- | --- | --- |
| Argentina (2000) | 5698 | 456 (8.0) | 5,242 (92.0) | 496 (8.7) | 5,202 (91.3) | 332 (5.8) | 4,342 (76.2) | 1,024 (18.0) |
| Bangladesh (2001) | 13368 | 7,355 (55.0) | 6,013 (45.0) | 3,004 (22.5) | 10,364 (77.5) | 8,531 (63.8) | 4,339 (32.5) | 498 (3.7) |
| Bangladesh (2007) | 20501 | 8,996 (43.9) | 11,505 (56.1) | 4,177 (20.4) | 16,324 (79.6) | 11,694 (57.0) | 7,819 (38.1) | 988 (4.8) |
| Bangladesh (2011) | 18007 | 5,150 (28.6) | 12,857 (71.4) | 3,502 (19.4) | 14,505 (80.6) | 7,691 (42.7) | 8,935 (49.6) | 1,381 (7.7) |
| Bangladesh (2014) | 2572 | 684 (26.6) | 1,888 (73.4) | 264 (10.3) | 2,308 (89.7) | 1,067 (41.5) | 1,421 (55.2) | 84 (3.3) |
| Botswana (2014) | 163928 | 19,588 (11.9) | 144,340 (88.1) | 23,849 (14.5) | 140,079 (85.5) | 20,325 (12.4) | 125,152 (76.3) | 18,451 (11.3) |
| Brazil (2015) | 4257 | 427 (10.0) | 3,830 (90.0) | 518 (12.2) | 3,739 (87.8) | 317 (7.4) | 3,327 (78.2) | 613 (14.4) |
| Burkina Faso (2004) | 1045 | 179 (17.1) | 866 (82.9) | 149 (14.3) | 896 (85.7) | 289 (27.7) | 702 (67.2) | 54 (5.2) |
| Burkina Faso (2006) | 1050 | 165 (15.7) | 885 (84.3) | 153 (14.6) | 897 (85.4) | 242 (23.0) | 760 (72.4) | 48 (4.6) |
| China (2002) | 4380 | 202 (4.6) | 4,178 (95.4) | 235 (5.4) | 4,145 (94.6) | 695 (15.9) | 3,358 (76.7) | 327 (7.5) |
| China (2012) | 42249 | 2,728 (6.5) | 39,521 (93.5) | 2,714 (6.4) | 39,535 (93.6) | 3,018 (7.1) | 36,720 (86.9) | 2,511 (5.9) |
| Ethiopia (2017) | 544 | 75 (13.8) | 469 (86.2) | 124 (22.8) | 420 (77.2) | 141 (25.9) | 340 (62.5) | 63 (11.6) |
| Ethiopia (2018) | 1424 | 147 (10.3) | 1,277 (89.7) | 181 (12.7) | 1,243 (87.3) | 382 (26.8) | 905 (63.6) | 137 (9.6) |
| Ethiopia (2020) | 556 | 91 (16.4) | 465 (83.6) | 43 (7.7) | 513 (92.3) | 208 (37.4) | 340 (61.2) | 8 (1.4) |
| Ghana (2009) | 1037 | 114 (11.0) | 923 (89.0) | 78 (7.5) | 959 (92.5) | 217 (20.9) | 791 (76.3) | 29 (2.8) |
| Ghana (2013) | 1291 | 222 (17.2) | 1,069 (82.8) | 48 (3.7) | 1,243 (96.3) | 442 (34.2) | 811 (62.8) | 38 (2.9) |
| Guatemala (2013) | 565 | 79 (14.0) | 486 (86.0) | 25 (4.4) | 540 (95.6) | 131 (23.2) | 430 (76.1) | 4 (0.7) |
| India (2000) | 4136 | 1,403 (33.9) | 2,733 (66.1) | 538 (13.0) | 3,598 (87.0) | 2,343 (56.6) | 1,679 (40.6) | 114 (2.8) |
| India (2010) | 44958 | 10,656 (23.7) | 34,302 (76.3) | 7,584 (16.9) | 37,374 (83.1) | 17,193 (38.2) | 26,635 (59.2) | 1,130 (2.5) |
| India (2013) | 573 | 161 (28.1) | 412 (71.9) | 30 (5.2) | 543 (94.8) | 283 (49.4) | 287 (50.1) | 3 (0.5) |
| India (2016) | 653 | 149 (22.8) | 504 (77.2) | 61 (9.3) | 592 (90.7) | 228 (34.9) | 409 (62.6) | 16 (2.5) |
| Malawi (2003) | 1199 | 120 (10.0) | 1,079 (90.0) | 174 (14.5) | 1,025 (85.5) | 204 (17.0) | 937 (78.1) | 58 (4.8) |
| Malawi (2011) | 1074 | 143 (13.3) | 931 (86.7) | 73 (6.8) | 1,001 (93.2) | 257 (23.9) | 788 (73.4) | 29 (2.7) |
| Mexico (2017) | 886 | 199 (22.5) | 687 (77.5) | 190 (21.4) | 696 (78.6) | 126 (14.2) | 717 (80.9) | 43 (4.9) |
| Nepal (2002) | 21383 | 6,491 (30.4) | 14,892 (69.6) | 3,834 (17.9) | 17,549 (82.1) | 10,308 (48.2) | 10,576 (49.5) | 499 (2.3) |
| Nepal (2010) | 23568 | 7,092 (30.1) | 16,476 (69.9) | 3,522 (14.9) | 20,046 (85.1) | 11,430 (48.5) | 11,244 (47.7) | 894 (3.8) |
| Pakistan (2013) | 640 | 205 (32.0) | 435 (68.0) | 87 (13.6) | 553 (86.4) | 236 (36.9) | 393 (61.4) | 11 (1.7) |
| Pakistan (2014) | 2415 | 609 (25.2) | 1,806 (74.8) | 344 (14.2) | 2,071 (85.8) | 828 (34.3) | 1,532 (63.4) | 55 (2.3) |
| Papua New Guinea (2009) | 1871 | 286 (15.3) | 1,585 (84.7) | 312 (16.7) | 1,559 (83.3) | 446 (23.8) | 1,254 (67.0) | 171 (9.1) |
| Rwanda (2017) | 2762 | 133 (4.8) | 2,629 (95.2) | 443 (16.0) | 2,319 (84.0) | 420 (15.2) | 1,852 (67.1) | 490 (17.7) |
| South Africa (2016) | 394 | 39 (9.9) | 355 (90.1) | 31 (7.9) | 363 (92.1) | 49 (12.4) | 309 (78.4) | 36 (9.1) |
| Sri Lanka (2015) | 13375 | 2,235 (16.7) | 11,140 (83.3) | 1,162 (8.7) | 12,213 (91.3) | 3,149 (23.5) | 9,663 (72.2) | 563 (4.2) |
| Tanzania (2001) | 7630 | 666 (8.7) | 6,964 (91.3) | 1,342 (17.6) | 6,288 (82.4) | 1,509 (19.8) | 5,077 (66.5) | 1,044 (13.7) |
| Tanzania (2008) | 818 | 74 (9.0) | 744 (91.0) | 40 (4.9) | 778 (95.1) | 151 (18.5) | 625 (76.4) | 42 (5.1) |
| Tanzania (2010) | 8309 | 708 (8.5) | 7,601 (91.5) | 1,190 (14.3) | 7,119 (85.7) | 2,136 (25.7) | 5,176 (62.3) | 997 (12.0) |
| Tanzania (2014a) | 407 | 51 (12.5) | 356 (87.5) | 25 (6.1) | 382 (93.9) | 118 (29.0) | 277 (68.1) | 12 (2.9) |
| Tanzania (2014b) | 2319 | 148 (6.4) | 2,171 (93.6) | 105 (4.5) | 2,214 (95.5) | 239 (10.3) | 1,768 (76.2) | 312 (13.5) |
| Thailand (2000) | 3844 | 313 (8.1) | 3,531 (91.9) | 346 (9.0) | 3,498 (91.0) | 552 (14.4) | 2,965 (77.1) | 327 (8.5) |
| Uganda (2016) | 635 | 41 (6.5) | 594 (93.5) | 35 (5.5) | 600 (94.5) | 96 (15.1) | 514 (80.9) | 25 (3.9) |
| Uganda (2018) | 6255 | 490 (7.8) | 5,765 (92.2) | 486 (7.8) | 5,769 (92.2) | 884 (14.1) | 4,780 (76.4) | 591 (9.4) |
| Zambia (2011) | 29207 | 2,117 (7.2) | 27,090 (92.8) | 5,472 (18.7) | 23,735 (81.3) | 6,518 (22.3) | 17,537 (60.0) | 5,152 (17.6) |
| Zambia (2013) | 703 | 153 (21.8) | 550 (78.2) | 153 (21.8) | 550 (78.2) | 141 (20.1) | 506 (72.0) | 56 (8.0) |
| Zambia (2014) | 762 | 61 (8.0) | 701 (92.0) | 51 (6.7) | 711 (93.3) | 143 (18.8) | 562 (73.8) | 57 (7.5) |
| Zambia (2015) | 9509 | 800 (8.4) | 8,709 (91.6) | 2,523 (26.5) | 6,986 (73.5) | 1,814 (19.1) | 5,638 (59.3) | 2,057 (21.6) |
| Zimbabwe (2012) | 4182 | 413 (9.9) | 3,769 (90.1) | 966 (23.1) | 3,216 (76.9) | 676 (16.2) | 2,751 (65.8) | 755 (18.1) |

S6h: Prevalence of 10 newborn types overall and by study

**
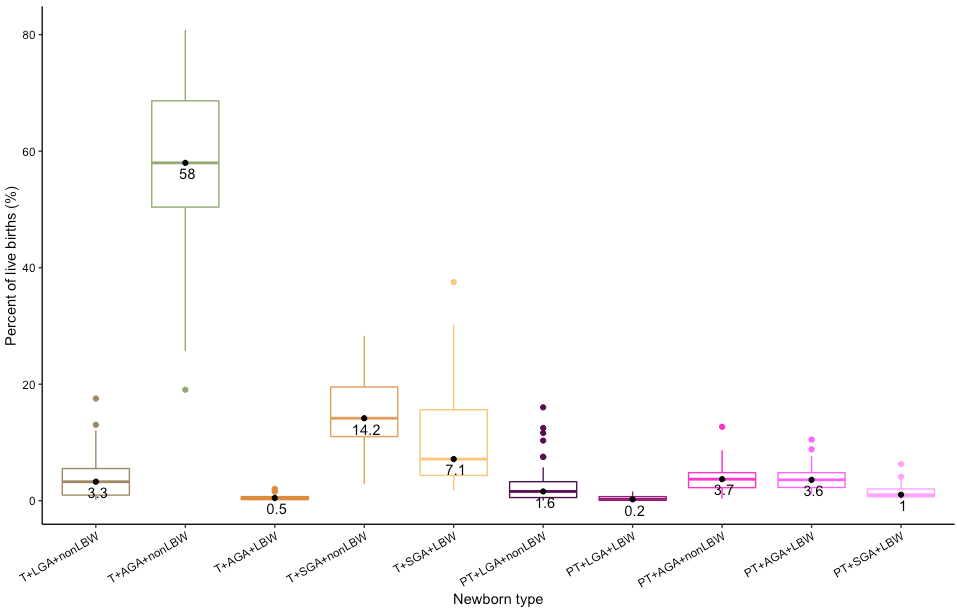
**


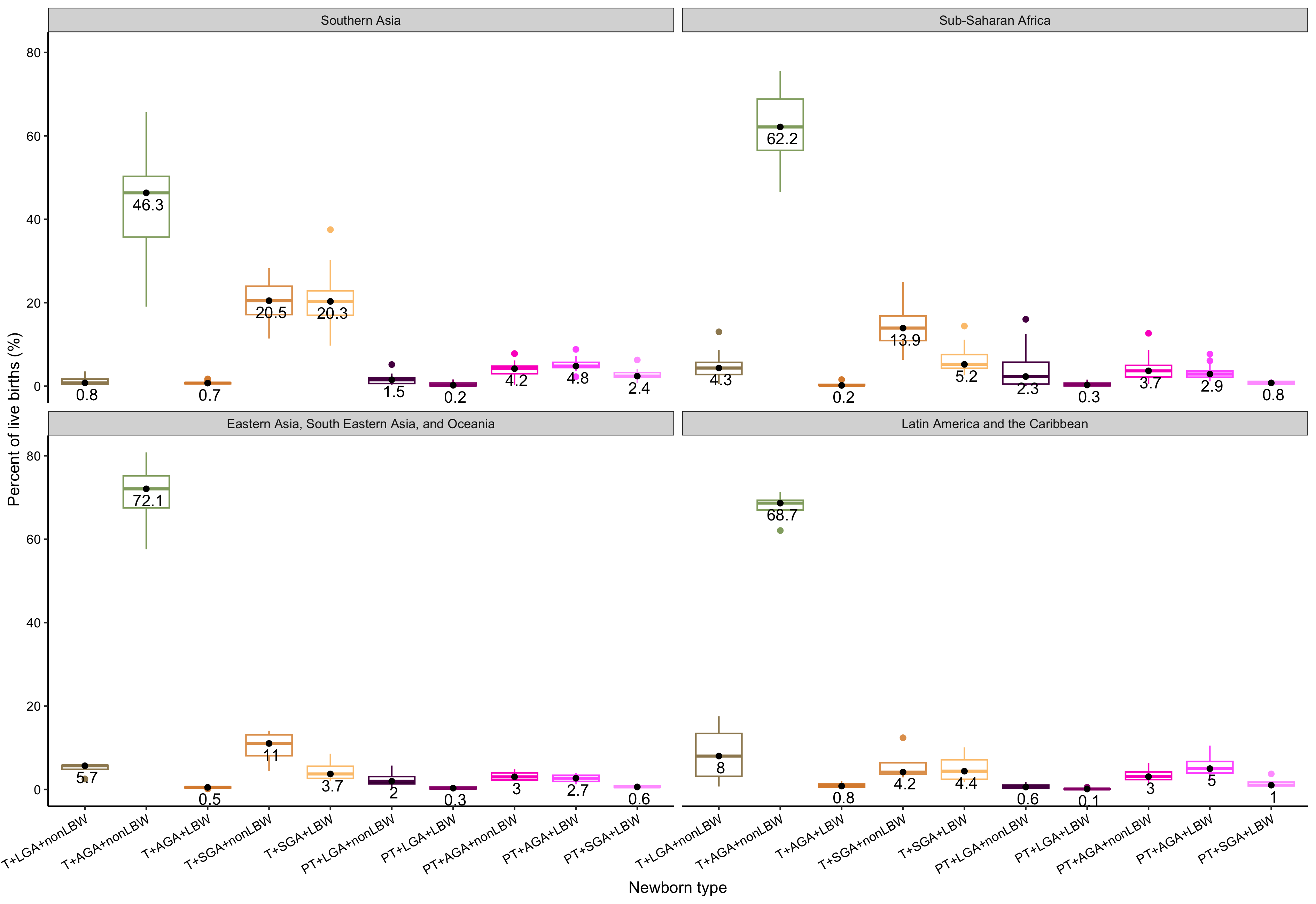


**
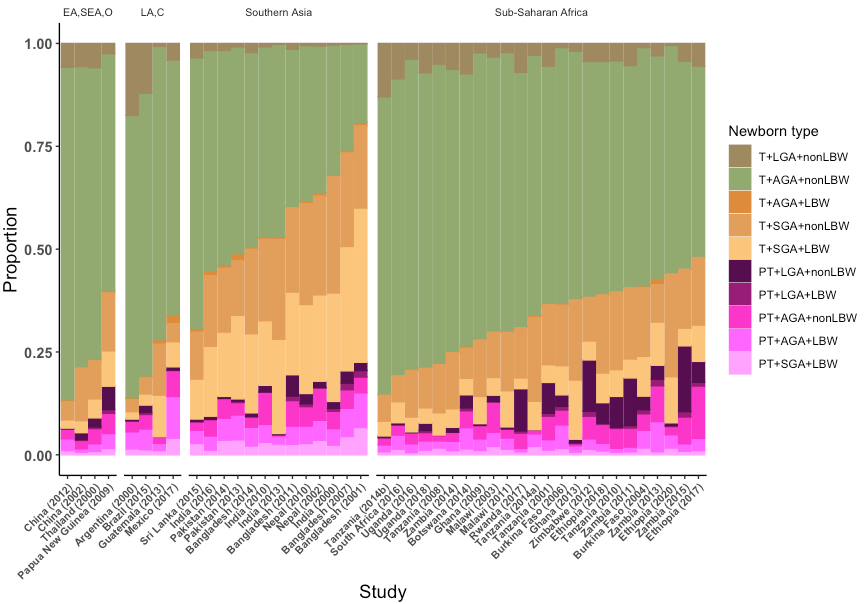
**

| **Study** | **T+LGA+nonLBW** | **T+AGA+nonLBW** | **T+AGA+LBW** | **T+SGA+nonLBW** | **T+SGA+LBW** | **PT+LGA+nonLBW** | **PT+LGA+LBW** | **PT+AGA+nonLBW** | **PT+AGA+LBW** | **PT+SGA+LBW** |
| --- | --- | --- | --- | --- | --- | --- | --- | --- | --- | --- |
| Argentina (2000) | 999 (17.5) | 3,910 (68.6) | 26 (0.5) | 165 (2.9) | 102 (1.8) | 20 (0.4) | 5 (0.1) | 148 (2.6) | 258 (4.5) | 65 (1.1) |
| Bangladesh (2001) | 14 (0.1) | 2,547 (19.1) | 111 (0.8) | 2,677 (20.0) | 5,015 (37.5) | 272 (2.0) | 212 (1.6) | 503 (3.8) | 1,178 (8.8) | 839 (6.3) |
| Bangladesh (2007) | 44 (0.2) | 5,276 (25.7) | 152 (0.7) | 4,653 (22.7) | 6,199 (30.2) | 617 (3.0) | 327 (1.6) | 915 (4.5) | 1,476 (7.2) | 842 (4.1) |
| Bangladesh (2011) | 245 (1.4) | 6,878 (38.2) | 79 (0.4) | 3,690 (20.5) | 3,613 (20.1) | 932 (5.2) | 204 (1.1) | 1,112 (6.2) | 866 (4.8) | 388 (2.2) |
| Bangladesh (2014) | 57 (2.2) | 1,215 (47.2) | 15 (0.6) | 528 (20.5) | 493 (19.2) | 24 (0.9) | 3 (0.1) | 64 (2.5) | 127 (4.9) | 46 (1.8) |
| Botswana (2014) | 12,097 (7.4) | 108,412 (66.1) | 1,041 (0.6) | 11,934 (7.3) | 6,595 (4.0) | 5,218 (3.2) | 1,136 (0.7) | 6,679 (4.1) | 9,020 (5.5) | 1,796 (1.1) |
| Brazil (2015) | 513 (12.1) | 2,924 (68.7) | 23 (0.5) | 166 (3.9) | 113 (2.7) | 78 (1.8) | 22 (0.5) | 149 (3.5) | 231 (5.4) | 38 (0.9) |
| Burkina Faso (2004) | 10 (1.0) | 606 (58.0) | 5 (0.5) | 174 (16.7) | 101 (9.7) | 34 (3.3) | 10 (1.0) | 42 (4.0) | 49 (4.7) | 14 (1.3) |
| Burkina Faso (2006) | 10 (1.0) | 654 (62.3) | 4 (0.4) | 154 (14.7) | 75 (7.1) | 29 (2.8) | 9 (0.9) | 38 (3.6) | 64 (6.1) | 13 (1.2) |
| China (2002) | 245 (5.6) | 3,211 (73.3) | 5 (0.1) | 559 (12.8) | 125 (2.9) | 75 (1.7) | 7 (0.2) | 88 (2.0) | 54 (1.2) | 11 (0.3) |
| China (2012) | 2,433 (5.8) | 34,147 (80.8) | 221 (0.5) | 1,879 (4.4) | 855 (2.0) | 64 (0.2) | 14 (0.0) | 998 (2.4) | 1,354 (3.2) | 284 (0.7) |
| Ethiopia (2017) | 30 (5.5) | 253 (46.5) | 0 (0.0) | 89 (16.4) | 48 (8.8) | 28 (5.1) | 5 (0.9) | 69 (12.7) | 18 (3.3) | 4 (0.7) |
| Ethiopia (2018) | 62 (4.4) | 807 (56.7) | 1 (0.1) | 271 (19.0) | 102 (7.2) | 71 (5.0) | 4 (0.3) | 66 (4.6) | 31 (2.2) | 9 (0.6) |
| Ethiopia (2020) | 3 (0.5) | 308 (55.4) | 1 (0.2) | 139 (25.0) | 62 (11.2) | 4 (0.7) | 1 (0.2) | 11 (2.0) | 20 (3.6) | 7 (1.3) |
| Ghana (2009) | 24 (2.3) | 720 (69.4) | 5 (0.5) | 142 (13.7) | 68 (6.6) | 4 (0.4) | 1 (0.1) | 33 (3.2) | 33 (3.2) | 7 (0.7) |
| Ghana (2013) | 24 (1.9) | 776 (60.1) | 5 (0.4) | 252 (19.5) | 186 (14.4) | 11 (0.9) | 3 (0.2) | 6 (0.5) | 24 (1.9) | 4 (0.3) |
| Guatemala (2013) | 4 (0.7) | 403 (71.3) | 6 (1.1) | 70 (12.4) | 57 (10.1) | 0 (0.0) | 0 (0.0) | 9 (1.6) | 12 (2.1) | 4 (0.7) |
| India (2000) | 16 (0.4) | 1,303 (31.5) | 19 (0.5) | 1,170 (28.3) | 1,090 (26.4) | 71 (1.7) | 27 (0.7) | 173 (4.2) | 184 (4.4) | 83 (2.0) |
| India (2010) | 364 (0.8) | 20,834 (46.3) | 208 (0.5) | 8,901 (19.8) | 7,067 (15.7) | 705 (1.6) | 61 (0.1) | 3,498 (7.8) | 2,095 (4.7) | 1,225 (2.7) |
| India (2013) | 1 (0.2) | 268 (46.8) | 4 (0.7) | 139 (24.3) | 131 (22.9) | 2 (0.3) | 0 (0.0) | 2 (0.3) | 13 (2.3) | 13 (2.3) |
| India (2016) | 11 (1.7) | 350 (53.6) | 8 (1.2) | 112 (17.2) | 111 (17.0) | 5 (0.8) | 0 (0.0) | 26 (4.0) | 25 (3.8) | 5 (0.8) |
| Malawi (2003) | 39 (3.3) | 800 (66.7) | 2 (0.2) | 132 (11.0) | 52 (4.3) | 19 (1.6) | 0 (0.0) | 89 (7.4) | 46 (3.8) | 20 (1.7) |
| Malawi (2011) | 24 (2.2) | 726 (67.6) | 5 (0.5) | 152 (14.2) | 94 (8.8) | 5 (0.5) | 0 (0.0) | 24 (2.2) | 33 (3.1) | 11 (1.0) |
| Mexico (2017) | 35 (4.0) | 550 (62.1) | 18 (2.0) | 39 (4.4) | 54 (6.1) | 7 (0.8) | 1 (0.1) | 56 (6.3) | 93 (10.5) | 33 (3.7) |
| Nepal (2002) | 130 (0.6) | 7,644 (35.7) | 161 (0.8) | 5,124 (24.0) | 4,490 (21.0) | 325 (1.5) | 44 (0.2) | 1,669 (7.8) | 1,102 (5.2) | 694 (3.2) |
| Nepal (2010) | 124 (0.5) | 8,887 (37.7) | 171 (0.7) | 5,771 (24.5) | 5,093 (21.6) | 594 (2.5) | 176 (0.7) | 1,100 (4.7) | 1,086 (4.6) | 566 (2.4) |
| Pakistan (2013) | 5 (0.8) | 322 (50.3) | 11 (1.7) | 85 (13.3) | 130 (20.3) | 4 (0.6) | 2 (0.3) | 19 (3.0) | 41 (6.4) | 21 (3.3) |
| Pakistan (2014) | 42 (1.7) | 1,261 (52.2) | 17 (0.7) | 374 (15.5) | 377 (15.6) | 13 (0.5) | 0 (0.0) | 116 (4.8) | 138 (5.7) | 77 (3.2) |
| Papua New Guinea (2009) | 47 (2.5) | 1,077 (57.6) | 12 (0.6) | 263 (14.1) | 160 (8.6) | 107 (5.7) | 17 (0.9) | 91 (4.9) | 74 (4.0) | 23 (1.2) |
| Rwanda (2017) | 192 (7.0) | 1,713 (62.0) | 5 (0.2) | 337 (12.2) | 72 (2.6) | 285 (10.3) | 13 (0.5) | 102 (3.7) | 32 (1.2) | 11 (0.4) |
| South Africa (2016) | 34 (8.6) | 284 (72.1) | 0 (0.0) | 25 (6.3) | 20 (5.1) | 2 (0.5) | 0 (0.0) | 10 (2.5) | 15 (3.8) | 4 (1.0) |
| Sri Lanka (2015) | 472 (3.5) | 8,788 (65.7) | 125 (0.9) | 1,528 (11.4) | 1,300 (9.7) | 81 (0.6) | 10 (0.1) | 271 (2.0) | 479 (3.6) | 321 (2.4) |
| Tanzania (2001) | 419 (5.5) | 4,420 (57.9) | 4 (0.1) | 1,116 (14.6) | 329 (4.3) | 574 (7.5) | 51 (0.7) | 435 (5.7) | 218 (2.9) | 64 (0.8) |
| Tanzania (2008) | 41 (5.0) | 596 (72.9) | 1 (0.1) | 96 (11.7) | 44 (5.4) | 0 (0.0) | 1 (0.1) | 11 (1.3) | 17 (2.1) | 11 (1.3) |
| Tanzania (2010) | 348 (4.2) | 4,661 (56.1) | 3 (0.0) | 1,567 (18.9) | 540 (6.5) | 625 (7.5) | 24 (0.3) | 400 (4.8) | 112 (1.3) | 29 (0.3) |
| Tanzania (2014a) | 12 (2.9) | 257 (63.1) | 2 (0.5) | 83 (20.4) | 28 (6.9) | 0 (0.0) | 0 (0.0) | 4 (1.0) | 14 (3.4) | 7 (1.7) |
| Tanzania (2014b) | 302 (13.0) | 1,678 (72.4) | 5 (0.2) | 146 (6.3) | 83 (3.6) | 6 (0.3) | 4 (0.2) | 39 (1.7) | 46 (2.0) | 10 (0.4) |
| Thailand (2000) | 225 (5.9) | 2,723 (70.8) | 18 (0.5) | 357 (9.3) | 175 (4.6) | 85 (2.2) | 17 (0.4) | 141 (3.7) | 83 (2.2) | 20 (0.5) |
| Uganda (2016) | 24 (3.8) | 480 (75.6) | 1 (0.2) | 72 (11.3) | 23 (3.6) | 1 (0.2) | 0 (0.0) | 17 (2.7) | 16 (2.5) | 1 (0.2) |
| Uganda (2018) | 444 (7.1) | 4,478 (71.6) | 13 (0.2) | 601 (9.6) | 233 (3.7) | 115 (1.8) | 32 (0.5) | 127 (2.0) | 162 (2.6) | 50 (0.8) |
| Zambia (2011) | 1,578 (5.4) | 15,705 (53.8) | 54 (0.2) | 5,062 (17.3) | 1,336 (4.6) | 3,396 (11.6) | 178 (0.6) | 1,349 (4.6) | 429 (1.5) | 120 (0.4) |
| Zambia (2013) | 21 (3.0) | 380 (54.1) | 11 (1.6) | 64 (9.1) | 74 (10.5) | 24 (3.4) | 11 (1.6) | 61 (8.7) | 54 (7.7) | 3 (0.4) |
| Zambia (2014) | 48 (6.3) | 523 (68.6) | 2 (0.3) | 104 (13.6) | 34 (4.5) | 9 (1.2) | 0 (0.0) | 17 (2.2) | 20 (2.6) | 5 (0.7) |
| Zambia (2015) | 413 (4.3) | 4,792 (50.4) | 16 (0.2) | 1,360 (14.3) | 405 (4.3) | 1,524 (16.0) | 120 (1.3) | 620 (6.5) | 210 (2.2) | 49 (0.5) |
| Zimbabwe (2012) | 182 (4.4) | 2,392 (57.2) | 7 (0.2) | 444 (10.6) | 191 (4.6) | 522 (12.5) | 51 (1.2) | 229 (5.5) | 123 (2.9) | 41 (1.0) |

S6i: Prevalence of 6 newborn types by study


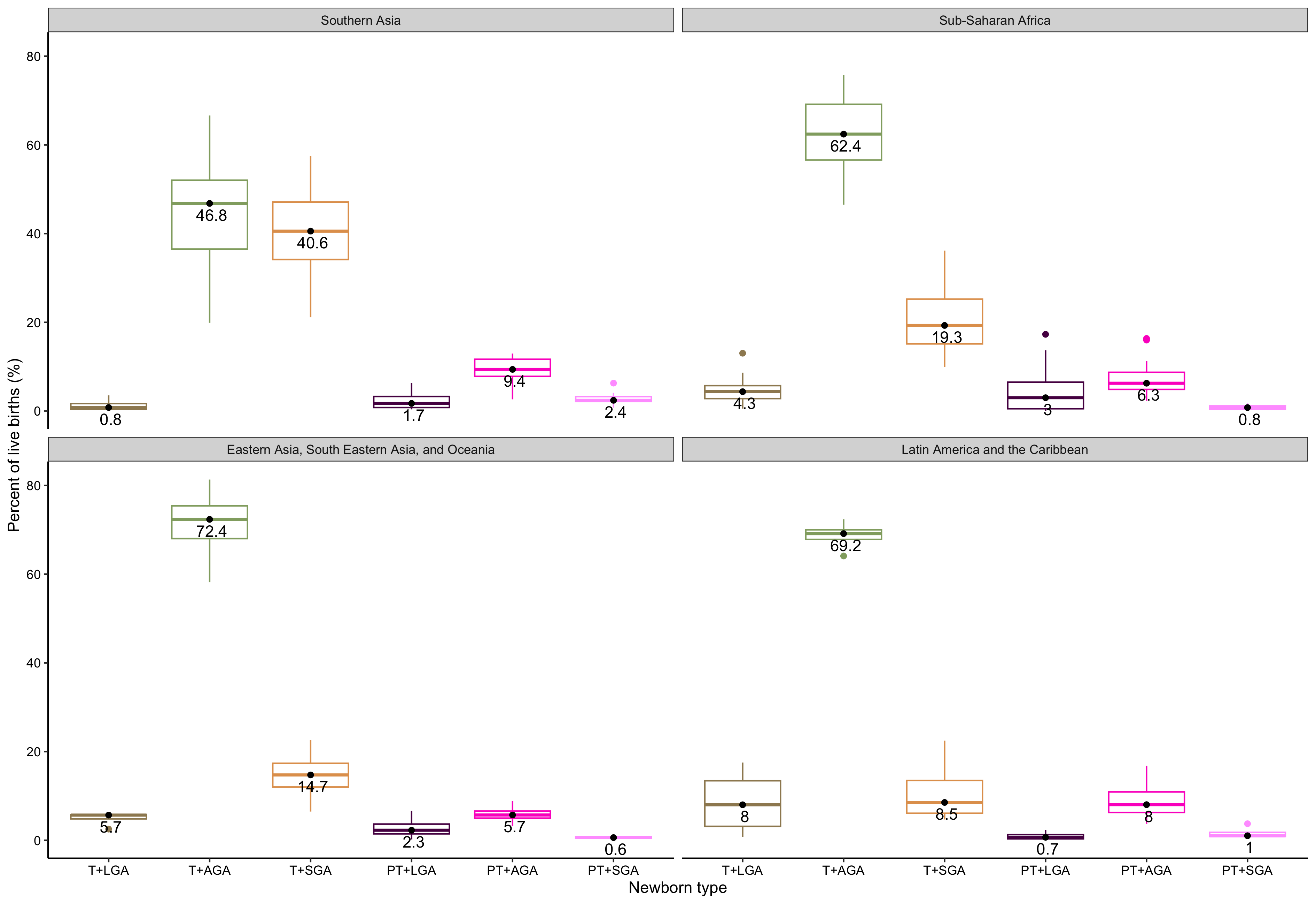


| **Study** | **T+LGA** | **T+AGA** | **T+SGA** | **PT+LGA** | **PT+AGA** | **PT+SGA** |
| --- | --- | --- | --- | --- | --- | --- |
| Argentina (2000) | 999 (17.5) | 3,936 (69.1) | 267 (4.7) | 25 (0.4) | 406 (7.1) | 65 (1.1) |
| Bangladesh (2001) | 14 (0.1) | 2,658 (19.9) | 7,692 (57.5) | 484 (3.6) | 1,681 (12.6) | 839 (6.3) |
| Bangladesh (2007) | 44 (0.2) | 5,428 (26.5) | 10,852 (52.9) | 944 (4.6) | 2,391 (11.7) | 842 (4.1) |
| Bangladesh (2011) | 245 (1.4) | 6,957 (38.6) | 7,303 (40.6) | 1,136 (6.3) | 1,978 (11.0) | 388 (2.2) |
| Bangladesh (2014) | 57 (2.2) | 1,230 (47.8) | 1,021 (39.7) | 27 (1.0) | 191 (7.4) | 46 (1.8) |
| Botswana (2014) | 12,097 (7.4) | 109,453 (66.8) | 18,529 (11.3) | 6,354 (3.9) | 15,699 (9.6) | 1,796 (1.1) |
| Brazil (2015) | 513 (12.1) | 2,947 (69.2) | 279 (6.6) | 100 (2.3) | 380 (8.9) | 38 (0.9) |
| Burkina Faso (2004) | 10 (1.0) | 611 (58.5) | 275 (26.3) | 44 (4.2) | 91 (8.7) | 14 (1.3) |
| Burkina Faso (2006) | 10 (1.0) | 658 (62.7) | 229 (21.8) | 38 (3.6) | 102 (9.7) | 13 (1.2) |
| China (2002) | 245 (5.6) | 3,216 (73.4) | 684 (15.6) | 82 (1.9) | 142 (3.2) | 11 (0.3) |
| China (2012) | 2,433 (5.8) | 34,368 (81.3) | 2,734 (6.5) | 78 (0.2) | 2,352 (5.6) | 284 (0.7) |
| Ethiopia (2017) | 30 (5.5) | 253 (46.5) | 137 (25.2) | 33 (6.1) | 87 (16.0) | 4 (0.7) |
| Ethiopia (2018) | 62 (4.4) | 808 (56.7) | 373 (26.2) | 75 (5.3) | 97 (6.8) | 9 (0.6) |
| Ethiopia (2020) | 3 (0.5) | 309 (55.6) | 201 (36.2) | 5 (0.9) | 31 (5.6) | 7 (1.3) |
| Ghana (2009) | 24 (2.3) | 725 (69.9) | 210 (20.3) | 5 (0.5) | 66 (6.4) | 7 (0.7) |
| Ghana (2013) | 24 (1.9) | 781 (60.5) | 438 (33.9) | 14 (1.1) | 30 (2.3) | 4 (0.3) |
| Guatemala (2013) | 4 (0.7) | 409 (72.4) | 127 (22.5) | 0 (0.0) | 21 (3.7) | 4 (0.7) |
| India (2000) | 16 (0.4) | 1,322 (32.0) | 2,260 (54.6) | 98 (2.4) | 357 (8.6) | 83 (2.0) |
| India (2010) | 364 (0.8) | 21,042 (46.8) | 15,968 (35.5) | 766 (1.7) | 5,593 (12.4) | 1,225 (2.7) |
| India (2013) | 1 (0.2) | 272 (47.5) | 270 (47.1) | 2 (0.3) | 15 (2.6) | 13 (2.3) |
| India (2016) | 11 (1.7) | 358 (54.8) | 223 (34.2) | 5 (0.8) | 51 (7.8) | 5 (0.8) |
| Malawi (2003) | 39 (3.3) | 802 (66.9) | 184 (15.3) | 19 (1.6) | 135 (11.3) | 20 (1.7) |
| Malawi (2011) | 24 (2.2) | 731 (68.1) | 246 (22.9) | 5 (0.5) | 57 (5.3) | 11 (1.0) |
| Mexico (2017) | 35 (4.0) | 568 (64.1) | 93 (10.5) | 8 (0.9) | 149 (16.8) | 33 (3.7) |
| Nepal (2002) | 130 (0.6) | 7,805 (36.5) | 9,614 (45.0) | 369 (1.7) | 2,771 (13.0) | 694 (3.2) |
| Nepal (2010) | 124 (0.5) | 9,058 (38.4) | 10,864 (46.1) | 770 (3.3) | 2,186 (9.3) | 566 (2.4) |
| Pakistan (2013) | 5 (0.8) | 333 (52.0) | 215 (33.6) | 6 (0.9) | 60 (9.4) | 21 (3.3) |
| Pakistan (2014) | 42 (1.7) | 1,278 (52.9) | 751 (31.1) | 13 (0.5) | 254 (10.5) | 77 (3.2) |
| Papua New Guinea (2009) | 47 (2.5) | 1,089 (58.2) | 423 (22.6) | 124 (6.6) | 165 (8.8) | 23 (1.2) |
| Rwanda (2017) | 192 (7.0) | 1,718 (62.2) | 409 (14.8) | 298 (10.8) | 134 (4.9) | 11 (0.4) |
| South Africa (2016) | 34 (8.6) | 284 (72.1) | 45 (11.4) | 2 (0.5) | 25 (6.3) | 4 (1.0) |
| Sri Lanka (2015) | 472 (3.5) | 8,913 (66.6) | 2,828 (21.1) | 91 (0.7) | 750 (5.6) | 321 (2.4) |
| Tanzania (2001) | 419 (5.5) | 4,424 (58.0) | 1,445 (18.9) | 625 (8.2) | 653 (8.6) | 64 (0.8) |
| Tanzania (2008) | 41 (5.0) | 597 (73.0) | 140 (17.1) | 1 (0.1) | 28 (3.4) | 11 (1.3) |
| Tanzania (2010) | 348 (4.2) | 4,664 (56.1) | 2,107 (25.4) | 649 (7.8) | 512 (6.2) | 29 (0.3) |
| Tanzania (2014a) | 12 (2.9) | 259 (63.6) | 111 (27.3) | 0 (0.0) | 18 (4.4) | 7 (1.7) |
| Tanzania (2014b) | 302 (13.0) | 1,683 (72.6) | 229 (9.9) | 10 (0.4) | 85 (3.7) | 10 (0.4) |
| Thailand (2000) | 225 (5.9) | 2,741 (71.3) | 532 (13.8) | 102 (2.7) | 224 (5.8) | 20 (0.5) |
| Uganda (2016) | 24 (3.8) | 481 (75.7) | 95 (15.0) | 1 (0.2) | 33 (5.2) | 1 (0.2) |
| Uganda (2018) | 444 (7.1) | 4,491 (71.8) | 834 (13.3) | 147 (2.4) | 289 (4.6) | 50 (0.8) |
| Zambia (2011) | 1,578 (5.4) | 15,759 (54.0) | 6,398 (21.9) | 3,574 (12.2) | 1,778 (6.1) | 120 (0.4) |
| Zambia (2013) | 21 (3.0) | 391 (55.6) | 138 (19.6) | 35 (5.0) | 115 (16.4) | 3 (0.4) |
| Zambia (2014) | 48 (6.3) | 525 (68.9) | 138 (18.1) | 9 (1.2) | 37 (4.9) | 5 (0.7) |
| Zambia (2015) | 413 (4.3) | 4,808 (50.6) | 1,765 (18.6) | 1,644 (17.3) | 830 (8.7) | 49 (0.5) |
| Zimbabwe (2012) | 182 (4.4) | 2,399 (57.4) | 635 (15.2) | 573 (13.7) | 352 (8.4) | 41 (1.0) |

S6j: Six newborn types sorted by prevalence in descending order for each type category color-coded by geographical region


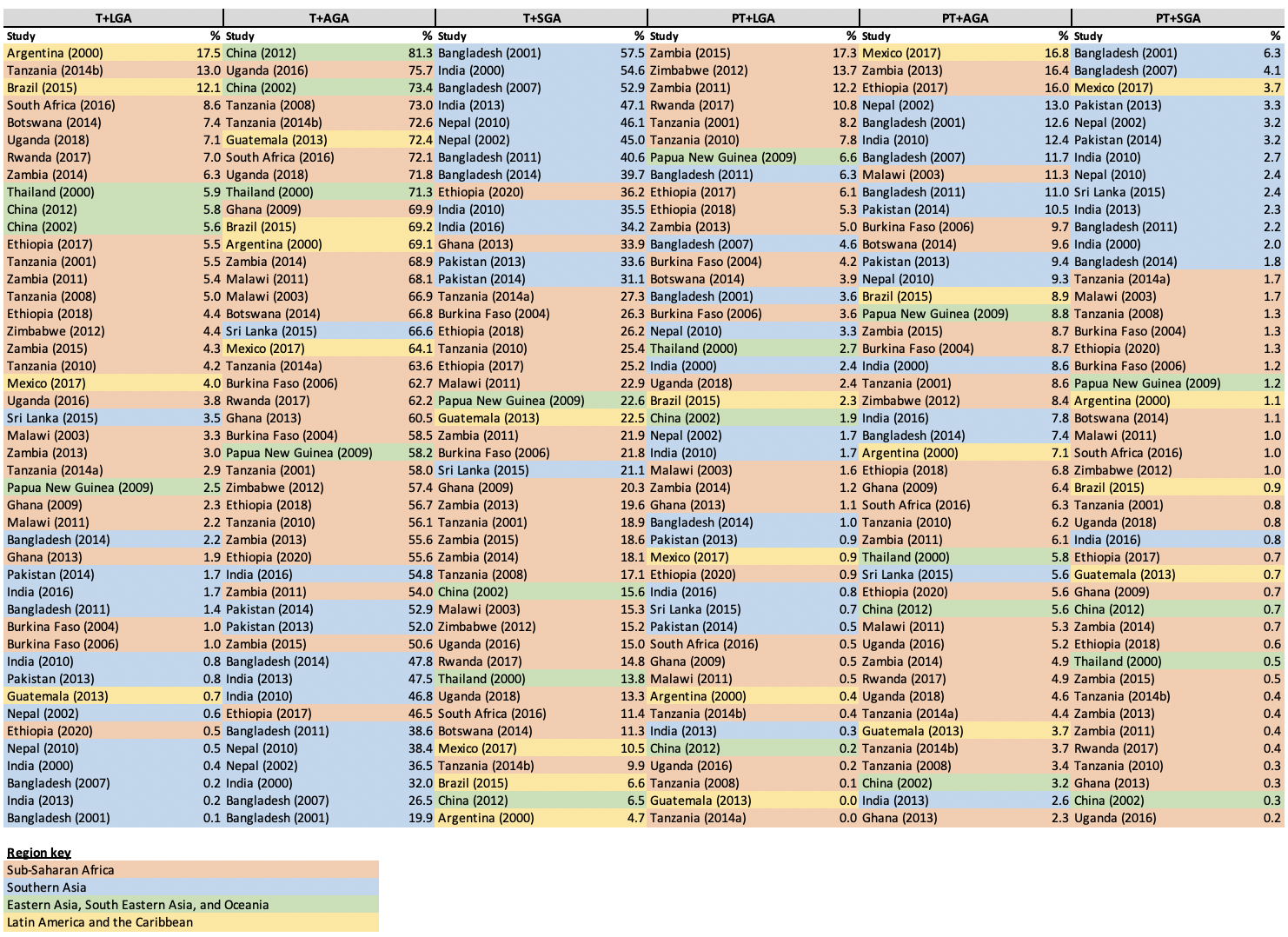


S6k: Prevalence of 4 newborn types overall and by study


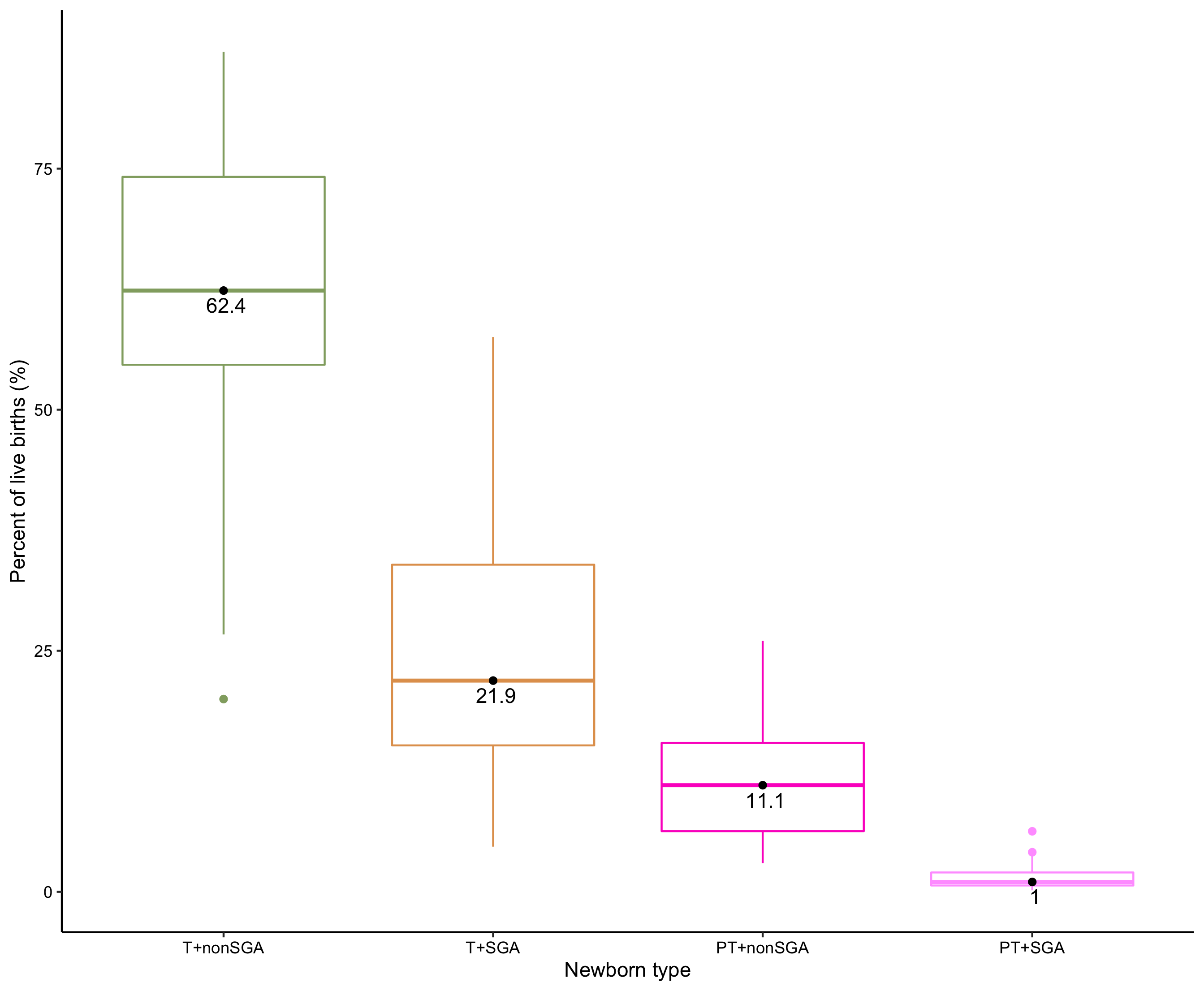


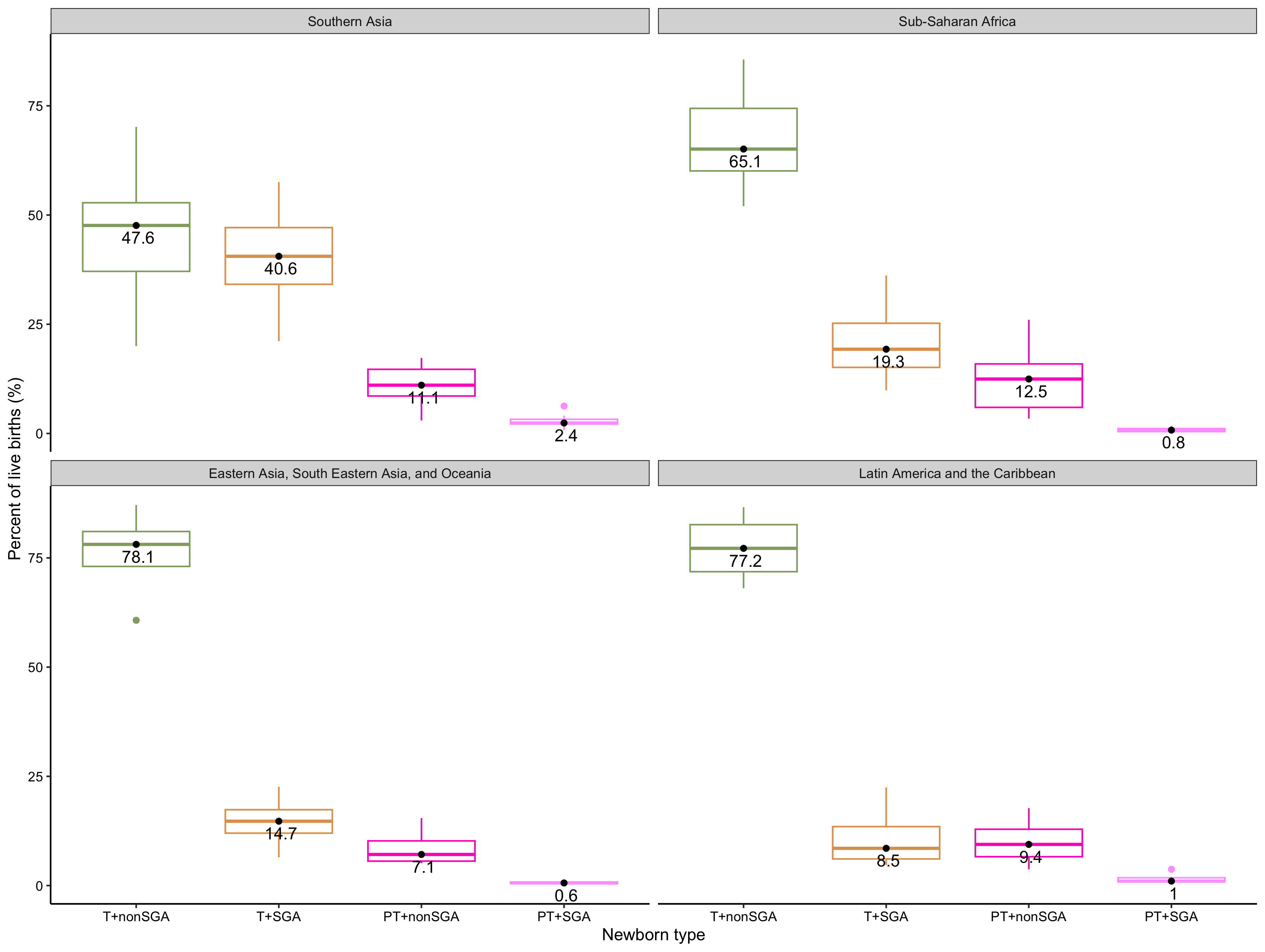


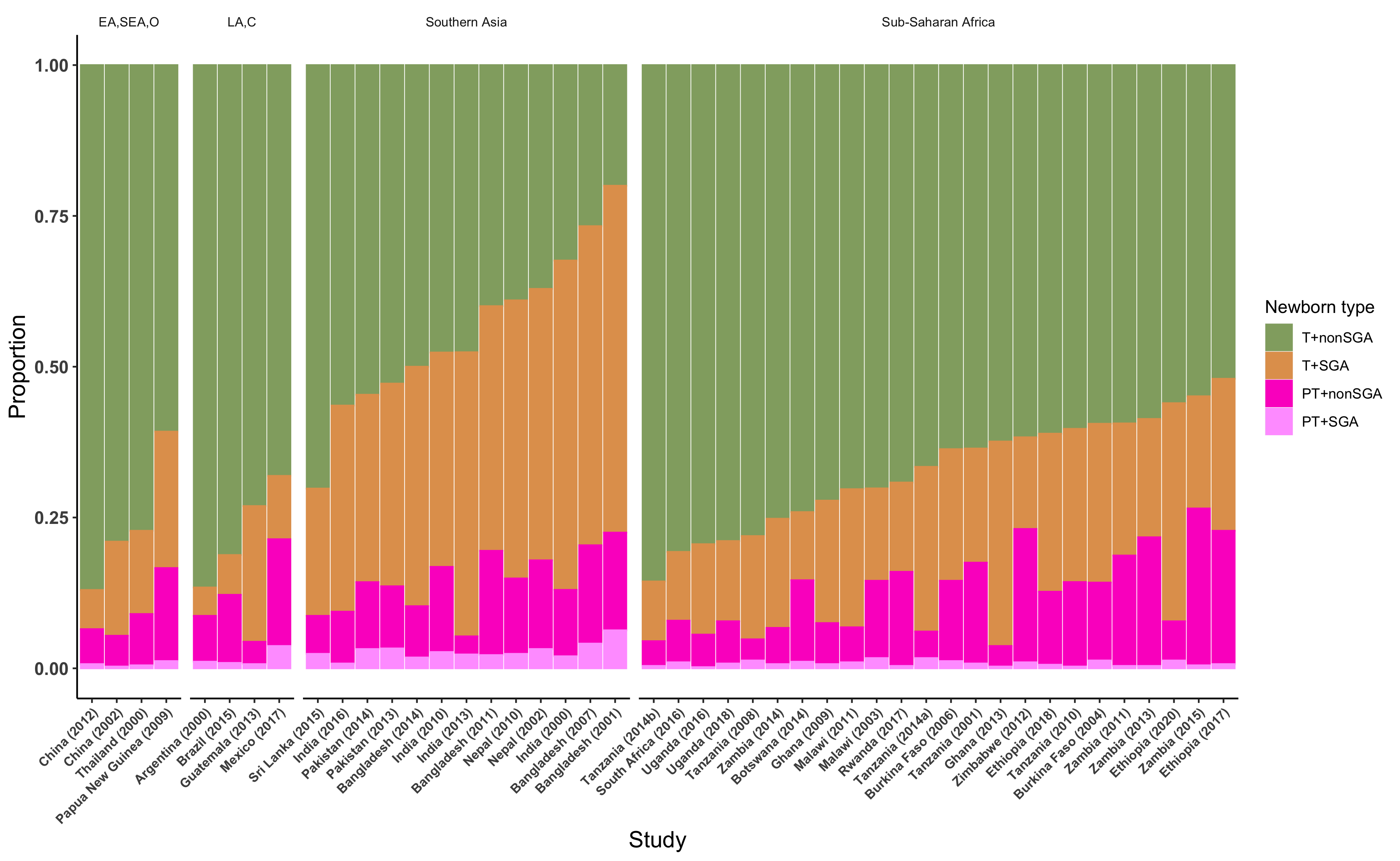


| **Study** | **T+nonSGA** | **T+SGA** | **PT+nonSGA** | **PT+SGA** |
| --- | --- | --- | --- | --- |
| Argentina (2000) | 4,935 (86.6) | 267 (4.7) | 431 (7.6) | 65 (1.1) |
| Bangladesh (2001) | 2,672 (20.0) | 7,692 (57.5) | 2,165 (16.2) | 839 (6.3) |
| Bangladesh (2007) | 5,472 (26.7) | 10,852 (52.9) | 3,335 (16.3) | 842 (4.1) |
| Bangladesh (2011) | 7,202 (40.0) | 7,303 (40.6) | 3,114 (17.3) | 388 (2.2) |
| Bangladesh (2014) | 1,287 (50.0) | 1,021 (39.7) | 218 (8.5) | 46 (1.8) |
| Botswana (2014) | 121,550 (74.1) | 18,529 (11.3) | 22,053 (13.5) | 1,796 (1.1) |
| Brazil (2015) | 3,460 (81.3) | 279 (6.6) | 480 (11.3) | 38 (0.9) |
| Burkina Faso (2004) | 621 (59.4) | 275 (26.3) | 135 (12.9) | 14 (1.3) |
| Burkina Faso (2006) | 668 (63.6) | 229 (21.8) | 140 (13.3) | 13 (1.2) |
| China (2002) | 3,461 (79.0) | 684 (15.6) | 224 (5.1) | 11 (0.3) |
| China (2012) | 36,801 (87.1) | 2,734 (6.5) | 2,430 (5.8) | 284 (0.7) |
| Ethiopia (2017) | 283 (52.0) | 137 (25.2) | 120 (22.1) | 4 (0.7) |
| Ethiopia (2018) | 870 (61.1) | 373 (26.2) | 172 (12.1) | 9 (0.6) |
| Ethiopia (2020) | 312 (56.1) | 201 (36.2) | 36 (6.5) | 7 (1.3) |
| Ghana (2009) | 749 (72.2) | 210 (20.3) | 71 (6.8) | 7 (0.7) |
| Ghana (2013) | 805 (62.4) | 438 (33.9) | 44 (3.4) | 4 (0.3) |
| Guatemala (2013) | 413 (73.1) | 127 (22.5) | 21 (3.7) | 4 (0.7) |
| India (2000) | 1,338 (32.4) | 2,260 (54.6) | 455 (11.0) | 83 (2.0) |
| India (2010) | 21,406 (47.6) | 15,968 (35.5) | 6,359 (14.1) | 1,225 (2.7) |
| India (2013) | 273 (47.6) | 270 (47.1) | 17 (3.0) | 13 (2.3) |
| India (2016) | 369 (56.5) | 223 (34.2) | 56 (8.6) | 5 (0.8) |
| Malawi (2003) | 841 (70.1) | 184 (15.3) | 154 (12.8) | 20 (1.7) |
| Malawi (2011) | 755 (70.3) | 246 (22.9) | 62 (5.8) | 11 (1.0) |
| Mexico (2017) | 603 (68.1) | 93 (10.5) | 157 (17.7) | 33 (3.7) |
| Nepal (2002) | 7,935 (37.1) | 9,614 (45.0) | 3,140 (14.7) | 694 (3.2) |
| Nepal (2010) | 9,182 (39.0) | 10,864 (46.1) | 2,956 (12.5) | 566 (2.4) |
| Pakistan (2013) | 338 (52.8) | 215 (33.6) | 66 (10.3) | 21 (3.3) |
| Pakistan (2014) | 1,320 (54.7) | 751 (31.1) | 267 (11.1) | 77 (3.2) |
| Papua New Guinea (2009) | 1,136 (60.7) | 423 (22.6) | 289 (15.4) | 23 (1.2) |
| Rwanda (2017) | 1,910 (69.2) | 409 (14.8) | 432 (15.6) | 11 (0.4) |
| South Africa (2016) | 318 (80.7) | 45 (11.4) | 27 (6.9) | 4 (1.0) |
| Sri Lanka (2015) | 9,385 (70.2) | 2,828 (21.1) | 841 (6.3) | 321 (2.4) |
| Tanzania (2001) | 4,843 (63.5) | 1,445 (18.9) | 1,278 (16.7) | 64 (0.8) |
| Tanzania (2008) | 638 (78.0) | 140 (17.1) | 29 (3.5) | 11 (1.3) |
| Tanzania (2010) | 5,012 (60.3) | 2,107 (25.4) | 1,161 (14.0) | 29 (0.3) |
| Tanzania (2014a) | 271 (66.6) | 111 (27.3) | 18 (4.4) | 7 (1.7) |
| Tanzania (2014b) | 1,985 (85.6) | 229 (9.9) | 95 (4.1) | 10 (0.4) |
| Thailand (2000) | 2,966 (77.2) | 532 (13.8) | 326 (8.5) | 20 (0.5) |
| Uganda (2016) | 505 (79.5) | 95 (15.0) | 34 (5.4) | 1 (0.2) |
| Uganda (2018) | 4,935 (78.9) | 834 (13.3) | 436 (7.0) | 50 (0.8) |
| Zambia (2011) | 17,337 (59.4) | 6,398 (21.9) | 5,352 (18.3) | 120 (0.4) |
| Zambia (2013) | 412 (58.6) | 138 (19.6) | 150 (21.3) | 3 (0.4) |
| Zambia (2014) | 573 (75.2) | 138 (18.1) | 46 (6.0) | 5 (0.7) |
| Zambia (2015) | 5,221 (54.9) | 1,765 (18.6) | 2,474 (26.0) | 49 (0.5) |
| Zimbabwe (2012) | 2,581 (61.7) | 635 (15.2) | 925 (22.1) | 41 (1.0) |

| **Study** | **Small types (10 categories)** | **nonSmall types  (10 categories)** | **Small types  (6 categories)** | **nonSmall types  (6 categories)** |
| --- | --- | --- | --- | --- |
| Argentina (2000) | 789 (13.8) | 4,909 (86.2) | 763 (13.4) | 4,935 (86.6) |
| Bangladesh (2001) | 10,807 (80.8) | 2,561 (19.2) | 10,696 (80.0) | 2,672 (20.0) |
| Bangladesh (2007) | 15,181 (74.1) | 5,320 (25.9) | 15,029 (73.3) | 5,472 (26.7) |
| Bangladesh (2011) | 10,884 (60.4) | 7,123 (39.6) | 10,805 (60.0) | 7,202 (40.0) |
| Bangladesh (2014) | 1,300 (50.5) | 1,272 (49.5) | 1,285 (50.0) | 1,287 (50.0) |
| Botswana (2014) | 43,419 (26.5) | 120,509 (73.5) | 42,378 (25.9) | 121,550 (74.1) |
| Brazil (2015) | 820 (19.3) | 3,437 (80.7) | 797 (18.7) | 3,460 (81.3) |
| Burkina Faso (2004) | 429 (41.1) | 616 (58.9) | 424 (40.6) | 621 (59.4) |
| Burkina Faso (2006) | 386 (36.8) | 664 (63.2) | 382 (36.4) | 668 (63.6) |
| China (2002) | 924 (21.1) | 3,456 (78.9) | 919 (21.0) | 3,461 (79.0) |
| China (2012) | 5,669 (13.4) | 36,580 (86.6) | 5,448 (12.9) | 36,801 (87.1) |
| Ethiopia (2017) | 261 (48.0) | 283 (52.0) | 261 (48.0) | 283 (52.0) |
| Ethiopia (2018) | 555 (39.0) | 869 (61.0) | 554 (38.9) | 870 (61.1) |
| Ethiopia (2020) | 245 (44.1) | 311 (55.9) | 244 (43.9) | 312 (56.1) |
| Ghana (2009) | 293 (28.3) | 744 (71.7) | 288 (27.8) | 749 (72.2) |
| Ghana (2013) | 491 (38.0) | 800 (62.0) | 486 (37.6) | 805 (62.4) |
| Guatemala (2013) | 158 (28.0) | 407 (72.0) | 152 (26.9) | 413 (73.1) |
| India (2000) | 2,817 (68.1) | 1,319 (31.9) | 2,798 (67.6) | 1,338 (32.4) |
| India (2010) | 23,760 (52.8) | 21,198 (47.2) | 23,552 (52.4) | 21,406 (47.6) |
| India (2013) | 304 (53.1) | 269 (46.9) | 300 (52.4) | 273 (47.6) |
| India (2016) | 292 (44.7) | 361 (55.3) | 284 (43.5) | 369 (56.5) |
| Malawi (2003) | 360 (30.0) | 839 (70.0) | 358 (29.9) | 841 (70.1) |
| Malawi (2011) | 324 (30.2) | 750 (69.8) | 319 (29.7) | 755 (70.3) |
| Mexico (2017) | 301 (34.0) | 585 (66.0) | 283 (31.9) | 603 (68.1) |
| Nepal (2002) | 13,609 (63.6) | 7,774 (36.4) | 13,448 (62.9) | 7,935 (37.1) |
| Nepal (2010) | 14,557 (61.8) | 9,011 (38.2) | 14,386 (61.0) | 9,182 (39.0) |
| Pakistan (2013) | 313 (48.9) | 327 (51.1) | 302 (47.2) | 338 (52.8) |
| Pakistan (2014) | 1,112 (46.0) | 1,303 (54.0) | 1,095 (45.3) | 1,320 (54.7) |
| Papua New Guinea (2009) | 747 (39.9) | 1,124 (60.1) | 735 (39.3) | 1,136 (60.7) |
| Rwanda (2017) | 857 (31.0) | 1,905 (69.0) | 852 (30.8) | 1,910 (69.2) |
| South Africa (2016) | 76 (19.3) | 318 (80.7) | 76 (19.3) | 318 (80.7) |
| Sri Lanka (2015) | 4,115 (30.8) | 9,260 (69.2) | 3,990 (29.8) | 9,385 (70.2) |
| Tanzania (2001) | 2,791 (36.6) | 4,839 (63.4) | 2,787 (36.5) | 4,843 (63.5) |
| Tanzania (2008) | 181 (22.1) | 637 (77.9) | 180 (22.0) | 638 (78.0) |
| Tanzania (2010) | 3,300 (39.7) | 5,009 (60.3) | 3,297 (39.7) | 5,012 (60.3) |
| Tanzania (2014a) | 138 (33.9) | 269 (66.1) | 136 (33.4) | 271 (66.6) |
| Tanzania (2014b) | 339 (14.6) | 1,980 (85.4) | 334 (14.4) | 1,985 (85.6) |
| Thailand (2000) | 896 (23.3) | 2,948 (76.7) | 878 (22.8) | 2,966 (77.2) |
| Uganda (2016) | 131 (20.6) | 504 (79.4) | 130 (20.5) | 505 (79.5) |
| Uganda (2018) | 1,333 (21.3) | 4,922 (78.7) | 1,320 (21.1) | 4,935 (78.9) |
| Zambia (2011) | 11,924 (40.8) | 17,283 (59.2) | 11,870 (40.6) | 17,337 (59.4) |
| Zambia (2013) | 302 (43.0) | 401 (57.0) | 291 (41.4) | 412 (58.6) |
| Zambia (2014) | 191 (25.1) | 571 (74.9) | 189 (24.8) | 573 (75.2) |
| Zambia (2015) | 4,304 (45.3) | 5,205 (54.7) | 4,288 (45.1) | 5,221 (54.9) |
| Zimbabwe (2012) | 1,608 (38.5) | 2,574 (61.5) | 1,601 (38.3) | 2,581 (61.7) |

**Appendix S7: Table of references for included studies**

| Argentina (2000) | Grandi C, Del Pino M, Casale Aragon D, Dos Santos Rodrigues L, Cunha Cardoso V. Evaluation of the INTERGROWTH-21st project newborn standard for neonatal phenotypes and neonatal morbidity and mortality. Rev Fac Cien Med Univ Nac Cordoba. 2020 May 6;77(2):86-93. doi: 10.31053/1853.0605.v77.n2.28064. PMID: 32558510. |
| --- | --- |
| Bangladesh (2001) | Klemm RD, Merrill RD, Wu L, Shamim AA, Ali H, Labrique A, Christian P, West KP Jr. Low-birthweight rates higher among Bangladeshi neonates measured during active birth surveillance compared to national survey data. Matern Child Nutr. 2015 Oct;11(4):583-94. doi: 10.1111/mcn.12041. Epub 2013 May 6. PMID: 23647669; PMCID: PMC6860210. |
| Bangladesh (2007) | West KP Jr, Shamim AA, Mehra S, Labrique AB, Ali H, Shaikh S, Klemm RD, Wu LS, Mitra M, Haque R, Hanif AA, Massie AB, Merrill RD, Schulze KJ, Christian P. Effect of maternal multiple micronutrient vs iron-folic acid supplementation on infant mortality and adverse birth outcomes in rural Bangladesh: the JiVitA-3 randomized trial. JAMA. 2014 Dec 24-31;312(24):2649-58. doi: 10.1001/jama.2014.16819. PMID: 25536256. |
| Bangladesh (2011) | Saha SK, Schrag SJ, El Arifeen S, Mullany LC, Shahidul Islam M, Shang N, Qazi SA, Zaidi AKM, Bhutta ZA, Bose A, Panigrahi P, Soofi SB, Connor NE, Mitra DK, Isaac R, Winchell JM, Arvay ML, Islam M, Shafiq Y, Nisar I, Baloch B, Kabir F, Ali M, Diaz MH, Satpathy R, Nanda P, Padhi BK, Parida S, Hotwani A, Hasanuzzaman M, Ahmed S, Belal Hossain M, Ariff S, Ahmed I, Ibne Moin SM, Mahmud A, Waller JL, Rafiqullah I, Quaiyum MA, Begum N, Balaji V, Halen J, Nawshad Uddin Ahmed ASM, Weber MW, Hamer DH, Hibberd PL, Sadeq-Ur Rahman Q, Mogan VR, Hossain T, McGee L, Anandan S, Liu A, Panigrahi K, Abraham AM, Baqui AH. Causes and incidence of community-acquired serious infections among young children in south Asia (ANISA): an observational cohort study. Lancet. 2018 Jul 14;392(10142):145-159. doi: 10.1016/S0140-6736(18)31127-9. Epub 2018 Jul 6. PMID: 30025808; PMCID: PMC6053599. |
| Bangladesh (2014) | AMANHI (Alliance for Maternal and Newborn Health Improvement), Baqui A, Ahmed P, Dasgupta SK, Begum N, Rahman M, Islam N, Quaiyum M, Kirkwood B, Edmond K, Shannon C, Newton S, Hurt L, Jehan F, Nisar I, Hussain A, Nadeem N, Ilyas M, Zaidi A, Sazawal S, Deb S, Dutta A, Dhingra U, Ali SM, Hamer DH, Semrau KE, Straszak-Suri M, Grogan C, Bemba G, Lee AC, Wylie BJ, Manu A, Yoshida S, Bahl R. Development and validation of a simplified algorithm for neonatal gestational age assessment - protocol for the Alliance for Maternal Newborn Health Improvement (AMANHI) prospective cohort study. J Glob Health. 2017 Dec;7(2):021201. doi: 10.7189/jogh.07.021201. PMID: 29163937; PMCID: PMC5665676. |
| Botswana (2014) | Zash R, Holmes L, Diseko M, Jacobson DL, Brummel S, Mayondi G, Isaacson A, Davey S, Mabuta J, Mmalane M, Gaolathe T, Essex M, Lockman S, Makhema J, Shapiro RL. Neural-Tube Defects and Antiretroviral Treatment Regimens in Botswana. N Engl J Med. 2019 Aug 29;381(9):827-840. doi: 10.1056/NEJMoa1905230. Epub 2019 Jul 22. PMID: 31329379; PMCID: PMC6995896. |
| Brazil (2015) | Hallal PC, Bertoldi AD, Domingues MR, da Silveira MF, Demarco FF, da Silva ICM, Barros FC, Victora CG, Bassani DG. Cohort Profile: The 2015 Pelotas (Brazil) Birth Cohort Study. Int J Epidemiol. 2018 Aug 1;47(4):1048-1048h. doi: 10.1093/ije/dyx219. PMID: 29126133; PMCID: PMC6124621. |
| Burkina Faso (2004) | Roberfroid D, Huybregts L, Lanou H, Ouedraogo L, Henry MC, Meda N, Kolsteren P; MISAME study group. Impact of prenatal multiple micronutrients on survival and growth during infancy: a randomized controlled trial. Am J Clin Nutr. 2012 Apr;95(4):916-24. doi: 10.3945/ajcn.111.029033. Epub 2012 Feb 29. PMID: 22378724. |
| Burkina Faso (2006) | Huybregts L, Roberfroid D, Lanou H, Menten J, Meda N, Van Camp J, Kolsteren P. Prenatal food supplementation fortified with multiple micronutrients increases birth length: a randomized controlled trial in rural Burkina Faso. Am J Clin Nutr. 2009 Dec;90(6):1593-600. doi: 10.3945/ajcn.2009.28253. Epub 2009 Oct 7. PMID: 19812173. |
| China (2002) | Zeng L, Dibley MJ, Cheng Y, Dang S, Chang S, Kong L, Yan H. Impact of micronutrient supplementation during pregnancy on birth weight, duration of gestation, and perinatal mortality in rural western China: double blind cluster randomised controlled trial. BMJ. 2008 Nov 7;337:a2001. doi: 10.1136/bmj.a2001. Erratum in: BMJ. 2008;337:a2522. PMID: 18996930; PMCID: PMC2577799. |
| China (2012) | Qiu X, Lu JH, He JR, Lam KH, Shen SY, Guo Y, Kuang YS, Yuan MY, Qiu L, Chen NN, Lu MS, Li WD, Xing YF, Zhou FJ, Bartington S, Cheng KK, Xia HM. The Born in Guangzhou Cohort Study (BIGCS). Eur J Epidemiol. 2017 Apr;32(4):337-346. doi: 10.1007/s10654-017-0239-x. Epub 2017 Mar 20. PMID: 28321694. |
| Ethiopia (2017) | Tesfamariam K, Argaw A, Hanley-Cook GT, Gebreyesus SH, Kolsteren P, Belachew T, Van de Velde M, De Saeger S, De Boevre M, Lachat C. Multiple mycotoxin exposure during pregnancy and risks of adverse birth outcomes: a prospective cohort study in rural Ethiopia. Environ Int. 2022 Feb;160:107052. doi: 10.1016/j.envint.2021.107052. Epub 2021 Dec 21. PMID: 34952355. |
| Ethiopia (2018) | Chan GJ, Goddard FGB, Hunegnaw BM, Mohammed Y, Hunegnaw M, Haneuse S, Bekele C, Bekele D. Estimates of Stillbirths, Neonatal Mortality, and Medically Vulnerable Live Births in Amhara, Ethiopia. JAMA Netw Open. 2022 Jun 1;5(6):e2218534. doi: 10.1001/jamanetworkopen.2022.18534. PMID: 35749113; PMCID: PMC9233235. |
| Ethiopia (2020) | Lee AC, Abate FW, Mullany LC, Baye E, Berhane YY, Derebe MM, Eglovitch M, Fasil N, Olson IE, Kidane WT, Shiferaw T, Shiferie F, Tsegaye F, Tsegaye S, Yibeltal K, Chan GJ, Christian P, Isanaka S, Kang Y, Lu C, Mengistie MM, Molina RL, Stojanov MD, Van Dyk F, Tadesse AW, Wondale AT, Wylie BJ, Worku A, Berhane Y. Enhancing Nutrition and Antenatal Infection Treatment (ENAT) study: protocol of a pragmatic clinical effectiveness study to improve birth outcomes in Ethiopia. BMJ Paediatr Open. 2022 Jan;6(1):e001327. doi: 10.1136/bmjpo-2021-001327. PMID: 36053580; PMCID: PMC8762145. |
| Ghana (2009) | Adu-Afarwuah S, Lartey A, Okronipa H, Ashorn P, Zeilani M, Peerson JM, Arimond M, Vosti S, Dewey KG. Lipid-based nutrient supplement increases the birth size of infants of primiparous women in Ghana. Am J Clin Nutr. 2015 Apr;101(4):835-46. doi: 10.3945/ajcn.114.091546. Epub 2015 Feb 11. PMID: 25833980. |
| Ghana (2013) | Jack DW, Asante KP, Wylie BJ, Chillrud SN, Whyatt RM, Ae-Ngibise KA, Quinn AK, Yawson AK, Boamah EA, Agyei O, Mujtaba M, Kaali S, Kinney P, Owusu-Agyei S. Ghana randomized air pollution and health study (GRAPHS): study protocol for a randomized controlled trial. Trials. 2015 Sep 22;16:420. doi: 10.1186/s13063-015-0930-8. PMID: 26395578; PMCID: PMC4579662. |
| Guatemala (2013) | Hambidge KM, Krebs NF, Westcott JE, Garces A, Goudar SS, Kodkany BS, Pasha O, Tshefu A, Bose CL, Figueroa L, Goldenberg RL, Derman RJ, Friedman JE, Frank DN, McClure EM, Stolka K, Das A, Koso-Thomas M, Sundberg S; Preconception Trial Group. Preconception maternal nutrition: a multi-site randomized controlled trial. BMC Pregnancy Childbirth. 2014 Mar 20;14:111. doi: 10.1186/1471-2393-14-111. PMID: 24650219; PMCID: PMC4000057. |
| India (2000) | Rahmathullah L, Tielsch JM, Thulasiraj RD, Katz J, Coles C, Devi S, John R, Prakash K, Sadanand AV, Edwin N, Kamaraj C. Impact of supplementing newborn infants with vitamin A on early infant mortality: community based randomised trial in southern India. BMJ. 2003 Aug 2;327(7409):254. doi: 10.1136/bmj.327.7409.254. PMID: 12896935; PMCID: PMC167159. |
| India (2010) | Mazumder S, Taneja S, Bhatia K, Yoshida S, Kaur J, Dube B, Toteja GS, Bahl R, Fontaine O, Martines J, Bhandari N; Neovita India Study Group. Efficacy of early neonatal supplementation with vitamin A to reduce mortality in infancy in Haryana, India (Neovita): a randomised, double-blind, placebo-controlled trial. Lancet. 2015 Apr 4;385(9975):1333-42. doi: 10.1016/S0140-6736(14)60891-6. Epub 2014 Dec 11. PMID: 25499546. |
| India (2013) | Hambidge KM, Krebs NF, Westcott JE, Garces A, Goudar SS, Kodkany BS, Pasha O, Tshefu A, Bose CL, Figueroa L, Goldenberg RL, Derman RJ, Friedman JE, Frank DN, McClure EM, Stolka K, Das A, Koso-Thomas M, Sundberg S; Preconception Trial Group. Preconception maternal nutrition: a multi-site randomized controlled trial. BMC Pregnancy Childbirth. 2014 Mar 20;14:111. doi: 10.1186/1471-2393-14-111. PMID: 24650219; PMCID: PMC4000057. |
| India (2016) | Babu GR, Murthy GVS, Reddy Y, Deepa R, Yamuna A, Prafulla S, Krishnan A, Lobo E, Rathnaiah M, Kinra S. Small for gestational age babies and depressive symptoms of mothers during pregnancy: Results from a birth cohort in India. Wellcome Open Res. 2020 Feb 6;3:76. doi: 10.12688/wellcomeopenres.14618.3. PMID: 31828224; PMCID: PMC6892423. |
| Malawi (2003) | Luntamo M, Kulmala T, Mbewe B, Cheung YB, Maleta K, Ashorn P. Effect of repeated treatment of pregnant women with sulfadoxine-pyrimethamine and azithromycin on preterm delivery in Malawi: a randomized controlled trial. Am J Trop Med Hyg. 2010 Dec;83(6):1212-20. doi: 10.4269/ajtmh.2010.10-0264. PMID: 21118924; PMCID: PMC2990034. |
| Malawi (2011) | Ashorn P, Alho L, Ashorn U, Cheung YB, Dewey KG, Harjunmaa U, Lartey A, Nkhoma M, Phiri N, Phuka J, Vosti SA, Zeilani M, Maleta K. The impact of lipid-based nutrient supplement provision to pregnant women on newborn size in rural Malawi: a randomized controlled trial. Am J Clin Nutr. 2015 Feb;101(2):387-97. doi: 10.3945/ajcn.114.088617. Epub 2014 Dec 10. PMID: 25646337. |
| Mexico (2017) | Mendoza-Carrera CE, Acevedo-Gallegos S, Lumbreras-Márquez M, Gallardo-Gaona JM, Copado-Mendoza DY, Rodriguez-Sibaja MJ. Comparación de cuatro tablas de crecimiento fetal para la predicción de desenlaces perinatales adversos en un hospital de tercer nivel de México. Ginecol. obstet. Méx.  [revista en la Internet]. 2021;89( 9 ):704-714. Disponible en: http://www.scielo.org.mx/scielo.php?script=sci_arttext&pid=S0300-90412021000900704&lng=es.  Epub 04-Abr-2022.  <https://doi.org/10.24245/gom.v89i9.5817>. |
| Nepal (2002) | Mullany LC, Darmstadt GL, Khatry SK, Katz J, LeClerq SC, Shrestha S, Adhikari R, Tielsch JM. Topical applications of chlorhexidine to the umbilical cord for prevention of omphalitis and neonatal mortality in southern Nepal: a community-based, cluster-randomised trial. Lancet. 2006 Mar 18;367(9514):910-8. doi: 10.1016/S0140-6736(06)68381-5. PMID: 16546539; PMCID: PMC2367116. |
| Nepal (2010) | N/A |
| Pakistan (2013) | Hambidge KM, Krebs NF, Westcott JE, Garces A, Goudar SS, Kodkany BS, Pasha O, Tshefu A, Bose CL, Figueroa L, Goldenberg RL, Derman RJ, Friedman JE, Frank DN, McClure EM, Stolka K, Das A, Koso-Thomas M, Sundberg S; Preconception Trial Group. Preconception maternal nutrition: a multi-site randomized controlled trial. BMC Pregnancy Childbirth. 2014 Mar 20;14:111. doi: 10.1186/1471-2393-14-111. PMID: 24650219; PMCID: PMC4000057. |
| Pakistan (2014) | AMANHI (Alliance for Maternal and Newborn Health Improvement), Baqui A, Ahmed P, Dasgupta SK, Begum N, Rahman M, Islam N, Quaiyum M, Kirkwood B, Edmond K, Shannon C, Newton S, Hurt L, Jehan F, Nisar I, Hussain A, Nadeem N, Ilyas M, Zaidi A, Sazawal S, Deb S, Dutta A, Dhingra U, Ali SM, Hamer DH, Semrau KE, Straszak-Suri M, Grogan C, Bemba G, Lee AC, Wylie BJ, Manu A, Yoshida S, Bahl R. Development and validation of a simplified algorithm for neonatal gestational age assessment - protocol for the Alliance for Maternal Newborn Health Improvement (AMANHI) prospective cohort study. J Glob Health. 2017 Dec;7(2):021201. doi: 10.7189/jogh.07.021201. PMID: 29163937; PMCID: PMC5665676. |
| Papua New Guinea (2009) | Unger HW, Ome-Kaius M, Wangnapi RA, Umbers AJ, Hanieh S, Suen CS, Robinson LJ, Rosanas-Urgell A, Wapling J, Lufele E, Kongs C, Samol P, Sui D, Singirok D, Bardaji A, Schofield L, Menendez C, Betuela I, Siba P, Mueller I, Rogerson SJ. Sulphadoxine-pyrimethamine plus azithromycin for the prevention of low birthweight in Papua New Guinea: a randomised controlled trial. BMC Med. 2015 Jan 16;13:9. doi: 10.1186/s12916-014-0258-3. PMID: 25591391; PMCID: PMC4305224. |
| Rwanda (2017) | Sayinzoga F, Lundeen T, Musange SF, Butrick E, Nzeyimana D, Murindahabi N, Azman-Firdaus H, Sloan NL, Benitez A, Phillips B, Ghosh R, Walker D. Assessing the impact of group antenatal care on gestational length in Rwanda: A cluster-randomized trial. PLoS One. 2021 Feb 2;16(2):e0246442. doi: 10.1371/journal.pone.0246442. PMID: 33529256; PMCID: PMC7853466. |
| South Africa (2016) | N/A |
| Sri Lanka (2015) | Senanayake H, Piccoli M, Valente EP, Businelli C, Mohamed R, Fernando R, Sakalasuriya A, Ihsan FR, Covi B, Wanzira H, Lazzerini M. Implementation of the WHO manual for Robson classification: an example from Sri Lanka using a local database for developing quality improvement recommendations. BMJ Open. 2019 Feb 19;9(2):e027317. doi: 10.1136/bmjopen-2018-027317. |
| Tanzania (2001) | Fawzi WW, Msamanga GI, Urassa W, Hertzmark E, Petraro P, Willett WC, Spiegelman D. Vitamins and perinatal outcomes among HIV-negative women in Tanzania. N Engl J Med. 2007 Apr 5;356(14):1423-31. doi: 10.1056/NEJMoa064868. PMID: 17409323. |
| Tanzania (2008) | Schmiegelow C, Minja D, Oesterholt M, Pehrson C, Suhrs HE, Boström S, Lemnge M, Magistrado P, Rasch V, Lusingu J, Theander TG, Bruun Nielsen B. Factors associated with and causes of perinatal mortality in northeastern Tanzania. Acta Obstet Gynecol Scand. 2012 Sep;91(9):1061-8. doi: 10.1111/j.1600-0412.2012.01478.x. PMID: 22676243. |
| Tanzania (2010) | Masanja H, Smith ER, Muhihi A, Briegleb C, Mshamu S, Ruben J, Noor RA, Khudyakov P, Yoshida S, Martines J, Bahl R, Fawzi WW; Neovita Tanzania Study Group. Effect of neonatal vitamin A supplementation on mortality in infants in Tanzania (Neovita): a randomised, double-blind, placebo-controlled trial. Lancet. 2015 Apr 4;385(9975):1324-32. doi: 10.1016/S0140-6736(14)61731-1. Epub 2014 Dec 11. PMID: 25499543; PMCID: PMC4419827. |
| Tanzania (2014a) | Hjort L, Lykke Møller S, Minja D, Msemo O, Nielsen BB, Lund Christensen D, Theander T, Nielsen K, Larsen LG, Grunnet LG, Groop L, Prasad R, Lusingu J, Schmiegelow C, Bygbjerg IC. FOETAL for NCD-FOetal Exposure and Epidemiological Transitions: the role of Anaemia in early Life for Non-Communicable Diseases in later life: a prospective preconception study in rural Tanzania. BMJ Open. 2019 May 22;9(5):e024861. doi: 10.1136/bmjopen-2018-024861. Erratum in: BMJ Open. 2019 Jun 28;9(6):e024861corr1. PMID: 31122967; PMCID: PMC6537995. |
| Tanzania (2014b) | AMANHI (Alliance for Maternal and Newborn Health Improvement), Baqui A, Ahmed P, Dasgupta SK, Begum N, Rahman M, Islam N, Quaiyum M, Kirkwood B, Edmond K, Shannon C, Newton S, Hurt L, Jehan F, Nisar I, Hussain A, Nadeem N, Ilyas M, Zaidi A, Sazawal S, Deb S, Dutta A, Dhingra U, Ali SM, Hamer DH, Semrau KE, Straszak-Suri M, Grogan C, Bemba G, Lee AC, Wylie BJ, Manu A, Yoshida S, Bahl R. Development and validation of a simplified algorithm for neonatal gestational age assessment - protocol for the Alliance for Maternal Newborn Health Improvement (AMANHI) prospective cohort study. J Glob Health. 2017 Dec;7(2):021201. doi: 10.7189/jogh.07.021201. PMID: 29163937; PMCID: PMC5665676. |
| Thailand (2000) | Isaranurug S, Mo-suwan L, Choprapawon C. A population-based cohort study of effect of maternal risk factors on low birthweight in Thailand. J Med Assoc Thai. 2007 Dec;90(12):2559-64. PMID: 18386704. |
| Uganda (2016) | Kajubi R, Ochieng T, Kakuru A, Jagannathan P, Nakalembe M, Ruel T, Opira B, Ochokoru H, Ategeka J, Nayebare P, Clark TD, Havlir DV, Kamya MR, Dorsey G. Monthly sulfadoxine-pyrimethamine versus dihydroartemisinin-piperaquine for intermittent preventive treatment of malaria in pregnancy: a double-blind, randomised, controlled, superiority trial. Lancet. 2019 Apr 6;393(10179):1428-1439. doi: 10.1016/S0140-6736(18)32224-4. Epub 2019 Mar 22. PMID: 30910321. |
| Uganda (2018) | Mulowooza J, Santos N, Isabirye N, Inhensiko I, Sloan NL, Shah S, Butrick E, Waiswa P, Walker D. Midwife-performed checklist and ultrasound to identify obstetric conditions at labour triage in Uganda: A quasi-experimental study. Midwifery. 2021 May;96:102949. doi: 10.1016/j.midw.2021.102949. Epub 2021 Feb 12. PMID: 33631411; PMCID: PMC7988503. |
| Zambia (2011) | Semrau KEA, Herlihy J, Grogan C, Musokotwane K, Yeboah-Antwi K, Mbewe R, Banda B, Mpamba C, Hamomba F, Pilingana P, Zulu A, Chanda-Kapata P, Biemba G, Thea DM, MacLeod WB, Simon JL, Hamer DH. Effectiveness of 4% chlorhexidine umbilical cord care on neonatal mortality in Southern Province, Zambia (ZamCAT): a cluster-randomised controlled trial. Lancet Glob Health. 2016 Nov;4(11):e827-e836. doi: 10.1016/S2214-109X(16)30215-7. Epub 2016 Sep 29. PMID: 27693439. |
| Zambia (2013) | Chaponda EB, Chico RM, Bruce J, Michelo C, Vwalika B, Mharakurwa S, Chaponda M, Chipeta J, Chandramohan D. Malarial infection and curable sexually transmitted and reproductive tract infections among pregnant women in a rural district of Zambia. Am J Trop Med Hyg. 2016;95(5):1069-1076. doi: 10.4269/ajtmh.16-0370. PMID: 27672205 PMCID: PMC5094219 |
| Zambia (2014) | AMANHI (Alliance for Maternal and Newborn Health Improvement), Baqui A, Ahmed P, Dasgupta SK, Begum N, Rahman M, Islam N, Quaiyum M, Kirkwood B, Edmond K, Shannon C, Newton S, Hurt L, Jehan F, Nisar I, Hussain A, Nadeem N, Ilyas M, Zaidi A, Sazawal S, Deb S, Dutta A, Dhingra U, Ali SM, Hamer DH, Semrau KE, Straszak-Suri M, Grogan C, Bemba G, Lee AC, Wylie BJ, Manu A, Yoshida S, Bahl R. Development and validation of a simplified algorithm for neonatal gestational age assessment - protocol for the Alliance for Maternal Newborn Health Improvement (AMANHI) prospective cohort study. J Glob Health. 2017 Dec;7(2):021201. doi: 10.7189/jogh.07.021201. PMID: 29163937; PMCID: PMC5665676. |
| Zambia (2015) | N/A |
| Zimbabwe (2012) | Humphrey JH, Mbuya MNN, Ntozini R, Moulton LH, Stoltzfus RJ, Tavengwa NV, Mutasa K, Majo F, Mutasa B, Mangwadu G, Chasokela CM, Chigumira A, Chasekwa B, Smith LE, Tielsch JM, Jones AD, Manges AR, Maluccio JA, Prendergast AJ; Sanitation Hygiene Infant Nutrition Efficacy (SHINE) Trial Team. Independent and combined effects of improved water, sanitation, and hygiene, and improved complementary feeding, on child stunting and anaemia in rural Zimbabwe: a cluster-randomised trial. Lancet Glob Health. 2019 Jan;7(1):e132-e147. doi: 10.1016/S2214-109X(18)30374-7. PMID: 30554749; PMCID: PMC6293965. |

**Appendix S8: Acknowledgments**

| Argentina (2000) | Marcelo San Pedro, former CEO EPSON Latin America; Miguel Larguía, former head, Department of Pediatrics, Hospital Materno Infantil Ramón Sardá, Buenos Aires, Argentina |
| --- | --- |
| Bangladesh (2001) | Ellen Piwoz, PhD, former Senior Program Officer, Bill & Melinda Gates Foundation, Seattle, WA; Kelsey Alland, Research Associate II, Center for Human Nutrition, Dept of International Health, Johns Hopkins Bloomberg School of Public Health |
| Bangladesh (2007) | Ellen Piwoz, PhD, former Senior Program Officer, Bill & Melinda Gates Foundation, Seattle, WA; Kelsey Alland, Research Associate II, Center for Human Nutrition, Dept of International Health, Johns Hopkins Bloomberg School of Public Health |
| Botswana (2014) | Molly Pretorius Holme, Harvard TH Chan School of Public Health; Lewis Holmes, Massachusetts General Hospital |
| Burkina Faso (2004) | Hermann Lanou, PhD, Unité Nutrition et Maladies Métaboliques, Institut de Recherche en Sciences de la Santé, Bobo-Dioulasso, Burkina Faso |
| Burkina Faso (2006) | Laeticia Celine Toe, MD, Department of Food Technology, Safety and Health, Faculty of Bioscience Engineering, Ghent University, Ghent, Belgium, Unité Nutrition et Maladies Métaboliques, Institut de Recherche en Sciences de la Santé, Bobo-Dioulasso, Burkina Faso |
| China (2002) | Michael J Dibley, MPH, Prof., The Sydney School of Public Health, Faculty of Medicine, The University of Sydney, NSW, Australia |
| Ethiopia (2017) | Addis Ababa University Thematic Research Grant |
| Ethiopia (2018) | Yahya Mohammed, St. Paul's Hospital Millennium Medical College; Mesfin Hunegnaw, St. Paul's Hospital Millennium Medical College; Chalachew Bekele, St. Paul's Hospital Millennium Medical College |
| Ethiopia (2020) | Yemane Berhane; Alemayehu Worku, Luke C Mullany, Michelle Eglovitch, Workagegnehu Tarekegn Kidane, Sitota Tsegaye, Kalkidan Yibeltal, Ingrid Olson, Grace J Chan, Parul Christian, Sheila Isanaka, Yunhee Kang, Rose L. Molina, Amare Worku Tadesse, Blair J Wylie |
| Ghana (2009) | Harriet Okronipa, PhD, Institute for Global Nutrition, Department of Nutrition, University of California, Davis, CA, USA, heokronipa@ucdavis.edu; Anna Lartey (Ph.D.); Professor; Department of Nutrition and Food Science, University of Ghana, Legon, Accra, Ghana; Email: aalartey@gmail.com |
| India (2010) | Dr. Sarmila Mazumder, PhD, Senior Scientist, Centre For Health Research and Development, Society for Applied Studies; Dr. Nita Bhandari, PhD, Senior Scientist, Centre For Health Research and Development, Society for Applied Studies, Dr. Rajiv Bahl, PhD, Newborn Unit Head and Head of Research, Department of Maternal, Newborn, Child and Adolescent Health and Ageing, World Health Organization, Geneva, Switzerland, Dr. Jose Martines, MD, Centre for Intervention Science in Maternal and Child Health (CISMAC), Centre for International Health, University of Bergen, Bergen, Norway. |
| India (2016) | Dr Suresh Shapeti  Special Officer and Director(In-charge)  Indian Institute of Public Health,  Public Health Foundation of India-Bengaluru-560023   The team also acknowledges the Directorate of Health and Family Welfare, Government of Karnataka for providing the approval for conducting the study. |
| Papua New Guinea (2009) | Dr Sarah Hanieh, Department of Infectious Diseases, University of Melbourne, Doherty Institute, Melbourne Australia |
| Rwanda (2017) | David Nzeyimana, MSc, Progam Manager, Preterm Birth Initiative - Rwanda, dnzeyimana@nursph.org; Nathalie Murindahabi, MSc, Data Manager, Preterm Birth Initiative - Rwanda, nmulindahabi@nursph.org (RWANDA dataset) |
| South Africa (2016) | David Sanders, University of Western Cape; Rina Swart, University of Western Cape; Tanya Doherty, South African Medical Research Council |
| Tanzania (2008) | Dr. Birgitte Bruun Nielsen, MD, PhD, Department of Obstetrics, Copenhagen University Hospital, Denmark, birgitte.bruun.nielsen@dadlnet.dk |
| Tanzania (2014a) | Dr. Birgitte Bruun Nielsen, MD, PhD, Department of Obstetrics, Copenhagen University Hospital, Denmark, birgitte.bruun.nielsen@dadlnet.dk |
| Thailand (2000) | Dr Chanpen Choprapawon, MD., MSC. (Epidemiology)  Retirement from Ministry of Public Health (MOPH), Thailand |
| Uganda (2016) | PROMOTE Birth Cohort 3 team and in particular Grant Dorsey, Department of Medicine, University of California San Francisco, California, USA; Teddy Andra, Infectious Diseases Research Collaboration, Kampala, Uganda; Bishop Opira, Infectious Diseases Research Collaboration, Kampala, Uganda; |
| Uganda (2018) | Nathan Isabirye MPH, Program Manager, PTBi Uganda Ultrasound Study isabiryenathan5@gmail.com; Innocent Inhensiko, Data Manager, PTBi Uganda Ultrasound Study, innoinhe23@gmail.com |
| Zambia (2011) | We acknowledge the support from the Zambian Ministry of Health, Southern Province Medical Office, District Medical Offices, and Chiefs and Chieftainesses of Southern Province for study implementation from 2009-2014. Finally, we thank the women, their newborn babies, and their families for their participation and dedication to the ZamCAT study. |
| Zambia (2015) | Martha Mwendafilumba, Centre for Infectious Disease Research in Zambia; Herbert Kapesa, Centre for Infectious Disease Research in Zambia |
